# Long-term Survival Analysis From PERLA, A Phase II Randomized Trial of Dostarlimab With Chemotherapy Versus Pembrolizumab With Chemotherapy in Metastatic Nonsquamous NSCLC

**DOI:** 10.1016/j.jtocrr.2025.100900

**Published:** 2025-09-04

**Authors:** Sun Min Lim, Ana Laura Ortega Granados, Gustavo dix Junqueira Pinto, Christian Sebastián Fuentes, Giuseppe Lo Russo, Michael Schenker, Jin Seok Ahn, Filippo de Marinis, Kenneth Locke, Zsolt Szijgyarto, Elena Buss, Neda Stjepanovic, Ivan Diaz-Padilla, Solange Peters

**Affiliations:** a Division of Medical Oncology, Department of Internal Medicine, Yonsei Cancer Center, Severance Hospital, Yonsei University College of Medicine, Seoul, South Korea; b Medical Oncology Department, Hospital Universitario de Jaén, Jaén, Spain; c Department of Medical Oncology, Barretos Cancer Hospital, Rua Antenor Duarte Villela, São Paulo, Brazil; d Centro de Investigaciones Clínicas, FUNDACIÓN RESPIRAR, Buenos Aires, Argentina; e Medical Oncology Department, Thoracic Unit, Fondazione IRCCS Istituto Nazionale dei Tumori, Milan, Italy; f Oncologie Medicala, Policlinica Sf. Nectarie, Centrul de Oncologie, Craiova, Romania; g Division of Hematology-Oncology, Department of Medicine, Samsung Medical Center, Sungkyunkwan University, Seoul, South Korea; h Division of Thoracic Oncology, Istituto Europeo di Oncologia (IRCCS), Milan, Italy; i Oncology Biostatistics, GSK, Philadelphia, Pennsylvania; j Oncology Biostatistics, GSK, London, United Kingdom; k Oncology Clinical Development, GSK, Baar, Switzerland; l Oncology Department, Centre Hospitalier Universitaire Vaudois, Lausanne University, Lausanne, Switzerland

**Keywords:** Dostarlimab, Pembrolizumab, NSCLC, PD-1 receptor, Immune checkpoint inhibitors

## Abstract

**Introduction:**

PERLA is a global, double-blind, phase II trial comparing anti–programmed cell death protein 1 antibodies, dostarlimab, and pembrolizumab in combination with chemotherapy (D+CT and P+CT, respectively) in patients with metastatic nonsquamous NSCLC without actionable genomic aberrations in the first-line setting.

**Methods:**

Patients were randomized 1:1 to receive not more than 35 cycles of 500 mg dostarlimab or 200 mg pembrolizumab, with less than or equal to 35 cycles of 500 mg/m^2^ pemetrexed and less than or equal to 4 cycles of cisplatin (75 mg/m^2^) or carboplatin (area under the curve 5 mg/mL/min) every 3 weeks. The primary end point was the overall response rate by blinded independent central review. The secondary end points included progression-free survival (PFS) on the basis of investigator assessment, overall survival (OS), and safety. Here, we reported on the long-term OS, PFS, and safety analyses.

**Results:**

At the end of the study (September 10, 2024), the median follow-up time (mo) for PFS was 30.4 for D+CT and 30.4 for P+CT. The median PFS (mo [95% confidence interval (CI)]) was 8.8 (6.9–11.0) for D+CT and 6.8 (4.9–7.1) for P+CT (hazard ratio 0.77 [95% CI: 0.58–1.03] at 79% maturity). The median follow-up time (mo) for OS was 35.5 for D+CT and 35.2 for P+CT. The median OS (mo [95% CI]) was 20.2 (14.5–27.3) and 15.9 (11.6–19.3), respectively (hazard ratio 0.75 [95% CI: 0.55–1.02] at 70% maturity). Safety profiles were similar between arms and consistent with previous analyses.

**Conclusions:**

This long-term analysis reaffirms previous observations that D+CT exhibited similar efficacy to P+CT and exhibits strong clinical efficacy as a first-line treatment for patients with metastatic nonsquamous NSCLC.

**Clinical trial registration:**

NCT04581824.

## Introduction

NSCLC has a poor prognosis, particularly for patients with advanced or metastatic disease.^1^ In recent years, the NSCLC treatment landscape has been transformed with the introduction of targeted treatments and immunotherapies, such as programmed cell death receptor-(ligand)1 (PD-[L]1) inhibitors, in the first-line setting.^2^ Dostarlimab is an anti–programmed cell death protein 1 (PD-1) monoclonal antibody the U.S. Food and Drug Administration approved as a single agent for patients with mismatch repair deficient recurrent or advanced solid tumors and in combination with chemotherapy in patients with primary advanced or recurrent endometrial cancer, regardless of mismatch repair status.^3^ PERLA (NCT04581824) is the first global, randomized, phase II trial comparing the efficacy and safety of two anti–PD-1 antibodies, dostarlimab and pembrolizumab, in combination with chemotherapy (D+CT and P+CT, respectively) as first-line treatment in patients with metastatic nonsquamous NSCLC.^4^ Previous analyses of PERLA revealed a confirmed overall response rate (ORR) by blinded independent central review (BICR) of 46% and 37% at the primary analysis (data cutoff [DCO] August 4, 2022) and 45% and 39% at the first supplementary analysis (SA1) (DCO July 7, 2023) in D+CT and P+CT arms, respectively. The median progression-free survival (PFS) at the primary analysis was 8.8 months (95% confidence interval [CI]: 6.7–10.4) and 6.7 months (95% CI: 4.9–7.1), respectively (hazard ratio [HR]: 0.70 [95% CI: 0.50–0.98]; 57% maturity).^4^ Overall survival (OS) analyses at SA1 revealed a numerical trend favoring D+CT versus P+CT (median 19.4 months [95% CI: 14.5–not reached] versus 15.9 months [95% CI: 11.6–19.3]; HR: 0.75 [95% CI: 0.53–1.05]; 55% maturity),^5^ which was maintained at SA2 (DCO April 30, 2024; 20.2 mo [95% CI: 14.5–27.3] versus 15.9 mo [95% CI: 11.6–19.3]; HR: 0.74 [95% CI: 0.54–1.00]; 67% maturity).^4^, ^5^, ^6^ Throughout the primary analysis, SA1, and SA2, safety profiles of the two treatment arms were similar, manageable, and consistent with previously reported data.^7^ However, longer-term survival data are warranted to further evaluate and support dostarlimab as a backbone for combination therapies in patients with NSCLC.

Here, we report PFS, OS, and safety results from PERLA (last patient last visit [LPLV] September 10, 2024), 2.5 years after the last patient’s enrollment.

## Material and Methods

The full PERLA (NCT04581824) trial design has been previously reported.^4^ All patients provided written informed consent before participation in the study, which was conducted in accordance with the Declaration of Helsinki and International Ethical Guidelines, International Council for Harmonization Good Clinical Practice guidelines, and all local laws. Briefly, eligible patients were 18 years of age and older with confirmed metastatic nonsquamous NSCLC, measurable disease per Response Evaluation Criteria in Solid Tumors version 1.1, no actionable genomic aberrations, documented PD-L1 status, Eastern Cooperative Oncology Group (ECOG) performance status score 0 to 1, and no previous systemic therapy for metastatic NSCLC or previous immunotherapy. Eligible patients were randomized 1:1 to receive a combination of four cycles of investigator’s choice of either cisplatin (75 mg/m^2^ intravenous [IV] every 3 wk [Q3W]) or carboplatin (area under the curve 5 mg/mL/min IV Q3W) with pemetrexed (500 mg/m^2^ IV Q3W) combined with either 500 mg dostarlimab or 200 mg pembrolizumab, Q3W for up to 35 cycles (approximately 24 mo) or until disease progression, withdrawal of consent, unacceptable toxicity, or death. Randomization was stratified by PD-L1 status (tumor proportion score [TPS] <1% versus 1%–49% versus^3^ 50%) and smoking status (never versus former or current smoker). The primary end point, confirmed ORR (BICR per Response Evaluation Criteria in Solid Tumors version 1.1 criteria), was reported previously and is not reported here. PFS assessed by investigators and OS were secondary end points. The analyses of OS by PD-L1 subgroups were also performed.

The planned sample size was 240 patients (120 patients per arm), providing 85% power to detect a 15% difference in ORR between the two arms at the 10% one-sided type I error rate, assuming the true ORR is 45% for both arms. Although the study was designed on the basis of a noninferiority trial design framework, because of the large noninferiority margin and type I error rate, precluding a hypothesis of true noninferiority, the prespecified hypothesis was that D+CT and P+CT were “similar” with “similarity” being the ORR for D+CT not being more than 15% lower than the ORR for P+CT. No formal statistical hypothesis testing was planned to be conducted.

## Results

### Study Population and Baseline Characteristics

Between November 19, 2020 and February 18, 2022, a total of 243 patients were recruited globally. Baseline characteristics are shown in Table 1. Briefly, more patients in the D+CT arm had an ECOG performance status of 1, and brain and liver metastases, than in the P+CT arm (69% versus 59%; 18% versus 12%; 16% versus 11%, respectively).

**Table 1:** Patient Demographics and Baseline Characteristics

Variable	D+CT (N = 121)	P+CT (N = 122)
Median age, y (range)	64 (25–80)	65 (46–86)
Age group (y), n (%)		
<65	65 (54)	57 (47)
≥65	56 (46)	65 (53)
Sex, n (%)		
Male	85 (70)	77 (63)
Female	36 (30)	45 (37)
Ethnicity, n (%)		
Hispanic or Latino	25 (21)	32 (26)
Other	90 (74)	84 (69)
Not reported^a^/Unknown^b^	6 (5)	6 (5)
Race, n (%)		
White	87 (72)	84 (69)
Asian	23 (19)	21 (17)
Deidentified	11 (9)	17 (14)
Enrollment region^c^, n (%)		
Europe	62 (51)	65 (53)
South America	35 (29)	34 (28)
Deidentified^d^	24 (20)	23 (19)
Smoking status^e^, n (%)		
Never smoked	17 (14)	17 (14)
Former or current smoker	104 (86)	105 (86)
ECOG performance status, n (%)		
0	37 (31)	50 (41)
1	84 (69)	72 (59)
Stage at initial diagnosis^f^, n (%)		
I	11 (9)	9 (7)
II	2 (2)	3 (2)
III	4 (3)	9 (7)
IV	101 (83)	100 (82)
Unknown^b^	3 (2)	1 (<1)
Histologic subtype, n (%)		
Nonsquamous	117 (97)	121 (>99)
Mixed	4 (3)^g^	1 (<1)^h^
PD-L1 status^e^, n (%)		
TPS <1%	50 (41)	51 (42)
TPS ≥1%	71 (59)	71 (58)
TPS 1–49%	44 (36)	44 (36)
TPS ≥50%	27 (22)	27 (22)
Metastases at baseline, n (%)		
Bone	39 (32)	34 (28)
Brain	22 (18)	15 (12)
Liver	19 (16)	14 (11)

D+CT, dostarlimab with chemotherapy; ECOG, Eastern Cooperative Oncology Group; ITT, intention-to-treat; P+CT, pembrolizumab with chemotherapy; PD-L1, programmed cell death ligand-1; TPS, tumor proportion score.

a Not reported indicates cases in which a patient prefers not to provide their ethnicity or in which the collection of this data is not permitted according to local regulations.

b Unknown indicates cases in which these data are not known.

c Europe: France, Germany, Italy, Poland, Romania, Spain; South America: Argentina, Brazil, Chile.

d Data deidentified for demographic variables if at least one treatment arm had a cell count less than 11; study site locations are described in Lim et al. Nat Commun 2023;14:7301.

e Randomization factors on the basis of data collected in the Interactive Response Technology at randomization.

f Patients are required to have metastatic NSCLC at enrollment.

g Predominantly nonsquamous histologic subtype without small cell component (n = 2) and other (n = 2).

h Predominantly nonsquamous histologic subtype without small cell component.

### Treatment Exposure

The median number of cycles (range) of immunotherapy was 13.0 (1‒35) for D+CT and 7.5 (1‒35) for P+CT. The median number of cycles (range) of pemetrexed was 11.0 (1‒35) with D+CT and 7.0 (1‒35) with P+CT. For platinum exposure, the median number of cycles (range) was 4.0 (1‒4) cycles for both carboplatin and cisplatin in the D+CT arm, and 4.0 (1‒4) cycles of carboplatin and 4.0 (2‒4) cycles of cisplatin in the P+CT arm. All patients completed planned study treatment by the end of the study (Supplementary Table 1).

### Efficacy

#### Progression-free Survival

**Figure 1:**
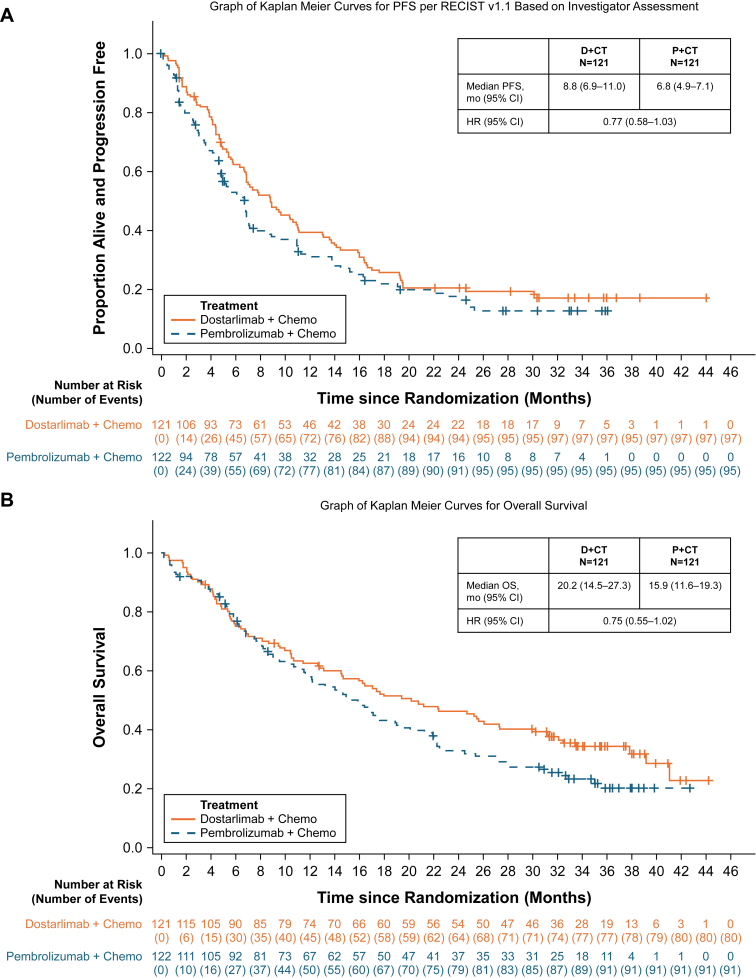
Kaplan–Meier (*A*) PFS and (*B*) OS curves of D+CT and P+CT. Plus (**+**) symbols represent individual censoring events. Chemo, chemotherapy; CI, confidence interval; D+CT, dostarlimab with chemotherapy; HR, hazard ratio; mo, months; OS, overall survival; P+CT, pembrolizumab with chemotherapy; PFS, progression-free survival.

As of LPLV, 97 (80%) and 95 (78%) patients experienced disease progression or death with D+CT and P+CT, respectively (79% maturity). The median follow-up time for PFS was 30.4 months (95% CI: 30.2–34.6) for D+CT and 30.4 months (95% CI: 24.7–33.6) for P+CT. The median PFS was 8.8 months (95% CI: 6.9–11.0) for D+CT and 6.8 months (95% CI: 4.9–7.1) for P+CT (HR: 0.77, 95% CI: 0.58–1.03) (Fig. 1*A*).

#### Overall Survival

As of LPLV, death was recorded in 80 (66%) and 91 (75%) patients treated with D+CT and P+CT, respectively (70% maturity). The median follow-up time for OS was 35.5 months (95% CI: 34.0–38.0) for D+CT and 35.2 months (95% CI: 32.9–37.8) for P+CT. The median OS was 20.2 months (95% CI: 14.5–27.3) for D+CT and 15.9 months (95% CI: 11.6–19.3) for P+CT (HR: 0.75, 95% CI: 0.55–1.02) (Fig. 1*B*). The OS by PD-L1 TPS status is presented in Supplementary Figure 1; however, because of small sample sizes and crossing of the curves, results should be interpreted with caution.

### Safety

Overall, the safety profiles in D+CT and P+CT arms were similar and consistent with previous analyses (Table 2).^4^ Treatment-emergent adverse events (TEAEs) led to treatment discontinuation in 29% and 38% of patients in D+CT and P+CT arms, respectively, and fatal treatment-related AEs occurred in 2% and 4% of patients in each arm. Investigator-determined fatal treatment-related AEs related to dostarlimab/pembrolizumab occurred in 2% for each arm and included urosepsis, immune-mediated lung disease, and pneumonitis for D+CT (<1% each), and respiratory failure and myelosuppression for P+CT (<1% each). Treatment-related TEAEs occurred in 85% and 81% of patients in D+CT and P+CT arms, respectively; the most frequent treatment-related TEAE in either treatment arm was anemia (43% and 39%, respectively). Overall, 32% and 39% of patients reported immune-related AEs for D+CT and P+CT, respectively, the most common of which were increased alanine aminotransferase (7%) and aspartate aminotransferase (7%) for D+CT, and increased alanine aminotransferase (7%) and pneumonitis (7%) for P+CT.

**Table 2:** Overall Summary of AEs

AE, n (%)	D+CT (N = 121)	P+CT (N = 122)
**AEs**	119 (98)	119 (98)
**Treatment-related TEAEs**	103 (85)	99 (81)
**Dostarlimab or pembrolizumab-related TRAEs**	86 (71)	70 (57)
**Grade ≥3 AEs**	79 (65)	80 (66)
**Grade ≥3 Treatment-related TEAEs**	49 (40)	52 (43)
**TEAEs leading to treatment discontinuation**	35 (29)	46 (38)
**TEAE leading to dostarlimab or pembrolizumab discontinuation**^a^	21 (17)	29 (24)
Pneumonitis	1 (<1)	7 (6)
Pneumonia	2 (2)	4 (3)
*Pneumocystis jirovecii* pneumonia	1 (<1)	1 (<1)
Septic shock	1 (<1)	1 (<1)
Alanine aminotransferase increased	2 (2)	3 (2)
Diarrhea	2 (2)	1 (<1)
Renal failure	1 (<1)	1 (<1)
**SAEs**	51 (42)	61 (50)
**Dostarlimab or pembrolizumab-related SAEs**	15 (12)	17 (14)
**Fatal TRAE**^b^	3 (2)	5 (4)
Immune-mediated lung disease	1 (<1)	0
Myelosuppression	0	1 (<1)
Pneumonia	0	1 (<1)
Pneumonitis	1 (<1)	0
Respiratory failure	0	1 (<1)
Septic shock	0	1 (<1)
Thrombocytopenia	0	1 (<1)
Urosepsis	1 (<1)	0
**Fatal dostarlimab or pembrolizumab-related TRAEs**	3 (2)	2 (2)
Urosepsis	1 (<1)	0
Immune-mediated lung disease	1 (<1)	0
Pneumonitis	1 (<1)	0
Respiratory failure	0	1 (<1)
Myelosuppression	0	1 (<1)
**irAEs**^c^	39 (32)	47 (39)
**irSAEs**	12 (10)	11 (9)

AE, adverse event; CT, chemotherapy; D+CT, dostarlimab with chemotherapy; ir, immune-related; P+CT, pembrolizumab with chemotherapy; SAE, serious adverse event; TEAE, treatment-emergent adverse event; TRAE, treatment-related adverse event.

a AEs below include those that occurred in more than 1 patient in both treatment arms.

b AEs described as treatment-related could be related to any study treatment agent.

c No new immune-related deaths were observed, and results are consistent with previous analyses.

## Discussion

This long-term analysis from PERLA provides a mature data set with a median follow-up of 35 months for OS, reaffirming the previous observations that efficacy and safety data were generally comparable between patients with metastatic nonsquamous NSCLC treated with D+CT or P+CT.

The PFS of 8.8 months for D+CT and 6.8 months for P+CT suggest a numerical trend favoring D+CT versus P+CT. The D+CT median PFS of 8.8 months is comparable to that observed in patients treated with pembrolizumab plus chemotherapy in KEYNOTE-189 (9.0 mo),^8^ although the PFS in PERLA was investigator-assessed, whereas PFS in KEYNOTE-189 was assessed by BICR. The lower median PFS for P+CT in this analysis versus the comparable arm in KEYNOTE-189 is likely owing to differences in trial populations (e.g., more patients had a PD-L1 TPS ≥50% in the pembrolizumab plus chemotherapy arm in KEYNOTE-189 versus the comparable arm in PERLA [32% versus 22%, respectively]), and differences in geographic location.^4^^,^^8^ The median PFS for P+CT in PERLA, however, is similar to that observed in real-world settings^9^; comparisons between PERLA, KEYNOTE-189, and real-world evidence have been discussed by Lim et al.^4^ Similarly, a numerical trend favoring D+CT versus P+CT was also suggested in the median OS of 20.2 months in D+CT versus 15.9 months in P+CT. Compared with the comparable arm in KEYNOTE-189, a lower median OS for P+CT was also observed in this analysis (22.0 versus 15.9 months).^8^ Other trials, such as EMPOWER-Lung 3, which evaluated the efficacy of cemiplimab^10^ plus chemotherapy in patients with stage III or IV NSCLC, reported a median OS of 21.1 months at the 2-year follow-up.^11^ IMpower130, a trial evaluating atezolizumab^12^ in combination with nanoparticle albumin-bound–paclitaxel reported a median OS of 18.6 months^13^; however, atezolizumab is an anti–PD-L1 therapy, and differences between drug classes have not been established.

Pharmacologic comparisons of dostarlimab and pembrolizumab have revealed that dostarlimab has a distinct epitope, paratope, and binding mode, and a greater steric clash with PD-L1 binding to PD-1 compared with pembrolizumab.^14^ Longitudinal tumor response using tumor size–overall survival modeling revealed a trend toward a numerically deeper and longer response for D+CT versus P+CT; however, the full clinical impact of dostarlimab’s distinct binding mode requires further investigation. This study has several strengths and limitations that have been previously reported by Lim et al.^4^

In conclusion, D+CT continues to exhibit strong clinical efficacy, similar to P+CT, in line with other first-line PD-1 inhibitor–chemotherapy combinations. D+CT and P+CT exhibit durable OS in first-line metastatic nonsquamous NSCLC. In addition, safety profiles remained consistent and similar to published data in PD-(L)1 inhibitors.^7^^,^^11^^,^^13^ These results support the ongoing investigation of dostarlimab as a combination partner for standard of care and novel therapies in solid tumors. Current clinical trials include the phase II GALAXIES study evaluating dostarlimab combinations in locally advanced or metastatic NSCLC,^15^ and JADE, a phase III study evaluating dostarlimab versus chemoradiation in patients with locally advanced unresected head and neck squamous cell carcinoma.^16^

## CRediT Authorship Contribution Statement

**Sun Min Lim:** Investigation, Writing - original draft, Writing - review & editing.

**Ana Laura Ortega Granados:** Investigation, Writing - original draft, Writing - review & editing.

**Gustavo dix Junqueira Pinto:** Investigation, Writing - original draft, Writing - review & editing.

**Christian Sebastián Fuentes:** Investigation, Writing - original draft, Writing - review & editing.

**Giuseppe Lo Russo:** Investigation, Writing - original draft, Writing - review & editing.

**Michael Schenker:** Investigation, Writing - original draft, Writing - review & editing.

**Jin Seok Ahn:** Investigation, Writing - original draft, Writing - review & editing.

**Filippo de Marinis:** Investigation, Writing - original draft, Writing - review & editing.

**Kenneth LockeJr:** Data curation, Formal analysis, Investigation, Validation, Writing - original draft, Writing - review & editing.

**Zsolt Szijgyarto:** Data curation, Formal analysis, Investigation, Validation, Writing - original draft, Writing - review & editing.

**Elena Buss:** Data curation, Formal analysis, Investigation, Validation, Writing - original draft, Writing - review & editing.

**Neda Stjepanovic:** Data curation, Formal analysis, Investigation, Validation, Writing - original draft, Writing - review & editing.

**Ivan Diaz-Padilla:** Data curation, Formal analysis, Investigation, Validation, Writing - original draft, Writing - review & editing.

**Solange Peters:** Investigation, Formal analysis, Writing - original draft, Writing - review & editing.

## Disclosure

Dr. Lim has received research grants from Yuhan and Johnson and Johnson; received consulting fees from AstraZeneca, Boehringer Ingelheim, Lilly, Takeda, Guardant, J Ints Bio, Therapex, Ono BMS; and has been an investigator for clinical trials sponsored by AstraZeneca, BeiGene, Boehringer Ingelheim, GSK, Roche, Hengrui, BridgeBio Therapeutics, Oscotec, Daichii Sankyo, Amgen, Therapex, Yuhan, Johnson and Johnson, and Takeda. Dr. Ortega-Granados is an employee of the Servicio Andaluz de Salud and has had an advisory role for Roche, Bristol Myers Squibb, and Merck Sharp and Dohme. Dr. Pinto has been an invited speaker for AstraZeneca and Daiichi Sankyo. Dr. Fuentes has been an invited speaker for Fundacion Respirar. Dr. Lo Russo has received consulting fees from Regeneron, Lilly, Roche, Novartis, BMS, MSD, AstraZeneca, Takeda, Amgen, Sanofi, Johnson and Johnson, Merck, Pierre Fabre, Bayer, Beigene, Daiichi, GSK, and Pfizer; received honoraria from Roche, Novartis, BMS, MSD, AstraZeneca, Takeda, Amgen, and Sanofi; received travel grants from Roche, BMS, and MSD; had an advisory role for Roche, Novartis, BMS, MSD, AstraZeneca, and Sanofi; and has been an investigator for clinical trials sponsored by Roche, Novartis, BMS, MSD, AstraZeneca, GSK, Amgen, and Sanofi. Dr. Schenker has had contracts for clinical trial activities (institutional and personal as site Principal Investigator) with GSK, Merck Serono, BMS, MSD, Roche, Sanofi, Regeneron, Astellas, Amgen, Bayer, BeiGene, Clovis, Tesaro, Gilead, Bioven, Novartis, Pfizer, Eli Lilly, Pharma Mar, AbbVie, AstraZeneca, Mylan, and Daiichi Sankyo. Dr. Ahn has been an invited speaker for Boryung, LG Chemical, Nokwon Medical, Samyang, Lilly Korea, Kyowa Kirin, Amgen Korea, Yuhan, AstraZeneca Korea, Menarini Korea, Bayer Korea, Takeda Pharmaceutical, Novartis Korea, BC World, Pfizer, Roche Korea, and Boehringer Ingelheim; and had an advisory role for Immuneoncia, Daiichi Sankyo Korea, Pfizer, Yuhan, Pharmbio Korea, Roche, Therapex, and Guardant. Dr. de Marinis has had an advisory role for AstraZeneca, Roche, Novartis, Merck, BMS, and MSD. Dr. Locke Jr and Dr. Szijgyarto are employed by GSK. Ms. Buss, Dr. Stjepanovic, and Dr. Diaz-Padilla are employed by GSK and hold financial equities in GSK. Dr. Peters has an advisory role with AbbVie, AiCME, Amgen, Arcus, AstraZeneca, Bayer, Beigene, Biocartis, BioInvent, Blueprint Medicines, Boehringer Ingelheim, Bristol Myers Squibb, Clovis, Daiichi Sankyo, Debiopharm, ecancer, Eli Lilly, Elsevier, F-Star, Fishawack, Foundation Medicine, Genzyme, Gilead, GSK, Illumina, Imedex, IQVIA, Incyte, iTeos, Janssen, Medscape, Medtoday, Merck Sharp and Dohme, Merck Serono, Merrimack, Novartis, Novocure, Oncology Education, Pharma Mar, Phosplatin Therapeutics, PER, Peerview, Pfizer, PRIME, Regeneron, RMEI, Roche/Genentech, RTP, Sanofi, Seattle Genetics, Takeda, and Vaccibody; has been an invited speaker for AiCME, AstraZeneca, Boehringer Ingelheim, Bristol Myers Squibb, eCancer, Eli Lilly, Foundation Medicine, Illumina, Imedex, Medscape, Merck Sharp and Dohme, Mirati, Novartis, PER, Peerview, Pfizer, Prime, Roche/Genentech, RTP, Sanofi, and Takeda; and received research grants from Amgen, AstraZeneca, BeiGene, Bristol Myers Squibb, GSK, Merck Sharp and Dohme, and Roche/Genentech.

## Supplementary Data

Supplementary Figure 1

Supplemenatry Protocol
