# Supplementary material for: Long-term Survival Analysis From PERLA, A Phase II Randomized Trial of Dostarlimab With Chemotherapy Versus Pembrolizumab With Chemotherapy in Metastatic Nonsquamous NSCLC
Source: JTO Clin Res Rep. 2025 Sep 4;6(10):100900. doi: 10.1016/j.jtocrr.2025.100900 (PMC12509971; doi:10.1016/j.jtocrr.2025.100900)
Supplement: Supplementary Figure 1 and Supplementary Table 1 [file mmc1.docx]

**Supplementary Materials**

**
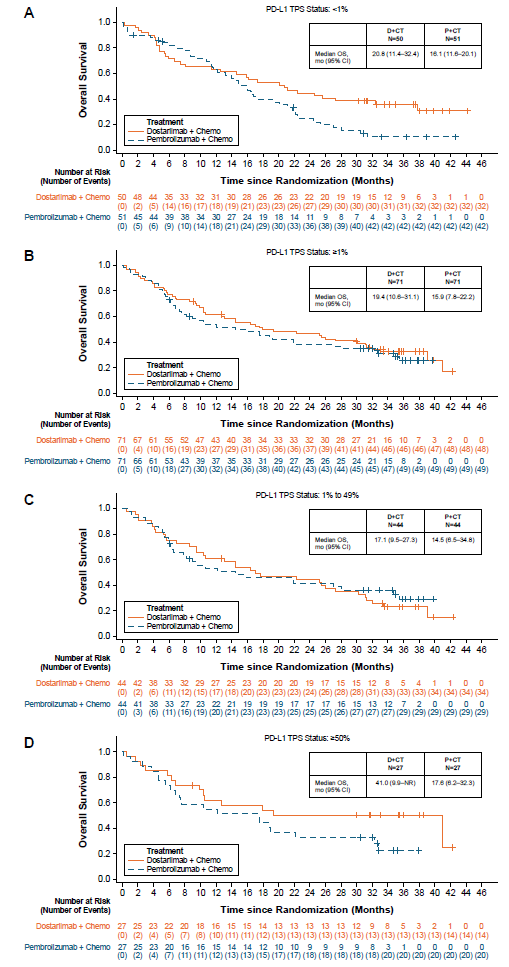
Figure S1. Kaplan–Meier OS curves by PD-L1 TPS status for D+CT and P+CT**

1. <1% PD-L1 TPS; B) ≥1% PD-L1 TPS; C) 1–49% PD-L1 TPS; and D) ≥50% PD-L1 TPS

Note: + symbols represent individual censoring events.

Chemo, chemotherapy; CI, confidence interval; D+CT, dostarlimab + chemotherapy; mo, months; OS, overall survival; P+CT, pembrolizumab + chemotherapy; PD-L1, programmed cell death – ligand 1; TPS, tumor proportion score.

**Supplemental Table 1: Treatment exposure for D+CT and P+CT**

| Variable | D+CT  (N=121) | P+CT  (N=122) |
| --- | --- | --- |
| Exposure to dostarlimab/pembrolizumab, n (%) | **121 (100)** | **122 (100)** |
| Median number of cycles, (range) | 13.0 (1–35) | 7.5 (1–35) |
| Median duration of exposure, months (range) | 9.0 (0.3–28.3) | 5.6 (0.2–27.7) |
| Exposure to pemetrexed, n (%) | **121 (100)** | **122 (100)** |
| Median number of cycles, (range) | 11.0 (1–35) | 7.0 (1–35) |
| Median duration of exposure, months (range) | 7.9 (0.3–25.7) | 5.5 (0.2–27.4) |
| Exposure to platinum chemotherapy (carboplatin), n (%) | **99 (81.8)** | **108 (88.5)** |
| Median number of cycles, (range) | 4.0 (1–4) | 4.0 (1–4) |
| Median duration of exposure, months (range) | 2.8 (0.3–4.4) | 2.8 (0.2–4.5) |
| Exposure to platinum chemotherapy (cisplatin), n (%) | **22 (18.2)** | **14 (11.5)** |
| Median number of cycles, (range) | 4.0 (1–4) | 4.0 (2–4) |
| Median duration of exposure, months (range) | 2.8 (0.7–3.9) | 2.8 (1.4–3.3) |

Duration of exposure = (min [date of last dose + 20, last contact date for discontinued subjects, date of death, data cut-off date] − date of first dose + 1)/30.4375.

D+CT, dostarlimab + chemotherapy; P+CT, pembrolizumab + chemotherapy.
