# Supplementary figures and images for: Long-term Survival Analysis From PERLA, A Phase II Randomized Trial of Dostarlimab With Chemotherapy Versus Pembrolizumab With Chemotherapy in Metastatic Nonsquamous NSCLC
Source: JTO Clin Res Rep. 2025 Sep 4;6(10):100900. doi: 10.1016/j.jtocrr.2025.100900 (PMC12509971; doi:10.1016/j.jtocrr.2025.100900)

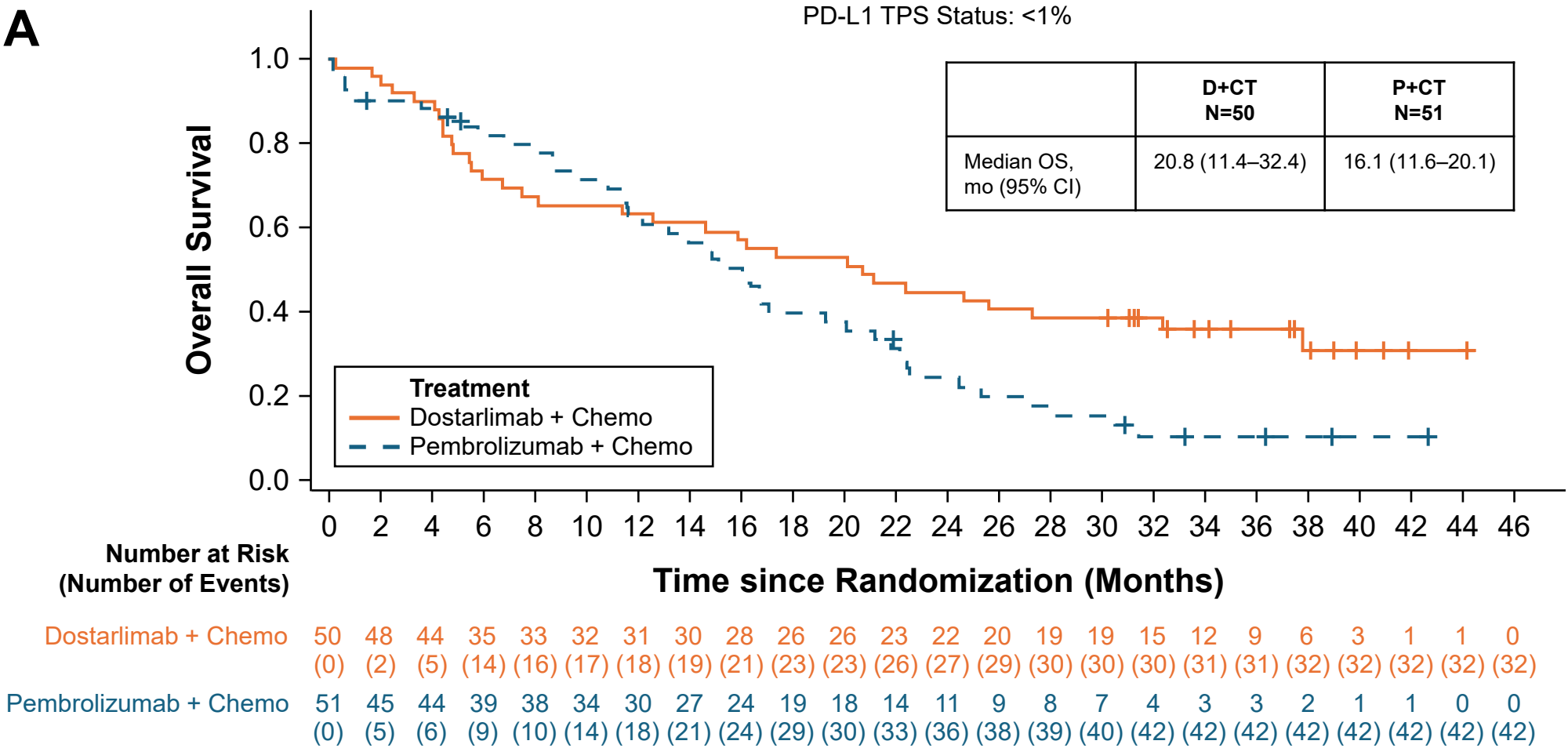

Supplement: Supplementary Figure 1 [file mmc2.pdf]
