# Supplementary material for: Long-term Survival Analysis From PERLA, A Phase II Randomized Trial of Dostarlimab With Chemotherapy Versus Pembrolizumab With Chemotherapy in Metastatic Nonsquamous NSCLC
Source: JTO Clin Res Rep. 2025 Sep 4;6(10):100900. doi: 10.1016/j.jtocrr.2025.100900 (PMC12509971; doi:10.1016/j.jtocrr.2025.100900)
Supplement: Supplemenatry Protocol [file mmc3.pdf]

A RANDOMIZED, PHASE 2, DOUBLE-BLIND STUDY TO EVALUATE THE EFFICACY OF DOSTARLIMAB PLUS CHEMOTHERAPY VERSUS PEMBROLIZUMAB PLUS CHEMOTHERAPY IN METASTATIC NON-SQUAMOUS NON-SMALL CELL LUNG CANCER

**Brief Title:**	Efficacy Comparison of Dostarlimab Plus Chemotherapy vs Pembrolizumab Plus Chemotherapy in Participants with Metastatic Non-squamous Non-small Cell Lung Cancer
**Protocol Number:**	213403 Amendment 5
**Compound Name:**	Dostarlimab (GSK4057190)
**Study Phase:**	Phase 2
**Sponsor Name and Legal Registered Address:**	GSK Research & Development Limited<br>980 Great West Road<br>Brentford<br>Middlesex, TW8 9GS<br>UK

SPONSOR SIGNATORY:

Neda Stjepanovic,
Medical Director

**Approval Date:**	14 February 2023
**Medical Monitor Name and Contact Information:**	Can be found in the Study Reference Manual.
**Regulatory Agency Identification Numbers:**	IND Number 150997<br>EudraCT Number 2020-002327-11
**Version of Protocol:**	05

Copyright 2023 the GSK group of companies. All rights reserved. Unauthorized copying or use of this information is prohibited.

PROTOCOL AMENDMENT SUMMARY OF CHANGES

Table 1: Document History

Document	Date	DNG Number	Sponsor
**Amendment 5 (Version 05)**	**14 February 2023**	**RPS-CLIN-050714**	**GSK**
**Amendment 4 (Version 04)**	30 November 2021	TMF-14193704	GSK
**Amendment 3 (Version 03)**	17 June 2021	TMF-12053714	GSK
**Amendment 2 (Version 02)**	10 September 2020	2020N447469_02	GSK
**Amendment 1 DEU-1 (Version 01 DEU-1)**	07 January 2021	2020N447469_01 DEU-1	GSK
**Amendment 1 (Version 01)**	24 July 2020	2020N447469_00	GSK
**Original Protocol (Version 00)**	18 June 2020	2020N436839_00	GSK

Abbreviations: DNG=document number generator; TMF=trial master file.

Amendment 5 (14 February 2023)

This amendment is considered to be non-substantial based on the criteria set forth in Article 10(a) of Directive 2001/20/EC of the European Parliament and the Council of the European Union because it neither significantly impacts the safety or physical/mental integrity of participants nor the scientific value of the study.

Overall Rationale for the Amendment 05

CCI [redacted]

Summary of Changes for the Amendment 05

Table 2: Summary of Changes for Amendment 05

Section(s) Affected	Description of Change	Brief Rationale
Section 8.3.3 Supplementary Analyses	Added 2 supplementary analyses at DCO2 and DCO3.	CCI [redacted]
Section 8.4.3 Primary Efficacy Analyses	Added supplementary analysis of ORR approximately15 months after the last subject was randomized.	
Appendix 4: Committees Structure	Clarified that blinded independent central review (BICR) will be utilized for review of radiological images and assessment of tumor response and progression.	
Appendix 4: Data Quality Assurance	Added involvement of central reading mechanism as content part of the Monitoring Plan.	
Appendix 4: Data Quality Assurance	Added a statement specifying the duration of documents storage (i.e., radiographic images) at external body.	
Appendix 4: Source Documents	Added a statement regarding documents which may be shared with external third parties contracted by GSK.	
Appendix 11: Amendment 4	Added protocol amendment 04 summary.	

French Coordinating Investigator Statement

Title of Protocol: “A Randomized, Phase 2, Double-blind study to evaluate the efficacy of Dostarlimab plus chemotherapy versus Pembrolizumab plus chemotherapy in metastatic non-squamous non-small cell lung cancer”.

Protocol Reference: **RPS-CLIN-050714**
Effective Date of Protocol Amendment n° 5: **14 February 2023**

Study Identifier: **213403 (PERLA)**

Compound Number: **GSK4057190 (Dostarlimab)**

I agree to act as the coordinating investigator for this clinical trial in France, according to the French law (n°2004-806 of 09 August 2004) “Article L1121-1 du Code de la Santé Publique”.

French Coordinating Investigator Name: **Pr Alain VERGNENEGRE**

Date: 20 MAR 2023

French Coordinating Investigator Signature:

PPD

TABLE OF CONTENTS

1. PROTOCOL SUMMARY ... 11
1.1. Synopsis ... 11
1.2. Schema ... 18
1.3. Schedule of Activities ... 18
2. INTRODUCTION ... 33
2.1. Study Rationale ... 33
2.2. Background ... 33
2.2.1. Lung Cancer ... 33
2.2.2. Programmed Cell Death, Immune Evasion, and Immunotherapy ... 34
2.2.3. Overview of PD-1 Inhibitors ... 35
2.2.4. PD-1 Inhibitors in Combination with Chemotherapy ... 36
2.2.5. Dostarlimab ... 38
2.2.6. Pembrolizumab ... 41
2.3. Benefit/Risk Assessment ... 41
2.3.1. Risk Assessment ... 42
2.3.2. Benefit Assessment ... 43
2.3.3. Overall Benefit/Risk Conclusion ... 43
3. OBJECTIVES AND ENDPOINTS ... 44
4. STUDY DESIGN ... 47
4.1. Overall Design ... 47
4.2. Scientific Rationale for Study Design ... 50
4.3. Justification for Dose ... 50
4.4. End of Study Definition ... 50
5. SELECTION OF STUDY POPULATION AND WITHDRAWAL CRITERIA ... 51
5.1. Inclusion Criteria ... 51
5.2. Exclusion Criteria ... 53
5.3. Lifestyle and/or Dietary Considerations ... 56
5.4. Screen Failures ... 56
5.5. Criteria for Temporarily Delaying ... 56
5.6. Withdrawal/Stopping Criteria ... 56
5.6.1. Liver Chemistry Stopping Criteria ... 58
5.6.2. QTc Stopping Criteria ... 59

5.7. Lost to Follow-up ... 59
5.8. Participant and Study Completion ... 60
6. STUDY TREATMENTS AND CONCOMITANT THERAPY ... 61
6.1. Study Treatments Administered ... 61
6.1.1. Dostarlimab ... 62
6.1.2. Pembrolizumab ... 62
6.1.3. Chemotherapy (Pemetrexed, Cisplatin, and Carboplatin) ... 62
6.1.4. Administration ... 63
6.2. Preparation/Handling/Storage/Accountability ... 63
6.3. Measures to Minimize Bias ... 64
6.3.1. Method of Treatment Assignment: Randomization ... 64
6.3.2. Blinding ... 64
6.4. Planned Dose Adjustments ... 65
6.4.1. Dose Levels and Dose Adjustment ... 65
6.4.2. Guidelines for Events of Special Interest ... 67
6.5. Participant-specific Dose Adjustments ... 78
6.6. Study Treatment Compliance ... 78
6.7. Treatment of Overdose ... 78
6.7.1. Protocol-defined Overdose ... 78
6.8. Treatment After the End of the Study ... 78
6.9. Concomitant Medications and Non-drug Therapies ... 79
6.9.1. Prohibited Medications and Non-drug Therapies ... 79
7. STUDY ASSESSMENTS AND PROCEDURES ... 80
7.1. General Guidelines ... 80
7.1.1. General Guidance for Treatment Continuity when Participants are Unable to Come into the Clinic ... 80
7.2. Screening and Critical Baseline Assessments ... 82
7.2.1. Baseline Assessments at Screening ... 82
7.2.2. Baseline Tumor Assessment (Documentation of Target and Non-target Lesions) ... 82
7.2.3. Determination of PD-L1 Status Prior to Randomization ... 83
7.3. Efficacy ... 84
7.3.1. Post-baseline Imaging and Tumor Assessment ... 84
7.4. Safety ... 86

7.4.1. Physical Examinations ... 86
7.4.2. ECOG Performance Status ... 86
7.4.3. Vital Signs ... 86
7.4.4. Pulse Oximetry ... 86
7.4.5. Electrocardiograms ... 86
7.4.6. Clinical Safety Laboratory Assessments ... 87
7.4.7. Pregnancy Testing ... 88
7.5. AEs, SAEs, and Other Safety Reporting ... 88
7.5.1. Time Period and Frequency for Collecting AE and SAE Information ... 88
7.5.2. Method of Detecting AEs and SAEs ... 89
7.5.3. Follow-up of AEs and SAEs ... 89
7.5.4. Regulatory Reporting Requirements for SAEs ... 90
7.5.5. Pregnancy ... 90
7.5.6. Cardiovascular and Death Events ... 91
7.5.7. Disease-related Events and/or Disease-related Outcomes Not Qualifying as SAEs ... 91
7.6. Translational Research (Biomarker and Pharmacodynamic Assessments) ... 91
7.6.1. Biomarker Sample Collection ... 91
7.6.2. Tumor Tissue Biomarker Analysis ... 92
7.6.3. Blood (Circulating) Biomarker Analysis ... 92
7.7. Pharmacokinetics ... 93
7.8. Immunogenicity ... 93
7.9. Genetics/Pharmacogenetics ... 94
7.10. Value Evidence and Outcomes Assessments ... 94
7.10.1. PROs ... 94
7.11. Biomedical Research ... 95
8. STATISTICAL CONSIDERATIONS AND DATA ANALYSES ... 97
8.1. Hypothesis ... 97
8.2. Sample Size Considerations ... 97
8.3. Data Analysis Considerations ... 97
8.3.1. Analysis Populations ... 97
8.3.2. Interim Analysis ... 98

8.3.3. Supplementary Analyses 98
8.4. Key Elements of Analysis Plan 98
8.4.1. Participant Disposition 99
8.4.2. Demographics, Medical History, Baseline Characteristics, and Concomitant Medications 99
8.4.3. Primary Efficacy Analyses 99
8.4.4. Secondary Efficacy Analyses 99
8.4.5. Exploratory Efficacy Analyses 100
8.4.6. Secondary Safety Analyses 100
8.4.7. Exploratory PK Analyses 101
8.4.8. Exploratory Immunogenicity Analyses 102
8.4.9. Exploratory Biomarker Analyses 102
8.4.10. Exploratory PRO Analyses 102
8.4.11. Other Analyses 102
9. REFERENCES 104

LIST OF TABLES

Table 1: Document History ... 2
Table 2: Summary of Changes for Amendment 04 ... 3
Table 3: Objectives and Endpoints for Study 213403 ... 12
Table 4: Schedule of Activities for Study 213403 ... 19
Table 5: Risk Assessment ... 42
Table 6: Objectives and Endpoints for Study 213403 ... 44
Table 7: Adequate Baseline Organ Function Inclusion Criteria ... 52
Table 8: Bundle Branch Block QTc Discontinuation Criteria ... 59
Table 9: Study Treatments for Study 213403 ... 61
Table 10: Dose Modifications for Chemotherapies Used in Study 213403 ... 66
Table 11: Recommended Dose Modifications for Hematological Chemotherapy Toxicities ... 66
Table 12: Recommended Dose Modifications for Non-hematological Chemotherapy Toxicities ... 67
Table 13: Dose Modification Guidelines for PD-1 Inhibitor-related AEs ... 68
Table 14: Infusion Reaction Treatment Guidelines ... 77
Table 15: Critical Data Collection and Safety Precautions ... 82
Table 16: Collecting and Reporting AEs, Pregnancy, and Survival ... 89
Table 17: Evaluation of Overall Response for Participants with Measurable Disease at Baseline ... 114
Table 18: Eastern Cooperative Oncology Group (ECOG) Performance Status Grading ... 117
Table 19: Protocol-required Safety Laboratory Tests ... 119
Table 20: Female Contraceptives Allowed During the Study ... 128
Table 21: Liver Chemistry Increased Monitoring Criteria ... 131
Table 22: Liver Chemistry Stopping Criteria ... 131
Table 23: Required Actions and Follow-up Assessments Following ANY Liver Stopping Event ... 133
Table 24: Abbreviations and Specialist Terms ... 145
Table 25: Summary of Changes for Amendment 1 ... 149
Table 26: Summary of Changes for Amendment 1 DEU-1 ... 150
Table 27: Summary of Changes from Amendment 1 DEU-1 (07 January 2021) to Amendment 3 (17 June 2021) ... 151
Table 28: Summary of Changes for Amendment 02 ... 157

Table 29: Summary of Changes for Amendment 03 158

LIST OF FIGURES

Figure 1: Schema for Study 213403 18
Figure 2: Liver Chemistry Stopping and Increased Monitoring Algorithm..... 58

1. PROTOCOL SUMMARY

1.1. Synopsis

Protocol Title

A Randomized, Phase 2, Double-blind Study to Evaluate the Efficacy of Dostarlimab Plus Chemotherapy versus Pembrolizumab Plus Chemotherapy in Metastatic Non-Squamous Non-Small Cell Lung Cancer

Brief Title

Efficacy Comparison of Dostarlimab Plus Chemotherapy vs Pembrolizumab Plus Chemotherapy in Participants with Metastatic Non-squamous Non-small Cell Lung Cancer

Rationale

The ongoing Phase 1/2 dose escalation and cohort expansion study (Study 4010-01-001 [GARNET; GSK Study 213346]), which is evaluating dostarlimab as monotherapy, has shown promising preliminary efficacy data in 67 participants with previously treated advanced non-small cell lung cancer (NSCLC); 18 out of 67 participants with NSCLC have achieved a confirmed partial response (PR) (26.9% objective response rate [ORR] by Response Evaluation Criteria in Solid Tumors [RECIST] v1.1), which is comparable to the reported efficacy of other programmed cell death protein 1 (PD-1) inhibitors in this setting. This response in participants with NSCLC in the GARNET study was observed even with negative enrichment by tumor proportion score (TPS).

Indirect comparison of the preliminary efficacy and safety profile of dostarlimab monotherapy from the GARNET study and the data of pembrolizumab monotherapy in second-line NSCLC (Herbst, Baas et al. 2016) indicates that the 2 PD-1 inhibitors may produce similar anti-tumor activity with comparable safety profiles. This study will provide a direct comparison of the efficacy, safety, and tolerability of dostarlimab versus pembrolizumab, both in combination with platinum-based chemotherapy.

The design of the proposed similarity study relies, in part, on the estimated treatment effect of pembrolizumab plus chemotherapy from 3 adequate and well-controlled studies (KEYNOTE studies) where said regimen was shown to be superior to chemotherapy alone (Langer, Gadgeel et al. 2016, Gandhi, Rodríguez-Abreu et al. 2018, Paz-Ares, Luft et al. 2018). The robustness of these studies in design and conduct help to provide a reliable estimate of pembrolizumab's effect. The proposed study follows KEYNOTE studies with respect to important study design features, including patient population and dosing of reference products.

Objectives and Endpoints

Table 3: Objectives and Endpoints for Study 213403

Objectives	Endpoints
**Primary**	
• To compare the ORR of PD-1 inhibitor dostarlimab vs pembrolizumab administered in combination with chemotherapy as evaluated using RECIST v1.1 based on BICR in participants with metastatic non-squamous NSCLC, without a known EGFR, ALK, ROS-1, or BRAF V600E mutation or other genomic aberration for which an approved targeted therapy is available, who have received no prior treatment of metastatic disease	• The primary efficacy endpoint ORR will be evaluated by RECIST v1.1 based on BICR and will be defined as the proportion of participants with BOR of CR or PR in the analysis population.
**Secondary**	
• To evaluate the following measures of clinical benefit of PD-1 inhibitor administered in combination with chemotherapy:<br>– OS<br>– PFS evaluated using RECIST v1.1 based on Investigator assessment	• OS will be defined as the time from the date of randomization to the date of death by any cause.<br>• PFS will be evaluated using RECIST v1.1 based on Investigator assessment and will be defined as the time from the date of randomization to the date of PD or death by any cause, whichever occurs first.
• To evaluate the safety of PD-1 inhibitor in combination with chemotherapy	• Assess the incidence of TEAEs, SAEs, irAEs, TEAEs leading to death, and AEs leading to discontinuation occurring while participants are on treatment or up to 90 days after the last dose of study treatment. Clinical laboratory parameters (hematology, chemistry, thyroid function, urinalysis), vital signs, ECOG performance status, ECG parameters, physical examinations, and usage of concomitant medications will be collected.

Table 3: Objectives and Endpoints for Study 213403 (Continued)

Objectives	Endpoints
CCI [redacted]	[redacted]

Table 3: Objectives and Endpoints for Study 213403 (Continued)

Objectives	Endpoints
CCI [redacted]	

Abbreviations: CCI [redacted]; ALK=anaplastic lymphoma kinase; BICR=blinded independent central review; CCI [redacted]; BRAF= proto-oncogene B-raf; CCI [redacted] CR=complete response; CCI [redacted]; ECG=electrocardiogram; ECOG=Eastern Cooperative Oncology Group; EGFR=epidermal growth factor receptor; CCI [redacted] EOT=End-of-Treatment; CCI [redacted] irAE=immune-related adverse event; CCI [redacted]; NSCLC=non-small cell lung cancer; ORR=objective response rate; OS=overall survival; PD=progressive disease; PD-1=programmed cell death protein 1; CCI [redacted]; PFS=progression-free survival; CCI [redacted] PR=partial response; CCI [redacted] RECIST=Response Evaluation Criteria in Solid Tumors; ROS-1=receptor tyrosine kinase-1; SAE=serious adverse event; TEAE=treatment-emergent adverse event; CCI [redacted]

Overall Design

This is a randomized, Phase 2, double-blind, 2-arm study to compare the efficacy and safety of PD-1 inhibitors dostarlimab and pembrolizumab, when administered in combination with chemotherapy, in male and female participants 18 years and older with non-squamous NSCLC without a known sensitizing epidermal growth factor receptor (EGFR), anaplastic lymphoma kinase (ALK), receptor tyrosine kinase-1 (ROS-1), or proto-oncogene B-raf (BRAF) V600E mutation or other genomic aberration for which an approved targeted therapy is available who have not received previous systemic anticancer therapy for metastatic disease.

The study consists of a Screening Period (Day-28 to Day-1) for completion of all Screening assessments and subsequent randomization, a Treatment Period, an End-of-Treatment (EOT) Visit (within 7 days of the decision to discontinue treatment for any reason), a Safety Follow-up Period with a visit at 30 (+7) and 90 (+7) days after the last dose of study treatment, and a Post-treatment Follow-up Period with assessments occurring 180 days after the last dose of study treatment and every 90 (±14) days thereafter, continuing until death, withdrawal of consent, or the end of study data collection. During the Treatment Period, clinic visits will occur every 3 weeks (Q3W). Any serious adverse events (SAEs) assessed as related to study participation should be reported from the signing of informed consent form, with all AEs and SAEs reported from the start of study treatment, and according to the time points specified in the schedule of activities (SoA) (Table 4) and Section 7.5.1.

To be eligible for the study, participants must have documented PD-L1 status by the 22C3 pharmDx assay (Agilent/Dako). If no prior PD-L1 result is available at the time of Screening, the participant can be tested locally using the aforementioned method, or central PD-L1 testing can be completed (refer to the Laboratory Manual for country-specific details of central assessment). For participants requiring PD-L1 testing at the time of study entry, an archival formalin-fixed paraffin-embedded (FFPE) tumor tissue specimen, which may have been collected at any time prior to Screening, can be submitted for analysis within 28 days of the participant's first dose. Specimens used to confirm diagnosis or collected after the participant has been diagnosed with metastatic disease will be preferred for determination of PD-L1 status. Both tissue block and freshly cut (within 6 months) slides (10 to 15) are acceptable. If no archival FFPE tumor tissue is available, a fresh tumor tissue biopsy should be obtained and tested within 28 days of the participant's first dose. Biopsies obtained prior to receipt of adjuvant/neoadjuvant chemotherapy will be permitted if a fresh biopsy is not feasible. As the results of baseline PD-L1 status assessment will be used as a stratification factor in this study, results must be provided prior to randomization. Participants who do not have documented PD-L1 status and cannot provide archived or fresh tissue sample for PD-L1 testing will not be eligible for the study.

Within 35 days prior to the first dose, participants must have a baseline tumor assessment of the chest and abdomen (including the entire liver and both adrenal glands). Other regions should be imaged as clinically indicated (e.g., pelvis, brain, etc). This assessment should be conducted by intravenous (IV) contrast-enhanced computed tomography (CT) or magnetic resonance imaging (MRI) scan. MRI should be used for abdominal (and pelvic) imaging if CT contrast is contraindicated, and, preferably, for IV

contrast-enhanced imaging of the brain. The same imaging technique should be used in a participant throughout the study. The CT component of positron emission tomography (PET)/CT may be used according to RECIST v1.1 guidelines, with full radiation dose diagnostic CT and IV CT contrast, and as clinically indicated. At each post-baseline assessment, evaluations of the sites of the disease identified by these scans are required.

Following informed consent and completion of all Screening assessments, all participants who meet the eligibility criteria will be randomized 1:1 to receive chemotherapy in combination with either dostarlimab or pembrolizumab. Randomization will be completed in a blinded manner using an interactive web response system. Dostarlimab and pembrolizumab must be dispensed in a blinded manner. Details of blinding will be documented in site-specific blinding plans. The dosing regimen is described in Section 6.1.4. Randomization will be stratified by the following 2 factors:

- PD-L1 status of the tumor (TPS <1% vs 1% to 49% vs ≥50%)
- Smoking status (never vs former/current)

To support continued tumor assessment while on study, serial imaging will then be performed at 6 weeks (42 [±7] days) and again at 12 weeks (84 [±7] days) from the randomization date. Following the 12-week scan, imaging will be performed every 9 weeks (Q9W) (every 63 [±7] days), or more frequently, if clinically indicated. After 48 weeks, participants who remain on treatment will have imaging performed every 12 weeks (Q12W) (every 84 [±7] days). Imaging should continue to be performed until discontinuation of study treatment due to disease progression with clinical instability, start of subsequent anticancer treatment, withdrawal of informed consent, or death, whichever comes first. Imaging must be performed on a calendar schedule and should not be affected by dose interruptions/delays. All radiographic images/scans at the specified time points, as well as any unscheduled images/scans, will be collected and stored centrally for blinded independent central review (BICR), potential future evaluation, and archiving. Digital copies of all Digital Imaging and Communications in Medicine (DICOM)-formatted scans must be maintained at the Investigative site.

Following the initial radiologic assessment of progressive disease (PD), clinically stable participants should continue study treatment and a follow-up scan should be performed at least 4 weeks and no longer than 8 weeks later. The follow-up scan will provide additional information to the Investigator for participant management and further treatment decisions. Further guidance for the assessment of scans acquired after RECIST v1.1-defined PD are provided in Appendix 1. For participants who are not clinically stable (clinically progressing) at the initial radiologic assessment of PD, study treatment will be discontinued and a follow-up scan is not required. Following the initial radiologic assessment of complete response (CR) or PR, a confirmatory follow-up scan should be performed at least 4 weeks later.

To support exploratory genomic and protein biomarker analysis participants are highly encouraged to submit an archival FFPE tumor tissue specimen, collected at the time of or after the diagnosis of metastatic disease from location(s) not irradiated prior to biopsy, at Screening. Both tissue block and freshly cut (within 6 months) slides are acceptable. If archival tissue is not available, the participant can elect to undergo biopsy prior to study entry. Open biopsies, punch biopsies, and core biopsies (3 samples) are acceptable.

Fine-needle aspirate, frozen sample, plastic embedded sample, cell block, clot, and cytological specimen are not acceptable for analysis. The archival tumor tissue sample should be submitted within 28 days of the participant's first dose of study treatment. Please note, if a participant has submitted an archival or fresh tumor tissue sample fitting these specifications as part of PD-L1 status determination, that sample may also be used for the exploratory biomarker analyses.

Blood samples to assess the pharmacokinetics (PK) of dostarlimab and pembrolizumab will be collected from all participants during treatment, at EOT, at the 90-day Safety Follow-up Visit, and at the 180-day Post-treatment Follow-up Visit. Immunogenicity with associated drug concentration will be analyzed in participants administered dostarlimab and, if needed, in participants administered pembrolizumab. Blood samples to assess circulating tumor biomarkers including ctDNA will be collected from all participants at Screening and on study.

Patient-reported outcomes (PROs) will be collected in alignment with study treatment administration at scheduled visits during the Treatment Period and at the EOT Visit.

Biomedical Research

CCI [redacted]

Brief Summary

The purpose of this study is to compare the efficacy and safety of dostarlimab in combination with chemotherapy to the efficacy and safety of pembrolizumab in combination with chemotherapy in participants with non-squamous NSCLC who have not received previous systemic anticancer therapy for metastatic disease. Study details include the following:

- Study duration: 5 years
- Treatment duration: Up to 35 cycles total

- Visit frequency: Q3W (cycles are 21 [±3] days)

Number of Participants

Approximately 240 participants will be randomly assigned to 2 study treatments, such that approximately 120 evaluable participants in each of the 2 arms complete the study.

Treatment Groups and Duration

Participants will be randomized in a 1:1 ratio into the dostarlimab plus chemotherapy arm or the pembrolizumab plus chemotherapy arm. Prior to randomization, Investigators will select, based on their clinical judgment, cisplatin or carboplatin as the chemotherapy to be administered. Randomization will be stratified by PD-L1 status of the tumor (TPS <1% versus 1% to 49% versus ≥50%) and smoking status (never vs former/current). A PD-L1 result obtained prior to Screening is allowed.

The study will last approximately 5 years.

Safety Data Monitoring

To safeguard the interest and safety of the participants in the study, a safety monitoring/review panel will be used to conduct review of incoming data to monitor emerging safety signals.

1.2. Schema

The schema for Study 213403 is presented in Figure 1.

Figure 1: Schema for Study 213403

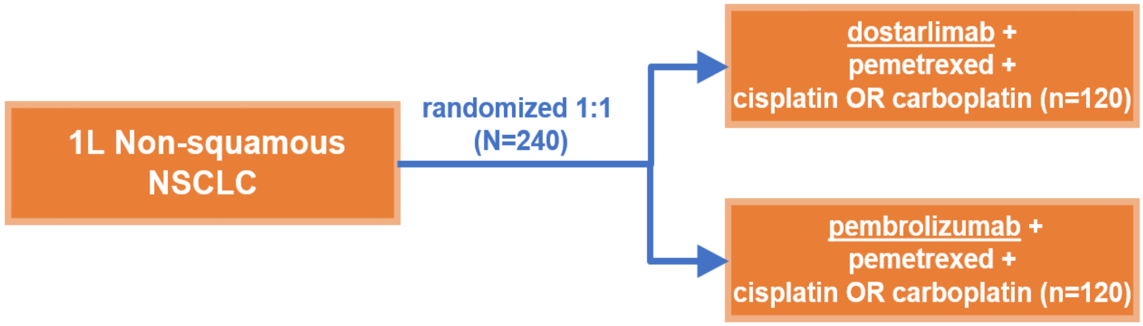

Abbreviations: 1L=first-line; NSCLC=non-small cell lung cancer.
Note: Both arms will be stratified by PD-L1 status (TPS <1% vs 1% to 49% vs ≥50%) and smoking status (never vs former/current).

1.3. Schedule of Activities

Schedules of activities for Study 213403 are provided in Table 4. Each treatment cycle is 3 weeks (21 [±3] days).

Table 4: Schedule of Activities for Study 213403

Visit/Cycle:	Screening	Treatment Period		EOT	Safety FUP	Post-treatment FUP	Notes
		C1	Subsequent Cycles				
**Day:**<br>**Procedure:**	**Up to 28 days before D1**	**D1**	**Cn, D1 (±3)**	**Within 7 days after last dose (or decision to discontinue)**	**30 (+7) and 90 (+7) days after last dose**	**180 days after last dose, then every 90 (±14) days**	Treatment cycles are 21 days long with dosing scheduled on D1 of each cycle.<br>All assessments should be done prior to drug administration and within 72 h prior to 1st dose of study drugs administrated in that cycle, unless otherwise specified. Visits starting at Cycle 2 can be performed ±3 days of the scheduled date.[1]
Informed consent	X						
Inclusion/exclusion criteria review	X						Clinical status should be rechecked before randomization and/or first dose of study treatment.
Demographics	X						
Medical, surgical, cancer, smoking, and medication history	X						
Current medical conditions and medications	X						

Table 4: Schedule of Activities for Study 213403 (Continued)

Visit/Cycle:	Screening	Treatment Period		EOT	Safety FUP	Post-treatment FUP	Notes
		C1	Subsequent Cycles				
**Day:**<br>**Procedure:**	**Up to 28 days before D1**	**D1**	**Cn, D1 (±3)**	**Within 7 days after last dose (or decision to discontinue)**	**30 (+7) and 90 (+7) days after last dose**	**180 days after last dose, then every 90 (±14) days**	Treatment cycles are 21 days long with dosing scheduled on D1 of each cycle.<br>All assessments should be done prior to drug administration and within 72 h prior to 1st dose of study drugs administrated in that cycle, unless otherwise specified. Visits starting at Cycle 2 can be performed ±3 days of the scheduled date.[1]
Tumor assessment	The baseline tumor assessment will be completed within 35 days prior to the first dose of study treatment. To support continued tumor assessment while on study, serial imaging will then be performed at 6 weeks (42 [±7] days) and again at 12 weeks (84 [±7] days) from the randomization date. Following the 12 week scan, imaging will be performed Q9W (every 63 [±7] days), or more frequently, if clinically indicated. After 48 weeks, participants who remain on treatment will have imaging performed Q12W (every 84 [±7] days). Imaging should continue to be performed until discontinuation of study treatment due to disease progression with clinical instability, start of subsequent anticancer treatment, withdrawal of informed consent, or death, whichever comes first. Imaging must be performed on a calendar schedule and should not be affected by dose interruptions/delays. All radiographic images/scans at the specified time points, as well as any unscheduled images/scans, will be collected and stored centrally for BICR, potential future evaluation, and archiving. Digital copies of all DICOM formatted scans must be maintained at the Investigative site.						If a participant had a tumor assessment of the clinically indicated areas within the 35 days before dosing on C1/D1 but prior to signing the main ICF, an additional CT/MRI scan for study screening is not required. CT/MRI scans completed during Screening prior to signing the main ICF must have been performed and available for submission per the image acquisition guidelines.<br>If EOT occurs between the scheduled radiologic disease assessments, the scans do not need to be repeated at EOT if fewer than 5 weeks have passed since the time of the previous disease assessment that did not document PD.<br>For more details on tumor assessment, see Section 7.2.2, Section 7.3.1, and Appendix 1 (RECIST).

Table 4: Schedule of Activities for Study 213403 (Continued)

Visit/Cycle:	Screening	Treatment Period		EOT	Safety FUP	Post-treatment FUP	Notes
		C1	Subsequent Cycles				
**Day:**<br>**Procedure:**	**Up to 28 days before D1**	**D1**	**Cn, D1 (±3)**	**Within 7 days after last dose (or decision to discontinue)**	**30 (+7) and 90 (+7) days after last dose**	**180 days after last dose, then every 90 (±14) days**	Treatment cycles are 21 days long with dosing scheduled on D1 of each cycle.<br>All assessments should be done prior to drug administration and within 72 h prior to 1st dose of study drugs administrated in that cycle, unless otherwise specified. Visits starting at Cycle 2 can be performed ±3 days of the scheduled date.[1]
PD-L1 status	X						Required for randomization; may require tumor tissue submission or collection (see Section 7.2.3.).
Archival (or fresh) tumor tissue sample	X						Sample is required, if available. If a participant has submitted an archival or fresh tumor tissue sample as part of PD-L1 status determination, that sample may also be used for the exploratory biomarker analyses; see Section 7.6.1. If PD-L1 status is known and the participant has agreed to provide archival sample via the optional consent, this sample may be provided within 28 days of the start of study treatment.

Table 4: Schedule of Activities for Study 213403 (Continued)

Visit/Cycle:	Screening	Treatment Period		EOT	Safety FUP	Post-treatment FUP	Notes
		C1	Subsequent Cycles				
**Day:**<br>**Procedure:**	**Up to 28 days before D1**	**D1**	**Cn, D1 (±3)**	**Within 7 days after last dose (or decision to discontinue)**	**30 (+7) and 90 (+7) days after last dose**	**180 days after last dose, then every 90 (±14) days**	Treatment cycles are 21 days long with dosing scheduled on D1 of each cycle.<br>All assessments should be done prior to drug administration and within 72 h prior to 1st dose of study drugs administrated in that cycle, unless otherwise specified. Visits starting at Cycle 2 can be performed ±3 days of the scheduled date.[1]
Evaluate genomic aberration	X						Participant is required to have histologically or cytologically confirmed metastatic non squamous NSCLC with documented absence of a sensitizing EGFR, ALK, ROS 1, or BRAF V600E mutation or other genomic aberration for which an approved targeted therapy is available (Inclusion Criteria 2, Section 5.1). An assessment at Screening is only required if prior documentation is not available.
Optional blood sample for genetic testing	X						Sample may be obtained after randomization during first cycle if not collected at screening.
Randomization	X						Randomization could be completed any time during the Screening Period once eligibility criteria have been confirmed.

Table 4: Schedule of Activities for Study 213403 (Continued)

**Visit/Cycle:**	**Screening**	**Treatment Period**		**EOT**	**Safety FUP**	**Post-treatment FUP**	**Notes**
		**C1**	**Subsequent Cycles**				
**Day:**<br>**Procedure:**	**Up to 28 days before D1**	**D1**	**Cn, D1 (±3)**	**Within 7 days after last dose (or decision to discontinue)**	**30 (+7) and 90 (+7) days after last dose**	**180 days after last dose, then every 90 (±14) days**	Treatment cycles are 21 days long with dosing scheduled on D1 of each cycle.<br>All assessments should be done prior to drug administration and within 72 h prior to 1st dose of study drugs administrated in that cycle, unless otherwise specified. Visits starting at Cycle 2 can be performed ±3 days of the scheduled date.[1]
PROs: EORTC-QLQ-C30, EORTC-QLQ-LC13, PRO-CTCAE, FACT-GP5, PGIS, and PGIC		Whenever possible and within capability, PROs should be completed the day before the visit, at the participant's home. PROs should be collected prior to any clinical procedures, whenever possible. Collect PROs on C1D1, then every cycle until C4D1, every 3 cycles from Cycle 7 until Cycle 16, and every 4 cycles thereafter until EOT, at the EOT Visit, and at the 30-day Safety FUP Visit.					Completed prior to examinations and discussion of results with site staff.<br>PGIC is not collected at C1D1.
ECOG performance status	X	X	X	X	X		See Appendix 2.
CBC	X	X	X	X	X		If Screening assessments were performed within 72 hours of D1, repeat testing is not required.<br>See Appendix 3 for analytes.
Serum chemistry	X	X	X	X	X		
Urinalysis	X		Every 6 cycles starting at C6D1				See Appendix 3 for analytes.
Thyroid panel	X		Q6W starting at C2D1	X	X		Collect sample at 90-day Safety FUP Visit only if assessment is clinically indicated.

Table 4: Schedule of Activities for Study 213403 (Continued)

Visit/Cycle:	Screening	Treatment Period		EOT	Safety FUP	Post-treatment FUP	Notes
		C1	Subsequent Cycles				
**Day:**<br>**Procedure:**	**Up to 28 days before D1**	**D1**	**Cn, D1 (±3)**	**Within 7 days after last dose (or decision to discontinue)**	**30 (+7) and 90 (+7) days after last dose**	**180 days after last dose, then every 90 (±14) days**	Treatment cycles are 21 days long with dosing scheduled on D1 of each cycle.<br>All assessments should be done prior to drug administration and within 72 h prior to 1st dose of study drugs administrated in that cycle, unless otherwise specified. Visits starting at Cycle 2 can be performed ±3 days of the scheduled date.[1]
Highly sensitive urine or serum pregnancy test (WOCBP only)	X	X	X	Monthly for at least 180 days after the last dose of study treatment (Note: duration of contraceptive use after last dose of chemotherapy must be consistent with local requirements and local approved product labels; however, the minimum duration is 180 days after last dose of chemotherapy)			A highly sensitive pregnancy test (urine or serum, as required by local regulations) is required within 72 hours before study treatment administration. At Screening, if a urine test cannot be confirmed as negative (e.g., an ambiguous result), a serum pregnancy test is required. In such cases, the participant must be excluded from participation if the serum pregnancy result is positive. See Section 5.1, Section 7.4.7, and Appendix 3.
HIV, hepatitis B, and hepatitis C screening	X						If a participant was tested within 3 months prior to the first dose of study treatment, testing at Screening is not required. See Appendix 3.
Full physical examination, including height	X			X			Height will be measured at Screening only.

**Visit/Cycle:**	**Screening**	**Treatment Period**		**EOT**	**Safety FUP**	**Post-treatment FUP**	**Notes**
		**C1**	**Subsequent Cycles**				
**Day:**<br>**Procedure:**	**Up to 28 days before D1**	**D1**	**Cn, D1 (±3)**	**Within 7 days after last dose (or decision to discontinue)**	**30 (+7) and 90 (+7) days after last dose**	**180 days after last dose, then every 90 (±14) days**	Treatment cycles are 21 days long with dosing scheduled on D1 of each cycle.<br>All assessments should be done prior to drug administration and within 72 h prior to 1st dose of study drugs administrated in that cycle, unless otherwise specified. Visits starting at Cycle 2 can be performed ±3 days of the scheduled date.[1]
Vital signs and weight	X	X	X	X	X		

Table 4: Schedule of Activities for Study 213403 (Continued)

Visit/Cycle:	Screening	Treatment Period		EOT	Safety FUP	Post-treatment FUP	Notes
		C1	Subsequent Cycles				
**Day:**<br>**Procedure:**	**Up to 28 days before D1**	**D1**	**Cn, D1 (±3)**	**Within 7 days after last dose (or decision to discontinue)**	**30 (+7) and 90 (+7) days after last dose**	**180 days after last dose, then every 90 (±14) days**	Treatment cycles are 21 days long with dosing scheduled on D1 of each cycle.<br>All assessments should be done prior to drug administration and within 72 h prior to 1st dose of study drugs administrated in that cycle, unless otherwise specified. Visits starting at Cycle 2 can be performed ±3 days of the scheduled date.[1]
Pulse oximetry	X	X	X	X	X*		*At the 30-day Safety Follow-up Visit, only<br>Performed using local standard procedures; to be completed prior to dosing when performed during the Treatment Period.
12-lead ECG	X	X		X			
AE monitoring	Collection of AEs begins after first treatment. AEs are required to be collected from the first treatment through 30 days after cessation of study treatment. However, SAEs are required to be captured through 90 days after cessation of study treatment (or to a minimum of 30 days post-treatment if the participant starts alternate anticancer therapy), and any pregnancies that occur while participant is on the study are to be captured. SAEs assessed as related to study participation and AESIs of any causality should be collected from the time informed consent was signed until study closeout. Monitoring of AEs after 90 days may be done via telephone assessment (Section 7.5.1).						

Visit/Cycle:	Screening	Treatment Period		EOT	Safety FUP	Post-treatment FUP	Notes
		C1	Subsequent Cycles				
**Day:**<br>**Procedure:**	**Up to 28 days before D1**	**D1**	**Cn, D1 (±3)**	**Within 7 days after last dose (or decision to discontinue)**	**30 (+7) and 90 (+7) days after last dose**	**180 days after last dose, then every 90 (±14) days**	Treatment cycles are 21 days long with dosing scheduled on D1 of each cycle.<br>All assessments should be done prior to drug administration and within 72 h prior to 1st dose of study drugs administrated in that cycle, unless otherwise specified. Visits starting at Cycle 2 can be performed ±3 days of the scheduled date.[1]
Blood sample for ctDNA biomarker analysis	X		C5D1 only				

Table 4: Schedule of Activities for Study 213403 (Continued)

Visit/Cycle:	Screening	Treatment Period		EOT	Safety FUP	Post-treatment FUP	Notes
		C1	Subsequent Cycles				
**Day:**<br>**Procedure:**	**Up to 28 days before D1**	**D1**	**Cn, D1 (±3)**	**Within 7 days after last dose (or decision to discontinue)**	**30 (+7) and 90 (+7) days after last dose**	**180 days after last dose, then every 90 (±14) days**	Treatment cycles are 21 days long with dosing scheduled on D1 of each cycle.<br>All assessments should be done prior to drug administration and within 72 h prior to 1st dose of study drugs administrated in that cycle, unless otherwise specified. Visits starting at Cycle 2 can be performed ±3 days of the scheduled date.[1]
Blood sample for PDy biomarker analysis	X	Predose and end of infusion	Predose for C2 and predose and end of infusion for C5, only				Predose is within -2 to 0 hours (not including 0 hours).<br>End of infusion is within >0 to +1 hours after immunotherapy infusion.
Predose blood sample for PK/immunogenicity		X	C2, C5, C11, and every 6 cycles thereafter (up to 35 cycles total)				Predose is within -2 to 0 hours (not including 0 hours).
Immunotherapy administered		X	X				Immunotherapy (500-mg 30- [-5 to +15] minute IV infusion of dostarlimab [Arm 1] or 200-mg 30- [-5 to +15] minute IV infusion of pembrolizumab [Arm 2]) will be administered for up to 35 cycles total (approximately 24 months).

Table 4: Schedule of Activities for Study 213403 (Continued)

Visit/Cycle:	Screening	Treatment Period		EOT	Safety FUP	Post-treatment FUP	Notes
		C1	Subsequent Cycles				
**Day:**<br>**Procedure:**	**Up to 28 days before D1**	**D1**	**Cn, D1 (±3)**	**Within 7 days after last dose (or decision to discontinue)**	**30 (+7) and 90 (+7) days after last dose**	**180 days after last dose, then every 90 (±14) days**	Treatment cycles are 21 days long with dosing scheduled on D1 of each cycle.<br>All assessments should be done prior to drug administration and within 72 h prior to 1st dose of study drugs administrated in that cycle, unless otherwise specified. Visits starting at Cycle 2 can be performed ±3 days of the scheduled date[1]
Pemetrexed treatment administered		X	X				500 mg/m^2 pemetrexed will be administered via 10-minute IV infusion, following immunotherapy administration. Pre-treatment for pemetrexed (folic acid, vitamin B12, and glucocorticoids) must be administered according to the product label and local guidelines.

**Visit/Cycle:**	**Screening**	**Treatment Period**		**EOT**	**Safety FUP**	**Post-treatment FUP**	**Notes**
		**C1**	**Subsequent Cycles**				
**Day:**<br>**Procedure:**	**Up to 28 days before D1**	**D1**	**Cn, D1 (±3)**	**Within 7 days after last dose (or decision to discontinue)**	**30 (+7) and 90 (+7) days after last dose**	**180 days after last dose, then every 90 (±14) days**	Treatment cycles are 21 days long with dosing scheduled on D1 of each cycle.<br>All assessments should be done prior to drug administration and within 72 h prior to 1st dose of study drugs administrated in that cycle, unless otherwise specified. Visits starting at Cycle 2 can be performed ±3 days of the scheduled date[1]
Platinum chemotherapy treatment administered		X	X				Platinum chemotherapy will be administered for the first 4 cycles only, following pemetrexed administration. If selected by the Investigator, cisplatin (75 mg/m^2) will be given via IV infusion (approximately 30 minutes after pemetrexed infusion). **If selected by the Investigator,** carboplatin (AUC 5 mg/mL/min via IV infusion) immediately following the pemetrexed infusion. Chemotherapeutic agents should be administered according to local practice and local approved product labels. For participants receiving cisplatin, pre- and post-treatment hydration procedures must be administered according to local practice and labels.

Table 4: Schedule of Activities for Study 213403 (Continued)

Visit/Cycle:	Screening	Treatment Period		EOT	Safety FUP	Post-treatment FUP	Notes
		C1	Subsequent Cycles				
**Day:**<br>**Procedure:**	**Up to 28 days before D1**	**D1**	**Cn, D1 (±3)**	**Within 7 days after last dose (or decision to discontinue)**	**30 (+7) and 90 (+7) days after last dose**	**180 days after last dose, then every 90 (±14) days**	Treatment cycles are 21 days long with dosing scheduled on D1 of each cycle.<br>All assessments should be done prior to drug administration and within 72 h prior to 1st dose of study drugs administrated in that cycle, unless otherwise specified. Visits starting at Cycle 2 can be performed ±3 days of the scheduled date[1]
End of infusion blood sample for PK		X	C2 and C5				End of immunotherapy infusion (>0 to +1 hour) samples will be collected for PK only on D1 of C1, C2, and C5.
Additional blood sample for PK/immunogenicity				X	X	X	Only 2 FUP samples will be collected: 1 at the 90-day Safety FUP Visit and 1 at the 180-day FUP Visit.
Concomitant medications/procedures		Recorded from the first dose of study treatment through Safety FUP					Any subsequent anticancer treatment started during the study should also be collected.
Symptom-directed physical examination		X	X		X		

Table 4: Schedule of Activities for Study 213403 (Continued)

Visit/Cycle:	Screening	Treatment Period		EOT	Safety FUP	Post-treatment FUP	Notes
		C1	Subsequent Cycles				
**Day:**<br>**Procedure:**	**Up to 28 days before D1**	**D1**	**Cn, D1 (±3)**	**Within 7 days after last dose (or decision to discontinue)**	**30 (+7) and 90 (+7) days after last dose**	**180 days after last dose, then every 90 (±14) days**	Treatment cycles are 21 days long with dosing scheduled on D1 of each cycle.<br>All assessments should be done prior to drug administration and within 72 h prior to 1st dose of study drugs administrated in that cycle, unless otherwise specified. Visits starting at Cycle 2 can be performed ±3 days of the scheduled date[1]
Survival, AESIs, and study-drug related SAEs						X	Participant will be followed until study closeout for survival status, AESIs (regardless of causality), and study-drug related SAEs.

Abbreviations: AE=adverse event; AESI=AE of special interest, AUC=area under the concentration-time curve; BICR=blinded independent central review; C=Cycle; Cn=Cycle subsequent to Cycle 1, i.e., C2, C3, C4, etc; CBC=complete blood count; CT=computed tomography; ctDNA=circulating tumor deoxyribonucleic acid; D=day; DICOM=Digital Imaging and Communications in Medicine; ECG=electrocardiogram; ECOG=Eastern Cooperative Oncology Group; EORTC-QLQ-C30=European Organisation for Research and Treatment of Cancer Quality of Life Questionnaire 30-item Core Module; EORTC-QLQ-LC13=European Organisation for Research and Treatment of Cancer Quality of Life Questionnaire 13-item Lung Cancer Module; EOT=End-of-Treatment; FACT-GP5=Functional Assessment of Cancer Therapy-General Population; FFPE=formalin-fixed paraffin-embedded; FUP=follow-up; ICF=informed consent form; IV=intravenous; MRI=magnetic resonance imaging; n=number of subsequent; PD-L1=programmed death-ligand 1; PDy=pharmacodynamic; PGIC=Patient Global Impression of Change; PGIS=Patient Global Impression of Severity; PK=pharmacokinetics; PRO=patient-reported outcome; PRO-CTCAE=Patient-reported Outcomes Version of the Common Terminology Criteria for Adverse Events; Q12W=every 12 weeks; Q6W=every 6 weeks; Q9W=every 9 weeks; RECIST=Response Evaluation Criteria in Solid Tumors; SAE=serious adverse event; WOCBP=woman of childbearing potential.

.[1] Standard-of-care tests/procedures, including laboratory assessments, ECG, physical examination, vital signs, height, weight, and ECOG performance status performed before the participant signs the ICF can be used as part of the Screening assessments, as long as the tests/procedures are performed within the 28-day Screening Period.

2. INTRODUCTION

2.1. Study Rationale

The ongoing Phase 1/2 dose escalation and cohort expansion study (Study 4010-01-001 [GARNET; GSK Study 213346]), which is evaluating dostarlimab as monotherapy, has shown promising preliminary efficacy data in 67 participants with previously treated advanced NSCLC; 18 out of 67 participants with NSCLC have achieved a confirmed PR (26.9% ORR by RECIST v1.1) which is comparable to the reported efficacy of other PD-1 inhibitors in this setting. This response in participants with NSCLC in the GARNET study was observed even with negative enrichment by TPS.

Although there are limited clinical data with dostarlimab in combination with chemotherapy agents, a number of clinical studies have been exploring the value of immune checkpoint inhibitors in combination with chemotherapy agents. The combination has been well tolerated and has shown encouraging antitumor activity in patients with previously untreated, metastatic NSCLC (Langer, Gadgeel et al. 2016, Gandhi, Rodríguez-Abreu et al. 2018, Papadimitrakopoulou, Cobo et al. 2018, Paz-Ares, Luft et al. 2018, Socinski, Jotte et al. 2018, West, McCleod et al. 2019).

The efficacy of pembrolizumab in combination with pemetrexed and platinum chemotherapy was investigated in patients with untreated metastatic non-squamous NSCLC (without EGFR or ALK mutations) in the KEYNOTE-189 study. The study demonstrated a significant improvement in OS and PFS for pembrolizumab administered in combination with pemetrexed and platinum chemotherapy compared with placebo and chemotherapy alone (Gandhi, Rodríguez-Abreu et al. 2018, KEYTRUDA Prescribing Information 2020, KEYTRUDA Summary of Product Characteristics 2020).

In the pivotal study KEYNOTE-407, pembrolizumab was studied in combination with carboplatin-paclitaxel (or nab-paclitaxel) in first-line metastatic squamous NSCLC. Data from the study demonstrated that adding pembrolizumab to carboplatin-paclitaxel (or nab-paclitaxel) significantly improved the median OS, regardless of PD-L1 tumor expression in patients with metastatic squamous NSCLC (Paz-Ares, Luft et al. 2018).

Indirect comparison of the preliminary efficacy and safety profile of dostarlimab monotherapy from the GARNET study and the data of pembrolizumab monotherapy in second-line NSCLC (Herbst, Baas et al. 2016) indicates that the 2 PD-1 inhibitors may produce similar anti-tumor activity with comparable safety profiles. This study will provide a direct comparison of the efficacy, safety, and tolerability of dostarlimab versus pembrolizumab, both in combination with platinum-based chemotherapy.

2.2. Background

2.2.1. Lung Cancer

Lung cancer is the most common cause of cancer mortality globally and the second most common cancer in both men and women. About 13% of all new cancers in the United States (US) are lung cancer . Most recent lung and bronchus cancer incidence rates in the US from the Surveillance, Epidemiology, and End Results program estimate an incidence

rate of 54.9 per 100,000 from 2016 data . The 2 major forms of lung cancer are NSCLC and small cell lung cancer. NSCLC is a heterogeneous disease that consists of adenocarcinoma, large cell carcinoma, and squamous cell carcinoma, and comprises approximately 84% of all lung cancers . The average age of diagnosis is approximately 70 years old. Despite advances in early detection and standard treatment, NSCLC is often diagnosed at an advanced stage, has poor prognosis, and is the leading cause of cancer deaths worldwide . In the US, the 5 year OS rate of all stages of NSCLC is approximately 19%, with advanced patients (Stage IIIB/IV) having a 5 year OS rate of <5% .

Platinum-based doublet chemotherapy, maintenance chemotherapy, and anti-angiogenic agents in combination with chemotherapy have contributed to improved patient outcomes in advanced NSCLC . Until recently, for most patients with NSCLC without targetable oncogene drivers, first-line platinum-based chemotherapy was the only standard treatment approach . However, recent understanding of the interactions between the immune system and tumor growth has led to the development of a new class of immunotherapies, which have now moved to the frontline setting for NSCLC treatment.

Approval of pembrolizumab monotherapy in the US and European Union (EU) in 2016 and 2017, respectively, as the first-line treatment approach for patients with NSCLC whose tumors demonstrate high expression of PD-L1 (TPS ≥50%) led to a change in the lung cancer management paradigm (Reck, Rodriguez-Abreu et al. 2016, Spiess, Dhillon et al. 2016, Herzberg, Campo et al. 2017, KEYTRUDA Summary of Product Characteristics 2020). Following this approval, it was shown that the addition of pembrolizumab to standard pemetrexed and platinum-based chemotherapy resulted in significantly longer OS and PFS than chemotherapy alone in patients with previously untreated metastatic non-squamous NSCLC (without EGFR or ALK mutations) . Similarly, in patients with previously untreated metastatic squamous NSCLC, the addition of pembrolizumab to standard carboplatin and either paclitaxel or nab-paclitaxel also resulted in significantly longer OS and PFS than chemotherapy alone. Based on these results, pembrolizumab is now approved in combination with chemotherapy as first-line treatment of patients with metastatic non-squamous (with no EGFR or ALK genomic tumor aberrations) and squamous NSCLC in the US and EU (Spiess, Dhillon et al. 2016, Paz-Ares, Luft et al. 2018, KEYTRUDA Summary of Product Characteristics 2020). Most recently, pembrolizumab monotherapy was approved in the US for use in previously untreated advanced or metastatic NSCLC patients with PD-L1 expression (TPS ≥1%; patients with EGFR or ALK genomic tumor aberrations should have disease progression on an approved therapy prior to receiving pembrolizumab monotherapy) (Mok, Wu et al. 2019).

2.2.2. Programmed Cell Death, Immune Evasion, and Immunotherapy

Programmed cell death protein 1 (PD-1) is a cell surface receptor on activated T cells (Ishida, Agata et al. 1992). PD-1 is part of a complex system of receptors and ligands that are involved in controlling T cell activation. PD-1 has 2 known ligands, PD-L1 (B7-H1) and programmed death-ligand 2 (PD-L2) (B7-DC). The PD-1/PD-L1 checkpoint serves as a negative regulator of T cells to help control local inflammatory responses and maintain self-tolerance. PD-L1 is constitutively expressed on a subset of macrophages but is also expressed by tumor cells . In the tumor microenvironment, PD-L1 expressed on tumor cells binds to PD-1 on activated T cells. This results in T-cell inhibition that

prevents the T cell from killing the tumor cells, a process called immune evasion . This observation is the basis of the hypothesis that the PD-1/PD-L1 signaling pathway may be exploited to regain the antitumor immune response. Nonclinical models in which PD-1 signaling was blocked or deficient (i.e., PD-1 knockout mice) demonstrate improved immune-mediated tumor control (Blank, Brown et al. 2004, Zhang, Gajewski et al. 2009).

The ligands for PD-1 (PD-L1 and PD-L2) are constitutively expressed or can be induced in a variety of cell types . PD-L1 is expressed at low levels on various nonhematopoietic tissues, most notably on the vascular endothelium, whereas PD-L2 protein is predominantly expressed on antigen-presenting cells found in lymphoid tissue or chronic inflammatory environments . The binding of PD-1 ligands to PD-1 inhibits T cell activation triggered through the T cell receptor. PD-L2 is thought to control immune T cell activation in lymphoid organs, whereas PD-L1 serves to dampen unwarranted T cell function in peripheral tissues. Although healthy organs express little (if any) PD-L1, a variety of cancers were shown to express abundant levels (Karim, Jordanova et al. 2009, Taube, Anders et al. 2012) which, via its interaction with the receptor, plays a critical role in immune evasion by tumors . As a consequence, the PD-1/PD-L1 pathway is an attractive target for therapeutic treatment in cancer .

2.2.3. Overview of PD-1 Inhibitors

The recognition of tumors by the immune system has been appreciated for multiple decades and provides an impetus to utilize the immune system to control tumor growth. Studies have reported the presence of tumor-infiltrating lymphocytes as a positive prognostic feature in multiple tumors, supporting a role for the immune system in limiting tumor growth. Despite evidence of immune reactivity, tumors are able to grow in the presence of an immune system, suggesting a suboptimal immune response (Zhang, Conejo-Garcia et al. 2003, Pages, Berger et al. 2005, Fridman, Pages et al. 2012).

The PD-1 receptor-ligand interaction is a major pathway hijacked by tumors to suppress immune control (Pedoeem, Azoulay-Alfaguter et al. 2014). The normal function of PD-1, expressed on the cell surface of activated T cells under healthy conditions, is to down-modulate unwanted or excessive immune responses, including autoimmune reactions. PD-1 (encoded by the gene PDCD1) is an immunoglobulin superfamily member related to cluster of differentiation (CD) 28 and cytotoxic T-lymphocyte-associated protein 4 (CTLA-4), which has been shown to negatively regulate antigen receptor signaling upon engagement of its ligands. PD-1 is expressed on activated lymphocytes, including peripheral CD4+ and CD8+, T cells, B cells, regulatory T cells, and natural killer cells . Expression has also been shown during thymic development on CD4/CD8 (double-negative) T cells, as well as subsets of macrophages and dendritic cells (Nishimura, Agata et al. 1996, Huang, Venet et al. 2009, Pena-Cruz, McDonough et al. 2010).

The first-in-human report of inhibition of the PD-1/PD-L1 pathway was in 2010. Antitumor activity was observed in patients with various solid tumors upon administration of nivolumab, an anti-PD-1 antibody (Brahmer, Drake et al. 2010). The broad spectrum of cancers amenable to PD-1 blockade supports the development of an antibody that targets PD-1 as a single agent, and in combination with other therapeutic agents.

Multiple clinical studies in patients with melanoma, renal cell carcinoma, NSCLC, and other cancer types have generated positive data with 2 different antibodies targeting PD-1, nivolumab and pembrolizumab, demonstrating improved response rates and survival (Borghaei, Paz-Ares et al. 2015, Motzer, Escudier et al. 2015, Robert, Long et al. 2015, Robert, Schachter et al. 2015, Weber, D'Angelo et al. 2015). Based on the clinical activity and manageable safety profile reported in these pioneering studies, nivolumab and pembrolizumab both have received approval from the US Food and Drug Administration and European Medicines Agency in a number of indications (refer to approved prescribing information).

2.2.4. PD-1 Inhibitors in Combination with Chemotherapy

There is accumulating evidence that, in addition to direct cytostatic and cytotoxic effects, the mechanisms of action of conventional chemotherapies may also involve activation of tumor-targeted immune responses, including increasing the immunogenicity of cancer cells and reducing immunosuppression of tumors (Kroemer, Galluzzi et al. 2013, Hato, Khong et al. 2014). In nonclinical models, platinum-based agents have been shown to modulate T cell activation by several potential mechanisms, including down-regulation of PD-L2, increased adenosine triphosphate, and increased high mobility group protein box 1 release from dying cells (Senovilla, Vitale et al. 2012). Several immunostimulatory effects of paclitaxel on the immune system have also been reported; among these are the induction of endoplasmic reticulum stress, which can lead to calreticulin exposure and dendritic cell stimulation (Senovilla, Vitale et al. 2012), depletion of myeloid-derived suppressor cells (Sevko, Michels et al. 2013, Liechtenstein, Perez-Janices et al. 2014), and boosting of T cell priming (Pfannenstiel, Lam et al. 2010). These data suggest that chemotherapy agents may modulate the tumor microenvironment, an effect that could be enhanced by the addition of immune checkpoint inhibitors, such as those targeting PD-1.

The ongoing Phase 1/2 dose escalation and cohort expansion study (Study 4010-01-001 [GARNET; GSK Study 213346]), which is evaluating dostarlimab as monotherapy, has shown promising preliminary efficacy data in 67 participants with previously treated advanced NSCLC; 18 out of 67 NSCLC patients have achieved a confirmed PR (26.9% ORR by RECIST v1.1), which is comparable to the reported efficacy of other PD-1 inhibitors in this setting. This response participants with NSCLC in the GARNET study was observed even with negative enrichment by TPS.

Although there are limited clinical data with dostarlimab in combination with chemotherapy agents, a number of clinical studies have been exploring the value of immune checkpoint inhibitors in combination with chemotherapy agents. The combination has been well tolerated and has shown encouraging antitumor activity in patients with previously untreated, metastatic NSCLC (Langer, Gadgeel et al. 2016, Gandhi, Rodríguez-Abreu et al. 2018, Papadimitrakopoulou, Cobo et al. 2018, Paz-Ares, Luft et al. 2018, Socinski, Jotte et al. 2018, West, McCleod et al. 2019).

The efficacy of pembrolizumab in combination with pemetrexed and platinum chemotherapy was investigated in patients with untreated metastatic non-squamous NSCLC (without EGFR or ALK mutations) in the KEYNOTE-189 study. The study demonstrated a significant improvement in OS and PFS for pembrolizumab administered in combination with pemetrexed and platinum chemotherapy compared with placebo and chemotherapy alone. After a median follow-up of 10.5 months, the estimated OS rate at

12 months was 69.2% (95% CI, 64.1% to 73.8%) in the pembrolizumab-chemotherapy combination arm versus 49.4% (95% CI, 42.1% to 56.2%) in the placebo-chemotherapy arm (hazard ratio [HR] for death, 0.49; 95% CI, 0.38 to 0.64; $p<0.001$). The median OS was not reached in the pembrolizumab-chemotherapy arm. Median PFS was 8.8 months (95% CI, 7.6 months to 9.2 months) in the pembrolizumab-chemotherapy arm and 4.9 months (95% CI, 4.7 months to 5.5 months) in the placebo-chemotherapy arm (HR for disease progression or death, 0.52; 95% CI, 0.43 to 0.64; p<0.001). The response rate as assessed by BICR was 47.6% (95% CI, 42.6% to 52.5%) in the pembrolizumab-chemotherapy arm and 18.9% (95% CI, 13.8% to 25.0%) in the placebo-chemotherapy arm (p<0.001). Adverse reactions leading to discontinuation of pembrolizumab occurred in 20% of patients. The most common AEs (≥1%) leading to discontinuation were pneumonitis (3%) and acute kidney injury (2%). Adverse reactions leading to the interruption of pembrolizumab occurred in 53% of patients; the most common adverse reactions or laboratory abnormalities leading to interruption of pembrolizumab (≥2%) were neutropenia (13%), asthenia/fatigue (7%), anemia (7%), thrombocytopenia (5%), diarrhea (4%), pneumonia (4%), increased blood creatinine (3%), dyspnea (2%), febrile neutropenia (2%), upper respiratory tract infection (2%), increased alanine aminotransferase (ALT) (2%), and pyrexia (2%). AEs leading to death were 6.7% in pembrolizumab-chemotherapy arm versus 5.9% in placebo placebo-chemotherapy arm; 3 immune-mediated AEs (all pneumonitis) led to death in the pembrolizumab-chemotherapy arm . Pembrolizumab in combination with platinum and pemetrexed has since been approved in the US and EU for the treatment of metastatic, first-line, non-squamous NSCLC (Spiess, Dhillon et al. 2016, KEYTRUDA Prescribing Information 2020, KEYTRUDA Summary of Product Characteristics 2020).

In the pivotal study KEYNOTE-407, pembrolizumab was studied in combination with carboplatin-paclitaxel (or nab-paclitaxel) in first-line metastatic squamous NSCLC (Paz-Ares, Luft et al. 2018). Data from the study demonstrated that adding pembrolizumab to carboplatin-paclitaxel (or nab-paclitaxel) significantly improved median OS regardless of PD-L1 tumor expression in patients with metastatic squamous NSCLC. The median OS was 15.9 months (95% CI, 13.2 month to not evaluable) for the pembrolizumab-chemotherapy arm versus 11.3 months (95% CI, 9.5 months to 14.8 months) for the placebo-chemotherapy arm (HR, 0.64; 95% CI, 0.49 to 0.85; $p<.001$). Consistent with OS, pembrolizumab-chemotherapy had significantly improved median PFS over chemotherapy alone. The median PFS was 6.4 months (95% CI, 6.2 months to 8.3 months) for the pembrolizumab-chemotherapy arm compared with 4.8 months (95% CI, 4.3 months to 5.7 months) for the placebo-chemotherapy arm (HR, 0.56; 95% CI, 0.45 to 0.70; $p<.001$). The response rate, as assessed by means of BICR, was 57.9% (95% CI, 51.9% to 63.8%) in the pembrolizumab-chemotherapy arm and 38.4% (95% CI, 32.7% to 44.4%) in the placebo-chemotherapy arm. The frequency of AEs was mostly similar between the pembrolizumab-chemotherapy and placebo-chemotherapy arms, both for overall events (98.2% vs 97.9%) and Grades 3 to 5 events (69.8% vs 68.2%). The most common AEs in both arms included anemia, alopecia, neutropenia, nausea, and thrombocytopenia, with cytopenias comprising the majority of Grade 3 to 5 AEs in each arm. Immune-mediated AEs and infusion reactions did occur more frequently with the addition of pembrolizumab to chemotherapy, compared with both overall (28.8% vs 8.6%) and for Grades 3 to 5 AEs (10.8% vs 3.2%). The most common immune-mediated AEs (incidence ≥5%) associated with the pembrolizumab regimen included

hypothyroidism (7.9%), hyperthyroidism (7.2%), and pneumonitis (6.5%). AEs leading to death were 8.3% in the pembrolizumab-chemotherapy arm vs 6.4% in the chemotherapy arm; 1 patient in each arm died from an immune-mediated AE (pneumonitis). Importantly, no new safety signals were identified, and the safety profile of pembrolizumab in combination with chemotherapy was consistent with that previously observed in lung cancer with pembrolizumab monotherapy. Pembrolizumab and carboplatin-paclitaxel (or nab-paclitaxel) combination treatment has since been approved in the US and EU for the treatment of metastatic first-line squamous NSCLC .

2.2.5. Dostarlimab

Dostarlimab (also known as TSR-042) is an immunoglobulin G (IgG)4-kappa humanized monoclonal antibody (mAb) that binds with high affinity to PD-1, resulting in inhibition of binding to PD-L1 and PD-L2. This antibody was generated based on a proprietary platform that utilizes affinity maturation to select antibodies with desired functional characteristics. The functional antagonist activity of dostarlimab was confirmed in a mixed lymphocyte reaction assay demonstrating enhanced interleukin-2 production upon addition of dostarlimab.

The overall immunogenicity risk for dostarlimab is low. The dostarlimab high purity drug product and route of administration decrease the risk for induction of immune responses. The current clinical data are consistent with this assessment; post-dostarlimab antibody responses to date have been low in incidence and with no apparent impact on PK, safety, or efficacy.

The dostarlimab development program includes several ongoing studies of dostarlimab as monotherapy and in combination with chemotherapy and other immuno-oncology agents across multiple tumor types.

<u>Monotherapy</u>

- Study 4010-01-001 (GARNET; GSK Study 213346) (Investigational New Drug [IND] 126472), is a Phase 1/2 dose escalation and cohort expansion (multicenter, open-label, first-in-human) study of dostarlimab as monotherapy in participants with advanced solid tumors, including NSCLC.

<u>Combination Therapy</u>

- Study 3000-01-002 (IOLite; GSK Study 213351) (IND 126472), is a multi-arm Phase 1 study of dostarlimab in various combinations with niraparib, cobolimab, bevacizumab and chemotherapies, including carboplatin, paclitaxel, and pemetrexed in advanced solid tumors, including NSCLC.
- Study 4020-01-001 (AMBER; GSK Study 213348) (IND 129161), is a Phase 1 study of dostarlimab and TSR-022/cobolimab (anti-T-cell immunoglobulin and mucin-domain containing-3 [TIM-3]) combination therapy in advanced solid tumors.
- Study 4040-01-001 (CITRINO; GSK Study 213349) (IND 134547), is a Phase 1 study of dostarlimab and TSR-033 (anti-lymphocyte-activation gene 3 [LAG-3]) combination therapy in advanced solid tumors.

- Study 3000-02-001 (JASPER; GSK Study 213352) (IND 134426), is a Phase 2 multi-arm study of dostarlimab in combination with niraparib in participants with NSCLC.
- Study 3000-03-005 (FIRST; GSK Study 213350) (IND 126472), is a Phase 3, randomized, double-blind study comparing platinum-based therapy with dostarlimab and niraparib versus standard-of-care platinum-based therapy as first-line treatment of ovarian cancer
- Study 3000-02-005 (OPAL; GSK Study 213357) (IND 100996), is a Phase 2 multi-cohort study to evaluate the safety and efficacy of novel treatment combination in participants with recurrent ovarian cancer.
- Study 3000-02-006 (MOONSTONE; GSK Study 213353) (IND 100996), is a Phase 2 single-arm study of niraparib in combination with dostarlimab to evaluate the efficacy and safety in participants with advanced, relapsed, high-grade ovarian cancer who have platinum-resistant disease or for whom further platinum therapy is not appropriate.
- Study 4010-03-001 (RUBY; GSK Study 213361) (IND 126472), is a Phase 3, randomized, double-blind study comparing dostarlimab in combination with platinum-based therapy versus standard-of-care platinum-based therapy efficacy in participants with recurrent and primary advanced EC.

Study 4010-01-001 (GARNET; GSK 213346) is the most relevant study with respect to this protocol and is discussed in more detail in the following section. Additional information on the clinical development program is contained within the dostarlimab Investigator's Brochure (IB).

2.2.5.1. Efficacy of Dostarlimab in NSCLC

Study 4010-01-001 (GARNET; GSK Study 213346), titled "A Phase 1 Dose Escalation and Cohort Expansion Study of TSR-042, an anti-PD-1 Monoclonal Antibody, in Patients with Advanced Solid Tumors," is a multicenter, open-label, first-in-human, Phase 1/2 study evaluating dostarlimab in participants with advanced solid tumors whose disease has progressed following treatment with available therapies, with dose escalation and fixed-dose safety evaluation phases in participants with advanced solid tumors and a dose expansion phase in specific tumor types, including NSCLC.

Preliminary clinical data from the GARNET study's NSCLC cohort show that dostarlimab monotherapy (500 mg Q3W for first 4 cycles and 1,000 mg every 6 weeks [Q6W] for all subsequent cycles) provides clinical benefits to participants with NSCLC. As of 08 July 2019, 67 participants with previously treated NSCLC were treated and had follow-up of 12 weeks or longer. Eighteen out of 67 participants with NSCLC have achieved a confirmed CR or PR (26.9% ORR by RECIST v1.1). Among these responders 7/18 (38.9%) were still in response. Activity was observed across all PD-L1 TPS categories, and encouraging activity was observed despite the fact that vast majority (90%) of participants with available PD-L1 status had a TPS <50%. As 01 March 2020, there are 7 participants still on treatment, 5 of which are responders.

In summary, in immuno-oncology-naïve participants with previously treated advanced NSCLC, the efficacy of dostarlimab was comparable to the reported efficacy of other

PD-1 inhibitors in similar NSCLC populations despite negative enrichment by TPS (Borghaei, Paz-Ares et al. 2015, Brahmer, Reckamp et al. 2015, Herbst, Baas et al. 2016).

2.2.5.2. Safety of Dostarlimab

Available data suggest that dostarlimab has a comparable safety profile to other PD1 inhibitors, including pembrolizumab. Dostarlimab is being investigated in multiple ongoing studies, including the GARNET study, with approximately 1,000 participants enrolled across different disease types, both in monotherapy and in combination with other therapies. Dostarlimab has demonstrated an acceptable safety profile with manageable toxicity. The observed AEs were in line with those expected in patients with recurrent or advanced solid tumors and were consistent with reported safety profiles of mAbs blocking the PD-L1 interactions, including pembrolizumab . Detailed information about the safety of dostarlimab is included in the IB.

Dostarlimab in NSCLC

As of the data cutoff date of 01 March 2020, 67 participants with NSCLC had been treated with dostarlimab monotherapy in the GARNET study. Within this population, all participants experienced treatment-emergent adverse events (TEAEs) (100%) and 55% (n=37) experienced any Grade ≥3 TEAEs. The rate of serious adverse events (SAEs) was 43.3% (n=29) and the rate of TEAEs leading to dostarlimab discontinuation was 10.4% (n=7). However, the rates of dostarlimab-related Grade ≥3 TEAEs was 13.4% (n=9) and dostarlimab-related SAEs (7.5%; n=5) were slightly lower than the overall rates. The rate of discontinuation due to dostarlimab-related TEAEs was low (10.4%; n=7) and there were no dostarlimab-related TEAEs leading to death.

In the NSCLC group, 43.3% (n=29) of participants experienced any immune-related adverse event (irAE) and 7.5% (n=5) experienced Grade ≥3 irAEs that were determined to be related to dostarlimab. All of the dostarlimab-related Grade ≥3 irAEs occurred in 1.5% of participants. Neither Grade ≥3 pneumonitis and or colitis occurred in any participants in this group.

Dostarlimab as Monotherapy

As of the data cutoff date of 01 March 2020, all of the 515 participants enrolled in GARNET who had received at least 1 dose of dostarlimab are included in the Safety Population. Dostarlimab was generally safe with manageable toxicity. The majority of participants reporting TEAEs did not experience serious TEAEs and did not experience TEAEs requiring treatment discontinuation. TEAEs reported in >20% of participants were anemia, fatigue, nausea, and diarrhea. Grade ≥3 TEAEs were reported in 50.3% of the Safety Population. Immune-related TEAEs (Grade ≥2 based on a predefined list) were reported in 34.8% of participants, and immune-related TEAEs related to dostarlimab were reported in 22.7% of the Safety Population. Serious immune-related TEAEs were reported in 5.0% of participants. TEAEs leading to death were reported in 2.7% of participants. No deaths resulting from TEAEs that were assessed as related to dostarlimab were reported.

Dostarlimab as Combination Therapy

As of the IB data cutoff date of 21 January 2020, the population of participants who received dostarlimab with chemotherapy was 27 participants, as compared to 535

participants who received monotherapy. Those participants received dostarlimab in combination with a variety of chemotherapy regimens, including, but not limited to: carboplatin, paclitaxel, and bevacizumab; FOLFIRI and bevacizumab; and FOLFOX and bevacizumab. Rates of any TEAE were similar between the 2 groups. Rates of any Grade ≥3 TEAEs and any SAEs were higher with combination therapy. However, rates of dostarlimab-related Grade ≥3 TEAEs were similar between the groups and dostarlimab-related SAEs were slightly lower in the combination group. Rates of discontinuation due to dostarlimab-related TEAEs were low in both groups. Rates of irAEs were also similar in the combination therapy and monotherapy therapy groups.

For further safety information, refer to the current version of the IB.

2.2.6. Pembrolizumab

Pembrolizumab is a potent humanized IgG4 mAb with high specificity of binding to PD-1 receptor, thus inhibiting its interaction with PD-L1 and PD-L2 ligands. Based on nonclinical in vitro data, pembrolizumab has high affinity and potent receptor blocking activity for PD-1.

2.2.6.1. Pembrolizumab in NSCLC

Pembrolizumab (KEYTRUDA®) is approved in the US and EU for multiple NSCLC indications . As described in Section 2.2.4, the safety of pembrolizumab in combination with chemotherapy was determined in patients with advanced non-squamous NSCLC who had documented disease progression following treatment with platinum-based chemotherapy in the KEYNOTE-189 study.

For more details, refer to the approved prescribing information for pembrolizumab.

2.3. Benefit/Risk Assessment

More detailed information about the known and expected benefits and risks and reasonably expected AEs of dostarlimab may be found in the IB. More detailed information regarding the expected benefits and risks of pembrolizumab can be found in local approved product labels (KEYTRUDA Prescribing Information 2020, KEYTRUDA Summary of Product Characteristics 2020).

2.3.1. Risk Assessment

Table 5: Risk Assessment

Potential Risk of Clinical Significance	Summary of Data/Rationale for Risk	Mitigation Strategy
**Dostarlimab and Pembrolizumab**		
IRRs	IRRs are a risk for all PD-(L)1 inhibitors.<br>Infusion-related reactions, which may be severe, have been reported in association with dostarlimab and pembrolizumab.	Participants will be closely monitored for signs of IRR.<br>For severe (Grade 3) or life-threatening (Grade 4) IRRs associated with dostarlimab, infusion should be stopped, and treatment should be permanently discontinued.<br>Management of IRRs is provided in Table 14.
irAEs	irAEs, which may be severe or fatal, can occur in patients treated with monoclonal antibodies directed against immune checkpoints, including pembrolizumab and dostarlimab. While irAEs (e.g., diarrhea/colitis, pneumonitis, nephritis, hypophysitis, adrenalitis, thyroiditis, severe skin reactions, uveitis, myocarditis, and hepatotoxicity) usually occur during treatment, symptoms can also manifest after discontinuation of treatment. irAEs may occur in any organ or tissue and may affect more than one body system simultaneously.	Participants with the following medical history are ineligible for this study:<br>• Toxicity (Grade 3) related to prior immunotherapy leading to study treatment discontinuation<br>• Active autoimmune disease<br>• Severe hypersensitivity to another mAb<br>Established management algorithms for irAEs: refer to Section 6.4.2.1 for further details on the identification, evaluation, and management of toxicities, including cumulative effects, with a potential immune etiology.

Potential Risk of Clinical Significance	Summary of Data/Rationale for Risk	Mitigation Strategy
Participants exposed to ionizing radiation as a consequence of participation in this study. Some of the radiation exposure required by the study is additional to routine clinical care.	The ionizing radiation exposure arises due to the inclusion in the study of CT scans.<br>The risk arising from the additional radiation burden is considered justified by the benefit of the clinical information to be obtained, which is critical to the study and cannot readily be obtained another way. The effective dose of radiation for these procedures is approximately 20 mSv per combined CT examination of the Chest and Abdomen. For comparison, the average global yearly background radiation dose is 2.4 mSv.	The study has been designed to keep the radiation dose as low as reasonably practicable (ALARP) whilst obtaining images of sufficient quality to meet the objectives.

Abbreviations: AE=adverse event; irAE=immune-related adverse event; IRR=infusion-related reaction; mAb=monoclonal antibody; PD-(L)1=programmed cell death protein 1/(programmed death-ligand 1).

2.3.2. Benefit Assessment

Preliminary clinical data from Study 4010-01-001 (GARNET; GSK Study 213346) show that dostarlimab monotherapy provides clinical benefits to participants with NSCLC. As of the 08 July 2019 data cutoff, 67 participants with NSCLC were treated and had follow-up of 12 weeks or longer. Eighteen out of 67 participants with NSCLC have achieved a confirmed CR or PR (26.9% ORR by RECIST v1.1. Among these responders, 7 out of 18 (38.9%) were still in response. Activity was observed across all PD-L1 TPS categories, and encouraging activity was observed despite the fact that the vast majority (90%) of participants with available PD-L1 status had a TPS <50%.

In summary, in participants with previously treated advanced NSCLC, the efficacy of dostarlimab was comparable to the reported efficacy of other PD-1 inhibitors in similar NSCLC populations despite negative enrichment by TPS (Borghaei, Paz-Ares et al. 2015, Herbst, Baas et al. 2016, Borghaei, Langer et al. 2019).

2.3.3. Overall Benefit/Risk Conclusion

Considering the measures taken to minimize risk to participants taking part in this study, the potential risks identified in association with dostarlimab are justified by the anticipated benefits that may be afforded to participants with NSCLC.

3. OBJECTIVES AND ENDPOINTS

Table 6: Objectives and Endpoints for Study 213403

Objectives	Endpoints
**Primary**	
• To compare the ORR of PD-1 inhibitor dostarlimab vs pembrolizumab administered in combination with chemotherapy as evaluated using RECIST v1.1 based on BICR in participants with metastatic non-squamous NSCLC, without a known EGFR, ALK, ROS-1, or BRAF V600E mutation or other genomic aberration for which a targeted therapy is available, who have received no prior treatment of metastatic disease	• The primary efficacy endpoint ORR will be evaluated by RECIST v1.1 based on BICR and will be defined as the proportion of participants with BOR of CR or PR in the analysis population.
**Secondary**	
• To evaluate the following measures of clinical benefit of PD-1 inhibitor administered in combination with chemotherapy:<br>– OS<br>– PFS evaluated using RECIST v1.1 based on Investigator assessment	• OS will be defined as the time from the date of randomization to the date of death by any cause.<br>• PFS will be evaluated using RECIST v1.1 based on Investigator assessment and will be defined as the time from the date of randomization to the date of PD or death by any cause, whichever occurs first.
• To evaluate the safety of PD-1 inhibitor in combination with chemotherapy	• Assess the incidence of TEAEs, SAEs, irAEs, TEAEs leading to death, and AEs leading to discontinuation occurring while participants are on treatment or up to 90 days after the last dose of study treatment. Clinical laboratory parameters (hematology, chemistry, thyroid function, urinalysis), vital signs, ECOG performance status, ECG parameters, physical examinations, and usage of concomitant medications will be collected.

Table 6: Objectives and Endpoints for Study 213403 (Continued)

Objectives	Endpoints
CCI [redacted]	[redacted]

Table 6: Objectives and Endpoints for Study 213403 (Continued)

Objectives	Endpoints
CCI [redacted]	[redacted]

Abbreviations: CCI [redacted]; ALK=anaplastic lymphoma kinase; BICR=blinded independent central review; CCI [redacted]; BRAF=proto-oncogene B-raf; CCI [redacted] CR=complete response; CCI [redacted]; ECG=electrocardiogram; ECOG=Eastern Cooperative Oncology Group; EGFR=epidermal growth factor receptor; CCI [redacted] EOT=End-of-Treatment; CCI [redacted] irAE=immune-related adverse event; CCI [redacted]; NSCLC=non-small cell lung cancer; ORR=objective response rate; OS=overall survival; PD=progressive disease; PD-1=programmed cell death protein 1; CCI [redacted]; PFS=progression-free survival; CCI [redacted] PR=partial response; CCI [redacted] RECIST=Response Evaluation Criteria in Solid Tumors; ROS-1=receptor tyrosine kinase-1; SAE=serious adverse event; TEAE=treatment-emergent adverse event; CCI [redacted]

4. STUDY DESIGN

4.1. Overall Design

This is a randomized, Phase 2, double-blind, 2-arm study to compare the efficacy and safety of PD-1 inhibitors dostarlimab and pembrolizumab, when administered in combination with chemotherapy, in male and female participants 18 years and older with non-squamous NSCLC without a known sensitizing EGFR, ALK, ROS-1, or BRAF V600E mutation or other genomic aberration for which an approved targeted therapy is available who have not received previous systemic anticancer therapy for metastatic disease.

The study consists of a Screening Period (Day -28 to Day -1) for completion of all Screening assessments and subsequent randomization, a Treatment Period, an EOT Visit (within 7 days of the decision to discontinue treatment for any reason), a Safety Follow-up Period with a visit at 30 (+7) and 90 (+7) days after the last dose of study treatment, and a Post-treatment Follow-up Period with assessments occurring 180 days after the last dose of study treatment and every 90 (±14) days thereafter, continuing until death, withdrawal of consent, or the end of study data collection. During the Treatment Period, clinic visits will occur Q3W. Collection of AEs begins from the start of study treatment and according to the time points specified in the SoA (Table 4) and Section 7.5.1. Any SAEs assessed as related to study participation (e.g., study treatment, protocol-mandated procedures, invasive tests, or change in existing therapy) or related to a GSK product will be recorded from the time a participant consents to participate in the study.

To be eligible for the study, participants must have documented PD-L1 status by the 22C3 pharmDx assay (Agilent/Dako). If no prior PD-L1 result is available at the time of Screening, the participant can be tested locally using the aforementioned method, or central PD-L1 testing can be completed (refer to the Laboratory Manual for country-specific details of central assessment). For participants requiring PD-L1 testing at the time of study entry, an archival FFPE tumor tissue specimen, which may have been collected at any time prior to Screening, can be submitted for analysis within 28 days of the participant’s first dose. Specimens used to confirm diagnosis or collected after the participant has been diagnosed with metastatic disease will be preferred for determination of PD-L1 status. Both tissue block and freshly cut (within 6 months) slides (10 to 15) are acceptable. If no archival FFPE tumor tissue is available, a fresh tumor tissue biopsy should be obtained and tested within 28 days of the participant’s first dose. Biopsies obtained prior to receipt of adjuvant/neoadjuvant chemotherapy will be permitted if a fresh biopsy is not feasible. As the results of baseline PD-L1 status assessment will be used as a stratification factor in this study, results must be provided prior to randomization. Participants who do not have documented PD-L1 status and cannot provide archived or fresh tissue sample for PD-L1 testing will not be eligible for the study.

Within 35 days prior to the first dose, participants must have a baseline tumor assessment of the chest and abdomen (including the entire liver and both adrenal glands). Other regions should be imaged as clinically indicated (e.g., pelvis, brain, etc). This assessment should be conducted by IV contrast-enhanced computed tomography (CT) or magnetic

resonance imaging (MRI) scan. MRI should be used for abdominal (and pelvic) imaging if CT contrast is contraindicated, and, preferably, for IV contrast-enhanced imaging of the brain. The same imaging technique should be used in a participant throughout the study. The CT component of positron emission tomography (PET)/CT may be used according to RECIST v1.1 guidelines, with full radiation dose diagnostic CT and IV CT contrast, and as clinically indicated. At each post-baseline assessment, evaluations of the sites of the disease identified by these scans are required.

Following informed consent and completion of all Screening assessments, all participants who meet the eligibility criteria will be randomized 1:1 to receive chemotherapy in combination with either dostarlimab or pembrolizumab. Randomization will be completed in a blinded manner using an interactive web response system. Dostarlimab and pembrolizumab must be dispensed in a blinded manner. Details of blinding will be documented in site-specific blinding plans. The dosing regimen is described in Section 6.1.4. Randomization will be stratified by the following 2 factors:

- PD-L1 status of the tumor (TPS <1% vs 1% to 49% vs ≥50%)
- Smoking status (never vs former/current)

To support continued tumor assessment while on study, serial imaging will then be performed at 6 weeks (42 [±7] days) and again at 12 weeks (84 [±7] days) from the randomization date. Following the 12-week scan, imaging will be performed Q9W (every 63 [±7] days), or more frequently, if clinically indicated. After 48 weeks, participants who remain on treatment will have imaging performed Q12W (every 84 [±7] days). Imaging should continue to be performed until discontinuation of study treatment due to disease progression with clinical instability, start of subsequent anticancer treatment, withdrawal of informed consent, or death, whichever comes first. Imaging must be performed on a calendar schedule and should not be affected by dose interruptions/delays. All radiographic images/scans at the specified time points, as well as any unscheduled images/scans, will be collected and stored centrally for BICR, potential future evaluation, and archiving. Digital copies of all DICOM-formatted scans must be maintained at the Investigative site as source documents.

Following the initial radiologic assessment of progressive disease (PD), clinically stable participants should continue study treatment and a follow-up scan should be performed at least 4 weeks and no longer than 8 weeks later. The follow-up scan will provide additional information to the Investigator for participant management and further treatment decisions. Further guidance for the assessment of scans acquired after RECIST v1.1-defined PD are provided in Appendix 1. For participants who are not clinically stable (clinically progressing) at the initial radiologic assessment of PD, study treatment will be discontinued and a follow-up scan is not required. Following the initial radiologic assessment of CR or PR, a confirmatory follow-up scan should be performed at least 4 weeks later.

CCI [redacted]. Both tissue block and freshly cut (within 6 months) slides are acceptable. If archival tissue is not available, the participant can elect to undergo biopsy prior to study

entry. Open biopsies, punch biopsies, and core biopsies (3 samples) are acceptable. Fine-needle aspirate, frozen sample, plastic embedded sample, cell block, clot, and cytological specimen are not acceptable for analysis. The archival tumor tissue sample should be submitted within 28 days of the participant's first dose of study treatment. CCI [redacted]

Blood samples to assess the PK of dostarlimab and pembrolizumab will be collected from all participants during treatment, at EOT, at the 90-day Safety Follow-up Visit, and at the 180-day Post-treatment Follow-up Visit. Immunogenicity with associated drug concentration will be analyzed in participants administered dostarlimab and, if needed, in participants administered pembrolizumab. CCI [redacted]

PROs will be collected in alignment with study treatment administration at scheduled visits during the Treatment Period, at the EOT Visit, and at the 30-day Safety Follow-up Visit.

Biomedical Research

CCI [redacted]

Number of Participants

Approximately 240 participants will be randomly assigned to 2 study treatments, such that approximately 120 evaluable participants in each of the 2 arms complete the study.

Treatment Groups and Duration

Participants will be randomized in a 1:1 ratio into the dostarlimab plus chemotherapy arm or the pembrolizumab plus chemotherapy arm. Prior to randomization, Investigators will

select, based on their clinical judgment, cisplatin or carboplatin as the chemotherapy to be administered. Randomization will be stratified by PD-L1 status of the tumor (TPS <1% versus 1% to 49% versus ≥50%) and smoking status (never vs former/current). A PD-L1 result obtained prior to Screening is allowed.

The study will last approximately 5 years.

4.2. Scientific Rationale for Study Design

The design of the proposed similarity study relies, in part, on the estimated treatment effect of pembrolizumab plus chemotherapy from 3 adequate and well-controlled studies (KEYNOTE studies) where said regimen was shown to be superior to chemotherapy alone (Langer, Gadgeel et al. 2016, Gandhi, Rodríguez-Abreu et al. 2018, Paz-Ares, Luft et al. 2018). The robustness of these studies in design and conduct help provide a reliable estimate of pembrolizumab's effect. The proposed study follows KEYNOTE studies with respect to important study design features, including patient population and dosing of reference products.

4.3. Justification for Dose

As determined in the Phase 1/2 Study 4010-01-001 (GARNET; GSK Study 213346), the recommended clinical dose of dostarlimab deemed safe and effective is 500 mg Q3W for 4 cycles, followed by 1,000 mg Q6W. To allow for comparison between the 2 arms of this study, dostarlimab dosing will remain at 500 mg Q3W dosing for all cycles (up to 35 total) to align with Q3W dosing of the comparator regimen.

Additional dose justification information for dostarlimab can be found in the IB.

4.4. End of Study Definition

The end of the study is defined as 5 years after the first participant received the first dose of study treatment.

A participant is considered to have completed the study if he/she has completed all phases of the study including the last visit or meets the criteria in Section 5.8.

5. SELECTION OF STUDY POPULATION AND WITHDRAWAL CRITERIA

Prospective approval of protocol deviations to recruitment and enrollment criteria, also known as protocol waivers or exemptions, is not permitted.

5.1. Inclusion Criteria

Participants are eligible to be included in the study only if all of the following criteria apply:

1. Participant must be ≥18 years old, must be able to understand the study procedures, and agrees to participate in the study by providing written informed consent (as described in Appendix 4), which includes compliance with the requirements and restrictions listed in the ICF and in this protocol.
2. Participant has histologically- or cytologically-confirmed metastatic non-squamous NSCLC with documented absence of a sensitizing EGFR, ALK, ROS-1, or BRAF V600E mutation or other genomic aberration for which an approved targeted therapy is available. Mixed tumors will be categorized by the predominant cell type; if the tumor has predominantly squamous cell histology or if small cell elements are present, the participant is ineligible.
3. Participants must have measurable disease, i.e., presenting with at least 1 measurable lesion per RECIST v1.1 as determined by the local site Investigator/radiology assessment. Measurable lesions situated in a previously irradiated area may be considered target lesions if progression has been demonstrated in such lesions and if there are other target lesions. If there is only 1 target lesion that was previously irradiated, the participant is not eligible. See Appendix 1 for the definition of a measurable lesion.
4. Participant has documented PD-L1 status by the 22C3 pharmDx assay (Agilent/Dako). If no prior PD-L1 result is available at the time of Screening, the participant can be tested locally using the stated method, or central PD-L1 testing can be completed. Results are needed for stratification and must be available prior to randomization.
5. Participant has an Eastern Cooperative Oncology Group (ECOG) performance status score of 0 or 1.
6. Participant has a life expectancy of at least 3 months.
7. Participant has adequate organ function as defined in Table 7. (Note: A complete blood count test should be obtained without transfusion or receipt of colony-stimulating factors within 2 weeks of obtaining the sample.)

Table 7: Adequate Baseline Organ Function Inclusion Criteria

System	Laboratory Values	Notes
**Hematologic**		
ANC	≥1.5×10^9/L	
Hemoglobin	≥9 g/dL	
Platelets	≥100×10^9/L	
**Hepatic**		
ALT	See Exclusion Criterion 5.	
Bilirubin		
**Renal**		
Serum creatinine<br>(OR creatinine clearance)	≤1.5×ULN<br>(OR ≥50 mL/min)	Creatinine clearance calculated by the Cockcroft-Gault formula (or measured using 24-hour creatinine clearance) for participants with creatinine levels >1.5×institutional ULN

Abbreviations: ALT=alanine aminotransferase; ANC=absolute neutrophil count; ULN=upper limit of normal.

8. Participant has recovered to Grade ≤1 from any prior treatment-related toxicities at the time of randomization. A participant with Grade 2 alopecia is an exception to this criterion and may qualify for this study.
9. Contraceptive use by male and female participants should be consistent with local regulations regarding the methods of contraception for those participating in clinical studies.
   a. Male participants are eligible to participate if they agree to the following during the Treatment Period and for at least 180 days after the last dose of study treatment. (Note: duration of contraceptive use after last dose of chemotherapy must be consistent with local requirements and local approved product labels; however, <u>the minimum duration is 180 days after last dose of chemotherapy</u>)
   - Refrain from donating sperm

     PLUS, either:
   - Be abstinent from heterosexual intercourse as their preferred and usual lifestyle (abstinent on a long term and persistent basis) and agree to remain abstinent.

     OR
   - Must agree to use contraception/barrier as follows:
     - Agree to use a male condom (and should also be advised of the benefit for a female partner to use a highly effective method of contraception, as a

condom may break or leak) when having sexual intercourse with a woman of childbearing potential (WOCBP) who is not currently pregnant.

  – Agree to use a male condom when engaging in any activity that allows for passage of ejaculate to another person.

b. A female participant is eligible to participate if she is not pregnant or breastfeeding, and 1 of the following conditions applies:

- Is a woman of non-childbearing potential (WONCBP), as defined in Appendix 5.

  OR

- Is a WOCBP, as defined in Appendix 5, using a contraceptive method that is highly effective (with a failure rate of <1% per year and, preferably, with low user dependency, as described in Appendix 5) during the Treatment Period and for at least 180 days after the last dose of study treatment and agrees not to donate eggs (ova or oocytes) for the purpose of reproduction during this period. (Note: duration of contraceptive use after last dose of chemotherapy may be longer than 180 days in order to comply with local requirements and local approved product labels). Contraceptive use should be consistent with local regulations regarding the methods of contraception for those participating in clinical studies. The Investigator should evaluate the potential for contraceptive method failure (e.g., noncompliance and recently initiated) in relationship to the first dose of study treatment.

  – A WOCBP must have a negative highly sensitive pregnancy test (urine or serum, as required by local guidelines) within 72 hours before the first dose of study treatment. If a urine test cannot be confirmed as negative (e.g., an ambiguous result), a serum pregnancy test is required. In such cases, the participant must be excluded from participation if the serum pregnancy result is positive.

  Note: Additional requirements for pregnancy testing during and after study treatment are located in Section 7.4.7.

  Note: The Investigator is responsible for review of medical history, menstrual history, and recent sexual activity to decrease the risk for inclusion of a woman with an early undetected pregnancy

5.2. Exclusion Criteria

Participants are excluded from the study if any of the following criteria apply:

1. Participant has received prior systemic therapy for the treatment of metastatic NSCLC. Participants who have received neoadjuvant or adjuvant chemotherapy are eligible if the neoadjuvant/adjuvant therapy was completed at least 12 months prior to the development of metastatic disease.
2. Participant has received prior therapy with a PD-(L)1 or PD-L2 inhibitor, a CTLA-4 inhibitor, a TIM-3 inhibitor, or any other immunotherapy agent (e.g., OX40) for the treatment of cancer.

3. Participant has received radiation to the lung that is >30 Gy within 6 months of the first dose of study treatment.
4. Participant has completed palliative radiotherapy within 7 days of the first dose of study treatment.
5. Participant is ineligible if any of the following hepatic characteristics are present:
    a. ALT >2.5×upper limit of normal (ULN) without liver metastases/tumor infiltration
    b. ALT >5×ULN with liver metastases/tumor infiltration
    c. Bilirubin >1.5×ULN (isolated bilirubin >1.5×ULN is acceptable if bilirubin is fractionated and direct bilirubin is <35%)
    d. Current active liver or biliary disease (with the exception of Gilbert's syndrome or asymptomatic gallstones, liver metastases, or otherwise stable chronic liver disease per Investigator assessment)

    Note: Stable chronic liver disease should generally be defined by the absence of ascites, encephalopathy, coagulopathy, hypoalbuminemia, esophageal or gastric varices, persistent jaundice, or cirrhosis
6. Participant has a corrected QT interval (QTc) >450 msec (or QTc >480 msec for participants with bundle branch block).

    Notes:

    - The QTc is the QT interval corrected for heart rate according to Bazett's formula (QTcB), Fridericia's formula (QTcF), and/or another method, machine-read or manually over-read.
    - The specific formula that will be used to determine eligibility and discontinuation for an individual participant should be determined prior to initiation of the study. In other words, several different formulae cannot be used to calculate the QTc for an individual participant and then the lowest QTc value used to include or discontinue the participant from the study.
    - For purposes of data analysis, QTcB, QTcF, another QT correction formula, or a composite of available values of QTc will be used as specified in the Reporting and Analysis Plan (RAP).
7. Participant has had major surgery within 3 weeks of the first dose of study treatment or has not adequately recovered from any AEs (Grade ≤1) and/or complications from any major surgery. Surgical implantation of a port catheter is not exclusionary.
8. Participant has an additional malignancy or a history of prior malignancy, with the exception of adequately treated basal or squamous skin cancer, cervical carcinoma in situ, superficial bladder cancer without evidence of disease, other in situ cancers, or had a malignancy treated with curative intent and with no evidence of disease recurrence for 5 years since the initiation of that therapy.
9. Participant has known active brain metastases and/or leptomeningeal metastases. Participants who have received prior therapy for their brain metastases and have radiographically stable central nervous system disease may participate, provided

they are neurologically stable for at least 2 weeks before study entry and must be off corticosteroids within 3 days prior to the first dose of study treatment. Stable brain metastases by this definition should be established prior to the first dose of study treatment. Participants with known untreated, asymptomatic brain metastases (i.e., no neurological symptoms, no requirements for corticosteroids, no or minimal surrounding edema, and no lesions >1.5 cm) may participate, but will require regular imaging of the brain as a site of disease.

10. Participant has tested positive for the presence of hepatitis B surface antigen or has a positive hepatitis C antibody test result at Screening, or within 3 months prior to first dose of study treatment.
11. Participant has an active infection requiring systemic therapy within 1 week prior to the anticipated first dose of study treatment.
12. Participant has known HIV (positive for HIV-1 or HIV-2 antibodies).
13. Participant has active autoimmune disease that required systemic treatment in the past 2 years, is immunocompromised in the opinion of the Investigator, or is receiving systemic immunosuppressive treatment. (Note: Participants with splenectomy are allowed.) Replacement therapy (e.g., thyroxine, insulin, or physiologic corticosteroid replacement therapy for adrenal or pituitary insufficiency, etc) is not considered a form of systemic treatment.
14. Participant has received systemic steroid therapy within 3 days prior to the first dose of the study treatment or is receiving any other form of immunosuppressive medication. Replacement therapy is not considered a form of systemic therapy. Use of inhaled corticosteroids, local steroid injection, or steroid eye drops is allowed.
15. Participant has symptomatic ascites or pleural effusion. A participant who is clinically stable following treatment of these conditions (including therapeutic thoraco- or paracentesis) is eligible.
16. Participant has current interstitial lung disease, current pneumonitis, or a history of pneumonitis that required the use of oral or IV glucocorticoids to assist with management. Lymphangitic spread of the NSCLC is not exclusionary.
17. Participant has a history or current evidence of any medical condition, therapy, or laboratory abnormality that might confound the study results, interfere with their participation for the full duration of the study treatment, or indicate it is not in the best interest of the participant to participate, in the opinion of the Investigator.
18. Participant has clinically active diverticulitis, intra-abdominal abscess, gastrointestinal obstruction, or peritoneal carcinomatosis.
19. Participant has pre-existing peripheral neuropathy that is Grade ≥2 by National Cancer Institute-Common Terminology Criteria for Adverse Events (NCI-CTCAE) v5.0 criteria.
20. Participant has received a live vaccine within 30 days of the first dose of study treatment. Seasonal flu vaccines that do not contain live virus are permitted.

21. Participant does not meet requirements per local prescribing guidelines for receiving treatment with either pemetrexed and cisplatin or carboplatin.
22. Participant has sensitivity to any of the study treatments, or components thereof, or a history of drug or other allergy that, in the opinion of the Investigator or GSK Medical Monitor, contraindicates their participation.
23. Participant is unable to interrupt aspirin or other nonsteroidal anti-inflammatory drugs (NSAIDs), other than an aspirin dose ≤1.3 g per day, for a 5-day period (8-day period for long-acting agents, such as piroxicam).

5.3. Lifestyle and/or Dietary Considerations

There are no identified lifestyle and/or dietary considerations or restrictions for this study.

5.4. Screen Failures

Screen failures are defined as participants who consent to participate in the clinical study but are not subsequently randomized into the study. A minimal set of screen failure information is required to ensure transparent reporting of screen failure participants to meet the Consolidated Standards of Reporting Trials (CONSORT) publishing requirements and to respond to queries from regulatory authorities. Minimal information includes demography, screen failure details, eligibility criteria, any protocol deviations and any SAEs.

Individuals who do not meet the criteria for participation in this study (screen failure) may be rescreened.

5.5. Criteria for Temporarily Delaying

Planned dosing interruptions are permitted in the case of medical/surgical events or logistical reasons not related to study treatment (e.g., elective surgery, unrelated medical events, participant vacation, or holidays). Participants should be placed back on study treatment within 3 weeks of the scheduled interruption, unless otherwise discussed with the Sponsor. The reason for interruption or discontinuation of treatment should be recorded in the electronic case report form (eCRF).

5.6. Withdrawal/Stopping Criteria

Participants will receive study treatment until any of the following occurs:

- Progressive disease (PD) according to RECIST v1.1 **AND*** the participant is clinically unstable, defined as follows:
  - Worsening of performance status
  - Clinically relevant increase in disease-related symptoms
  - Requirement for intensified management of disease-related symptoms (e.g., analgesics, radiation, palliative care)

  ***Note:** PD according to RECIST v1.1 alone is NOT a reason to discontinue study treatment. Clinically stable participants with RECIST v1.1-defined PD should

continue study treatment and a follow-up scan should be performed at least 4 weeks and no longer than 8 weeks later. The follow-up scan will provide additional information to the Investigator for participant management and further treatment decisions. Further guidance for the assessment of scans acquired after RECIST v1.1-defined PD are provided in Appendix 1.

- Unacceptable toxicity or other adverse event, that cannot be managed by dose modification (including meeting stopping criteria for liver chemistry defined in Section 5.6.1)
- Death
- Request of the participant or proxy (withdrawal of consent by subject or proxy)
- Participant is lost to follow-up
- Participant has received PD-1 inhibitor treatment for a maximum of 35 cycles total (approximately 24 months)
- It is in the best interest of the participant as judged by the Investigator and/or Sponsor
- Participant becomes pregnant

In addition, study treatment may be permanently discontinued for any of the following reasons:

- Deviation(s) from the protocol
- Study is closed or terminated

The primary reason study treatment was permanently discontinued must be documented in the participant's medical records and eCRF.

All participants who permanently discontinue study treatment without disease progression will be followed for progression according to the protocol schedule until any of the following occurs:

- Initiation of new anti-cancer therapy
- Disease progression
- Death

All participants who permanently discontinue study treatment will be followed for survival and new anti-cancer therapy, including radiotherapy. If participants are unable or unwilling to attend clinic visits during Follow-up, contact to assess survival may be made via another form of communication (e.g., phone, email, etc.).

Survival follow-up will continue until 80% of the total number of randomized participants have died. At such time, the study will be closed for further follow-up.

If the participant voluntarily discontinues from treatment due to toxicity, 'adverse event (AE)' will be recorded as the primary reason for permanent discontinuation on the eCRF.

Once a participant has permanently discontinued from study treatment, the participant will not be allowed to be retreated.

All participants who discontinue from study treatment will have safety assessments at the time of discontinuation and during post-study treatment follow-up as specified in the SoA (Table 4).

5.6.1. Liver Chemistry Stopping Criteria

Liver chemistry stopping and increased monitoring criteria have been designed to assure participant safety and evaluate liver event etiology.

Discontinuation of study treatment for abnormal liver tests is required when the following occurs:

- A participant meets one of the conditions outlined in the Liver Chemistry Stopping and Increased Monitoring Algorithm (Figure 2)

  OR

- In the presence of abnormal liver chemistry not meeting protocol-specified stopping rules, if the Investigator believes that it is in the best interest of the participant.

Figure 2: Liver Chemistry Stopping and Increased Monitoring Algorithm

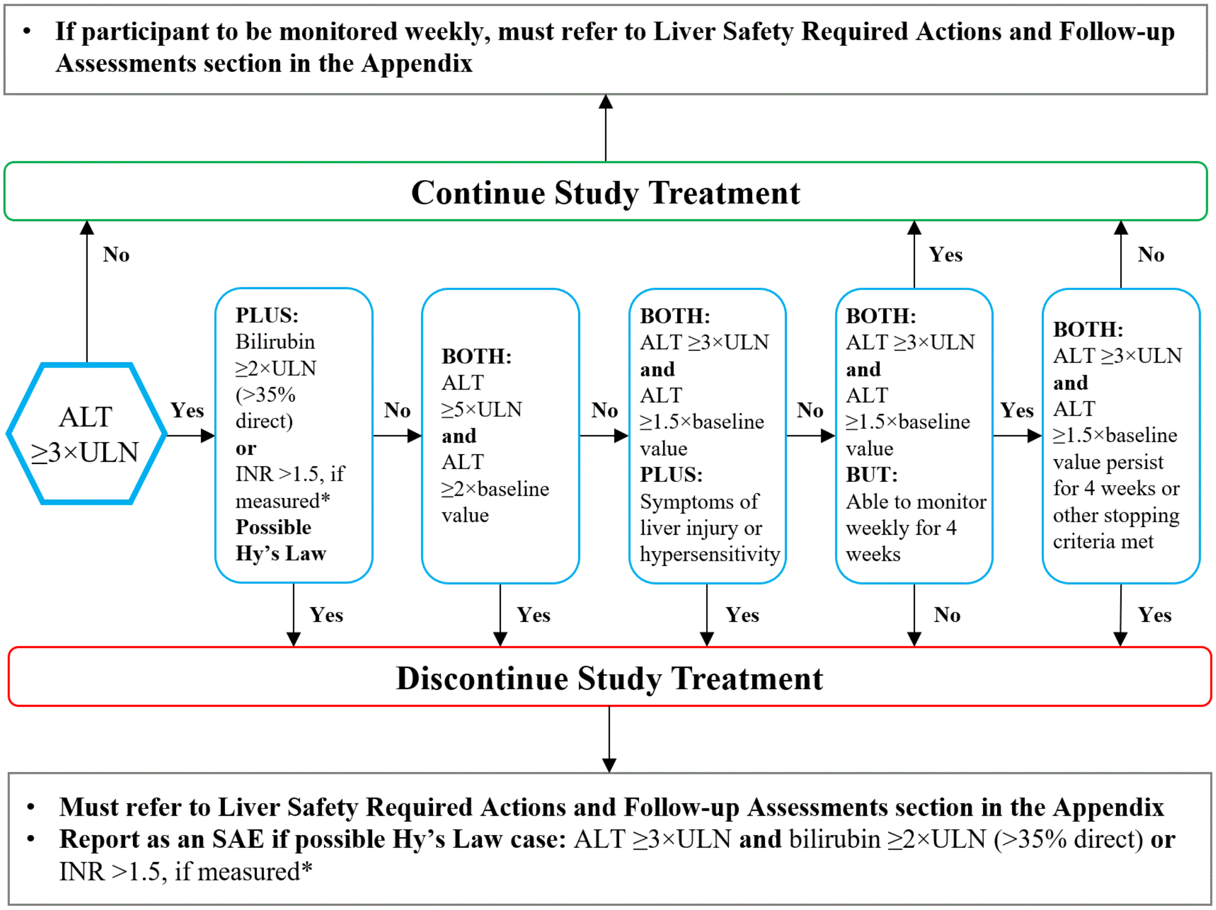

Abbreviations: ALT=alanine aminotransferase; INR=international normalized ratio; SAE=serious adverse event; ULN=upper limit of normal.
*INR value not applicable to participants on anticoagulants.

Liver safety required actions and follow-up assessments can be found in Appendix 6.

5.6.1.1. Restart/Rechallenge After Liver Stopping Criteria Met

If participant meets liver chemistry stopping criteria, do not restart/rechallenge participant with study treatment unless **all** of the following occurs:

- GSK Medical Governance approval **is granted**

  AND
- Ethics and/or IRB approval is obtained, if required

  AND
- Separate consent for intervention restart/rechallenge is signed by the participant

Note: If study treatment was interrupted for suspected drug-induced liver injury, the participant should be informed of the risk of death, liver transplantation, hospitalization, and jaundice and re-consented before resumption of dosing.

- Refer to Appendix 7 for details on the restart/rechallenge process.
- If GSK Medical Governance approval to restart/rechallenge participant with study treatment **is not granted**, then the participant must permanently discontinue study treatment and may continue in the study for protocol-specified follow-up assessments.

5.6.2. QTc Stopping Criteria

If a participant meets the following QTc criteria, they must be discontinued from the study.

- QTc >500msec

  OR
- Change from baseline of QTc >60 msec

For participants with underlying **bundle branch block**, follow the discontinuation criteria in Table 8.

Table 8: Bundle Branch Block QTc Discontinuation Criteria

Baseline QTc with Bundle Branch Block	Discontinuation QTc with Bundle Branch Block
<450 msec	>500 msec
450 to 480 msec	≥530 msec

5.7. Lost to Follow-up

A participant will be considered lost to follow-up if he or she repeatedly fails to return for scheduled visits and is unable to be contacted by the study site.

The following actions must be taken if a participant fails to return to the clinic for a required study visit:

- The site must attempt to contact the participant, reschedule the missed visit as soon as possible, counsel the participant on the importance of maintaining the assigned visit

schedule, and ascertain whether or not the participant wishes to and/or should continue in the study.

- Before a participant is deemed lost to follow-up, the Investigator or designee must make every effort to regain contact with the participant (where possible, 3 telephone calls and, if necessary, a certified letter to the participant's last known mailing address or local equivalent methods). These contact attempts should be documented in the participant's medical record.
- Should the participant continue to be unreachable, he/she will be considered to have withdrawn from the study.
- Site personnel, or an independent third party, will attempt to collect the vital status of the participant within legal and ethical boundaries for all participants randomized, including those who did not get study treatment. Public sources may be searched for vital status information. If vital status is determined as deceased, this will be documented, and the participant will not be considered lost to follow-up. Sponsor personnel will not be involved in any attempts to collect vital status information.

Discontinuation of specific sites or of the study as a whole are outlined in Appendix 4.

5.8. Participant and Study Completion

A participant will be considered to have completed the study as per the end of study definition in Section 4.4, if the participant dies during the Treatment or Follow-up Period, withdraws from the study, or completes the last study related visit or contact, whichever is sooner. If a participant dies, document the cause of death in the eCRF. A participant will be considered to have withdrawn from the study if the participant has not died and is lost to follow up, has withdrawn consent, at the Investigator's discretion is no longer being followed, or if the study is closed/terminated.

6. STUDY TREATMENTS AND CONCOMITANT THERAPY

Study treatment is defined as any investigational treatment(s), marketed product(s), placebo, or medical device(s) intended to be administered to a study participant according to the study protocol.

Study treatments to be administered in this study are summarized in Table 9 and Section 6.1.1, Section 6.1.2, and Section 6.1.3. For further details, refer to the Pharmacy Manual (dostarlimab) or to the product labels.

6.1. Study Treatments Administered

Table 9: Study Treatments for Study 213403

Arm Name	Dostarlimab	Pembrolizumab	Chemotherapy Standard of Care (BOTH Arms)		
**Treatment name**	Dostarlimab	Pembrolizumab	Pemetrexed	Cisplatin	Carboplatin
**Type**	Biologic	Biologic	Drug	Drug	Drug
**Dose formulation**	Solution	Solution	Solution	Solution	Solution
**Unit dose strength**	500 mg Q3W	200 mg Q3W	500 mg/m^2 Q3W	75 mg/m^2 Q3W for 4 cycles	AUC 5 mg/mL/min Q3W for 4 cycles
**Route of administration**	IV infusion	IV infusion	IV infusion	IV infusion	IV infusion
**Use**	Experimental	Comparator	Background intervention	Background intervention	Background intervention
**Sourcing**	Provided centrally by the Sponsor	May be provided centrally by the Sponsor or locally by the study site	May be provided centrally by the Sponsor or locally by the study site	May be provided centrally by the Sponsor or locally by the study site	May be provided centrally by the Sponsor or locally by the study site
**Packaging and labeling**	Study treatment will be provided in a single-use vial. Each vial will be labeled as required per country requirements.	Study treatment will be provided in a single-use vial. Each vial will be labeled as required per country requirements.	Study treatment will be labeled as required per country requirements	Study treatment will be labeled as required per country requirements	Study treatment will be labeled as required per country requirements

Abbreviations: AUC=area under the concentration-time curve; ICF=informed consent form; Q3W=every 3 weeks; SRM=study reference manual.
Note: These formulation details are current at the time of protocol finalization and may be updated in other documents (e.g., SRM and/or ICF) without requiring protocol amendment.

6.1.1. Dostarlimab

Dostarlimab for IV infusion will be supplied by the Sponsor as a solution of 500 mg at a concentration of 50 mg/mL in a single-use vial.

Dostarlimab will be administered through a 30-minute infusion at a dose of 500 mg IV Q3W up to a maximum of 35 cycles total (approximately 24 months).

Sites should make every effort to target the infusion timing to be as close to 30 minutes as possible. Given the variability of infusion pumps from site to site, however, a window between-5 and +15 minutes is permitted.

6.1.2. Pembrolizumab

Pembrolizumab for injection may be supplied by the Sponsor as a solution of 100 mg/4 mL (25 mg/mL) in a single-use vial or may be obtained locally as a commercially available dosage formulation with reimbursement by the Sponsor.

Pembrolizumab will be administered through a 30-minute infusion at a dose of 200 mg Q3W up to a maximum of 35 cycles total (approximately 24 months).

Sites should make every effort to target the infusion timing to be as close to 30 minutes as possible. Given the variability of infusion pumps from site to site, however, a window between-5 and +15 minutes is permitted.

6.1.3. Chemotherapy (Pemetrexed, Cisplatin, and Carboplatin)

Pemetrexed, cisplatin, and carboplatin may be supplied by the Sponsor or may be obtained locally as a commercially available dosage formulation for infusion, with reimbursement by the Sponsor.

Pemetrexed will be administered at 500 mg/m^2 IV through an IV infusion Q3W, up to a maximum of 35 cycles total (approximately 24 months). Pre-treatment for pemetrexed (folic acid, vitamin B12, and glucocorticoids) must be administered according to local guidelines and product label.

Platinum chemotherapy will be administered for the first 4 cycles only, following pemetrexed administration. Chemotherapeutic agents should be administered according to local practice and local approved product labels.

If selected by the Investigator, cisplatin (75 mg/m^2) will be given via IV infusion (approximately 30 minutes after pemetrexed infusion) for the first 4 cycles. For participants receiving cisplatin, pre- and post-treatment hydration procedures must be administered according to local practice and labels.

If selected by the Investigator, carboplatin (area under the concentration-time curve 5 mg/mL/min) will be given via IV infusion immediately following the pemetrexed infusion for the first 4 cycles.

Anti-emetics and hydration may be prescribed per institutional guidance. Refer to local prescribing guidelines for additional information on the dose and instructions for administration of pemetrexed, cisplatin, and carboplatin.

6.1.4. Administration

PD-1 inhibitor (dostarlimab or pembrolizumab) and chemotherapy will be administered at the study site on Day 1 of every 21-day cycle beginning with Cycle 1. After Cycle 1, treatment for subsequent cycles may be administered within a ±3-day window of the scheduled dosing day. Both PD-1 inhibitor and chemotherapy should be given on the same day of the window.

The order of administration is: PD-1 inhibitor (dostarlimab or pembrolizumab) first, immediately followed by pemetrexed, and then followed by cisplatin or carboplatin (Cycles 1 to 4 only). Cisplatin (if selected by the Investigator), should be administered approximately 30 minutes after pemetrexed. Carboplatin (if selected by the Investigator) should be administered immediately following pemetrexed.

6.2. Preparation/Handling/Storage/Accountability

Preparation

The Pharmacy Manual contains specific instructions for the preparation of study treatment and administration of the infusion solution. During the Treatment Period, dostarlimab and pembrolizumab will be prepared by an unblinded pharmacist or designee and dispensed in a blinded manner to the study staff for administration to participants.

Handling and Storage (Including Disposal)

1. The Investigator or designee must confirm appropriate temperature conditions have been maintained during transit for all study treatment received. Any discrepancies must be reported and resolved before use of the study treatment.
2. Only participants enrolled in the study may receive study treatment, and only authorized site staff may supply or administer study treatment. All study treatments must be stored in a secure, environmentally controlled, and monitored (manual or automated) area in accordance with the labeled storage conditions, with access limited to the Investigator and authorized site staff.
3. Precaution will be taken to avoid direct contact with the study treatment. A Safety Data Sheet describing occupational hazards and recommended handling precautions will be provided to the Investigator. In the case of unintentional occupational exposure, notify the Site Monitor, GSK Medical Monitor, and/or GSK study contact.
4. At the end of the study, when all participants have discontinued study treatment, complete drug reconciliation per batch should be available at the site for verification in order to allow drug destruction or return procedure. After receiving Sponsor approval in writing, the investigational site is responsible for destruction of the study treatment according to local regulations. If a site does not have the capability for onsite destruction, the Sponsor will provide a return for destruction service through a third party. Both the unused and expired study treatment must be destroyed, upon authorization of the Sponsor, according to local regulations and procedures, and a copy of the destruction form must be filed in the study binder.

5. The medication provided for this study is to be used only as indicated in this protocol and only for the participants entered in this study.
6. Further guidance and information for the final disposition of unused study treatment are provided in the Study Reference Manual.

Accountability

1. The Investigator, institution, or the head of the medical institution (where applicable) is responsible for study treatment accountability, reconciliation, and record maintenance (i.e., receipt, dispensation, reconciliation, and final disposition records) throughout the study.
2. Details of maintaining drug accountability, including information on the accountability log, will be provided in the Pharmacy Manual.
3. All dispensation and accountability records will be available for Sponsor review. The Study Monitor will assume the responsibility for reconciling the study treatment accountability log. The pharmacist will dispense study treatment for each participant according to the protocol and Pharmacy Manual, if applicable.

6.3. Measures to Minimize Bias

6.3.1. Method of Treatment Assignment: Randomization

Following informed consent and completion of all Screening assessments, participants who meet the inclusion and exclusion criteria will be randomized in a 1:1 ratio to receive either dostarlimab plus chemotherapy or pembrolizumab plus chemotherapy. Randomization will be completed in a blinded manner using an interactive web response system. Randomization will be stratified by PD-L1 status of the tumor (TPS <1% vs 1% to 49% vs ≥50%) and smoking status (never vs former/current).

It is recommended that participants receive the first dose of study treatment on the day of randomization. If Screening assessments were performed within 72 hours of Day 1, repeat testing is not required.

6.3.2. Blinding

Dostarlimab and pembrolizumab must be dispensed in a blinded manner. Details of blinding will be documented in site-specific blinding plans.

In the event of a medical emergency where unblinding is deemed necessary to the care of the participant, the Investigator may have direct access to the participant's treatment code. Detailed instructions for this process are provided in the Blinding Plan and a summary of the general process follows.

Participants and blinded study staff may also be unblinded to study treatment in cases associated with important medical reasons, as determined by the Investigator, and for specific non-urgent medical events. The process for unblinding the identity of the assigned treatment is outlined in the Blinding Plan; refer to the current version of this document for the treatment unblinding process.

If an individual's role on the study requires information about treatment assignment (e.g., an individual is involved in emergency unblinding), procedures will be used to ensure all other personnel remain blinded.

In the event unblinding has occurred, the circumstances necessitating unblinding (i.e., date and details about the situation leading to unblinding) must be documented promptly, and the Unblinded Sponsor Clinical Study Manager notified as soon as possible. Only the Principal Investigator or delegate should be unblinded to the respective participant's code. Study site personnel and Sponsor personnel directly associated with the conduct of the study should not be unblinded.

If the Investigator decides that unblinding is warranted, the Investigator should make every effort to contact GSK prior to unblinding a participant's treatment assignment unless this could delay emergency intervention of the participant. If a participant's intervention assignment is unblinded GSK must be notified within 24 hours after breaking the blind. The date and reason that the blind was broken must be recorded in the source documentation and case report form, as applicable.

GSK's Global Clinical Safety and Pharmacovigilance staff may unblind the treatment assignment for any participant with an SAE. If the SAE requires that an expedited regulatory report be sent to 1 or more regulatory agencies, a copy of the report, identifying the participant's treatment assignment, may be sent to the Investigators in accordance with local regulations and/or GSK policy.

Participants who require unblinding will be discontinued from study treatment, but will remain on study until progression, start of subsequent anticancer treatment, withdrawal of informed consent, loss to follow-up, or death.

6.4. Planned Dose Adjustments

6.4.1. Dose Levels and Dose Adjustment

PD-1 inhibitor (dostarlimab or pembrolizumab) dose reductions are not permitted. Chemotherapy dose reductions are provided in Table 10. Recommended dose modifications for key chemotherapy toxicities are provided in Table 11 and Table 12. These serve as a guide and do not replace Investigator judgment and applicable local label recommendation, if more stringent.

If a dose reduction for toxicity occurs with any agent, the dose may not be re-escalated. Participants can have a maximum of 2 dose modifications (if applicable) to each of the chemotherapy components of study therapy throughout the course of the study for toxicities. If a participant experiences several toxicities and there are conflicting recommendations, the most conservative dose adjustment recommended should be followed (dose reduction appropriate to the most severe toxicity). Participants who require a third dose modification to any particular component will have that agent discontinued.

Reduction of one chemotherapy agent and not the other agent is appropriate if, in the opinion of the Investigator, the toxicity is clearly related to one of the treatments. If, in the opinion of the Investigator, the toxicity is related to the combination of both chemotherapy agents, both drugs should be reduced according to recommended dose

modifications. If the toxicity is related to the combination of three agents, all three agents should be reduced (if applicable), interrupted, or discontinued according to the recommended dose modifications. Participants may have chemotherapy discontinued and continue on PD-1 inhibitor alone. The decision for participants to discontinue PD-1 inhibitor and continue on chemotherapy alone will be made on a case by case basis and the Investigator may consider consultation with the Sponsor for this individualized decision.

Chemotherapy may be interrupted for a maximum of 6 weeks.

Table 10: Dose Modifications for Chemotherapies Used in Study 213403

	**Dose Level 0 (Starting Dose)**	**Dose Level -1**	**Dose Level -2**	**Dose Level -3**
**Pemetrexed**	500 mg/m^2	375 mg/m^2	250 mg/m^2	Discontinue
**Cisplatin**	75 mg/m^2	56 mg/m^2	38 mg/m^2	Discontinue
**Carboplatin**	AUC 5 mg/mL/min (maximum dose: 750 mg)	AUC 3.75 mg/mL/min (maximum dose: 562.5 mg)	AUC 2.5 mg/mL/min (maximum dose: 375 mg)	Discontinue

Abbreviation: AUC=area under the concentration-time curve.

Table 11: Recommended Dose Modifications for Hematological Chemotherapy Toxicities

**Hematologic Events**		**Pemetrexed Dose Level (See Table 10)**	**Cisplatin / Carboplatin Dose Level (See Table 10)**
**Platelets (cells/µL)**	**ANC (cells/µL)**		
≥50,000 **and**	≥500	DL 0	DL 0
≥50,000 **and**	<500	DL -1	DL -1
<50,000 without bleeding **and**	**Any**	DL -1	DL -1
<50,000 with Grade ≥2 bleeding **and**	**Any**	DL -2	DL -2
**Any and**	<1000 **and** fever ≥38.5°C (101°F)	DL -1	DL -1

Abbreviations: ANC=absolute neutrophil count; DL=dose level.

Table 12: Recommended Dose Modifications for Non-hematological Chemotherapy Toxicities

Non-hematologic Events		Pemetrexed Dose Level (See Table 10)	Cisplatin Dose Level (See Table 10)	Carboplatin Dose Level (See Table 10)
**Event**	**NCI-CTCAE Grade**			
Nausea or vomiting	Grade 3 or 4	DL -1	DL -1	DL 0
Diarrhea	Requiring hospitalization irrespective of grade, or Grade 3 or 4	DL -1	DL -1	DL 0
Mucositis	Grade 3 or 4	DL -2	DL 0	DL 0
Neurotoxicity	Grade 2	DL 0	DL -2	DL 0
	Grade 3 or 4	DL -1	Discontinue	DL -1
Transaminase elevation	Grade 3	DL -1	DL -1	DL -1
	Grade 4	Discontinue	Discontinue	Discontinue
Other non-hematological toxicity	Grade 3 or 4	DL -1	DL -1	DL -1

Abbreviations: DL=dose level; NCICTCAE=National Cancer Institute Common Terminology Criteria for Adverse Events.

6.4.2. Guidelines for Events of Special Interest

The severity of AEs will be graded using the NCI-CTCAE v5.0. Guidelines for dose modifications and interruptions for management of common toxicities associated with the study treatment(s) are provided in this section.

6.4.2.1. Management of PD-1 Inhibitor-related AEs

PD-1 inhibitor (dostarlimab or pembrolizumab) treatment may be interrupted or discontinued due to toxicity. AEs (both nonserious and serious) associated with dostarlimab or pembrolizumab exposure may represent an immunologic etiology. These AEs may occur shortly after the first dose or several months after the last dose of study treatment.

PD-1 inhibitor treatment must be withheld for Grade ≥3 toxicities, and certain irAEs, as described in Table 13. The recent joint American Society of Clinical Oncology and National Comprehensive Cancer Network guidelines for the diagnosis and management of irAEs treated with immune checkpoint inhibitor therapy may be used as a supplement to Table 13 (Brahmer, Lacchetti et al. 2018). The medical monitor can be contacted if there are additional questions about PD-1 inhibitor-related AE management. The maximum allowed drug interruption period for PD-1 inhibitors is 12 weeks.

Table 13: Dose Modification Guidelines for PD-1 Inhibitor-related AEs

Immune-related AEs	Toxicity grade or conditions (NCI-CTCAE v5.0)	Action taken with PD-1 inhibitor[1]	irAE management with corticosteroid and/or other therapies	Monitoring and follow-up
**General instructions:**<br>Corticosteroid taper should be initiated upon AE improving to Grade ≤1 and continue to taper over at least 4 weeks.<br>For situations where PD-1 inhibitor has been withheld, treatment can be resumed after AE has been reduced to Grade 1 and corticosteroid has been tapered. PD-1 inhibitor should be permanently discontinued if AE does not resolve within 12 weeks of last dose or corticosteroids cannot be reduced to ≤10 mg prednisone, or equivalent, per day, within 12 weeks.<br>For severe and life-threatening irAEs, IV corticosteroid should be initiated first, followed by oral steroid. Other immunosuppressive treatment should be initiated if irAEs cannot be controlled by corticosteroids.				
Adrenal insufficiency	Grade 2, 3 or 4	Hold until administration of HRT results in return to adequate hormone levels based on laboratory values and restart dosing when toxicity resolves to Grade 1.<br>For recurrent or worsening Grade ≥2 adrenal insufficiency while adequate HRT is continuing, permanently discontinue study treatment.	Start treatment with corticosteroids before other HRT to avoid adrenal crisis (hydrocortisone slowly titrating doses down according to symptoms OR prednisone and fludrocortisone titrating up or down based on BP, other symptoms, and labs); participants with severe symptoms may require additional fluids (e.g., saline >2 L).	Monitor for cortisol level (AM), comprehensive metabolic panel (Na, K, CO_2, glucose) and renin.<br><br>Ensure adequate endocrine evaluation (e.g., Endocrine consultation).

Table 13: Dose Modification Guidelines for PD1 Inhibitor related AEs (Continued)

Immune-related AEs	Toxicity grade or conditions (NCI-CTCAE v5.0)	Action taken with PD-1 inhibitor[1]	irAE management with corticosteroid and/or other therapies	Monitoring and follow-up
AST / ALT elevation or increased bilirubin or hepatitis	Grade 2 with AST or ALT >3 to 5×ULN or total bilirubin >1.5 to 3×ULN	Restart dosing when toxicity resolves to Grade 1	Administer corticosteroids (initial dose of 0.5 to 1 mg/kg methylprednisolone or equivalent) followed by taper	Monitor with liver function tests (consider weekly or more frequently until liver enzyme value(s) return to baseline or are stable.<br>See Section 5.6.1 and Appendix 6 for additional details on Liver Event Follow-up Assessments.
	Grade ≥3 with AST or ALT >5×ULN or total bilirubin >3×ULN	Permanently discontinue[2]	Administer corticosteroids (initial dose of 1 to 2 mg/kg methylprednisolone or equivalent) followed by taper	
Diarrhea / colitis	Grade 2 or 3	Restart dosing when toxicity resolves to Grade 1	Administer corticosteroids (initial dose of 1 to 2 mg/kg methylprednisolone or equivalent) followed by taper	Monitor participants for signs and symptoms of enterocolitis (i.e., diarrhea, abdominal pain, blood or mucus in stool with or without fever) and of bowel perforation (i.e., peritoneal signs and ileus).<br>Participants with Grade ≥2 diarrhea where colitis is suspected should consider GI consultation and an endoscopy to rule out colitis.<br>Participants with diarrhea/colitis should be advised to drink liberal quantities of clear fluids. If sufficient oral fluid intake is not feasible, fluid and electrolytes should be substituted via IV infusion.
	Grade 4 or recurrent Grade 3	Permanently discontinue		

Table 13: Dose Modification Guidelines for PD1 Inhibitor related AEs (Continued)

Immune-related AEs	Toxicity grade or conditions (NCI-CTCAE v5.0)	Action taken with PD-1 inhibitor[1]	irAE management with corticosteroid and/or other therapies	Monitoring and follow-up
Severe neurologic events (myasthenic syndrome/myasthenia gravis, Guillain Barré Syndrome, transverse myelitis)	Grade 2, 3 or 4	Permanently discontinue	Consider high dose corticosteroids and other therapies as needed.<br>It is highly recommended that Investigators discuss any AEs with the Sponsor before using infliximab.	Ensure adequate evaluation (e.g., neurology consultation).<br>Consider MRI of brain and/or spine depending on symptoms.<br>Consider inpatient management as clinically indicated.
Hemophagocytic lymphohistiocytosis	Any grade	Permanently discontinue		
Hyperthyroidism	Grade 3 or 4	Hold until return to adequate hormone levels based on laboratory values and restart dosing when toxicity resolves to Grade 1.	Treat with non-selective beta-blockers (e.g., propranolol) or thionamides as appropriate	Monitor for signs and symptoms of thyroid disorders

Immune-related AEs	Toxicity grade or conditions (NCI-CTCAE v5.0)	Action taken with PD-1 inhibitor[1]	irAE management with corticosteroid and/or other therapies	Monitoring and follow-up
Hypophysitis	Grade 2, 3 or 4	For Grade 2, 3 or 4, hold until administration of HRT results in return to adequate hormone levels based on laboratory values and restart dosing when toxicity resolves to Grade 1. For recurrence or worsening of Grade ≥2 hypophysitis after steroid taper has been completed and patient is on adequate HRT, permanently discontinue.	Administer corticosteroids and initiate HRT as clinically indicated	Monitor for signs and symptoms of hypophysitis (including hypopituitarism and adrenal insufficiency)

Table 13: Dose Modification Guidelines for PD-1 Inhibitor-related AEs (Continued)

Immune-related AEs	Toxicity grade or conditions (NCI-CTCAE v5.0)	Action taken with PD-1 inhibitor[1]	irAE management with corticosteroid and/or other therapies	Monitoring and follow-up
Hypothyroidism	Grade 3 or 4	Hold until administration of HRT results in return to adequate hormone levels based on laboratory values and restart dosing when toxicity resolves to Grade 1.	Initiate thyroid replacement hormones (e.g., levothyroxine or liothyronine) per standard of care	Monitor for signs and symptoms of thyroid disorders.<br>Monitor thyroid function tests.<br>Ensure adequate evaluation (e.g., endocrine consultation).<br>Exclude concomitant adrenal insufficiency (AM cortisol level).

Immune-related AEs	Toxicity grade or conditions (NCI-CTCAE v5.0)	Action taken with PD-1 inhibitor[1]	irAE management with corticosteroid and/or other therapies	Monitoring and follow-up
Immune-related encephalitis	Any grade	Permanently discontinue	Consider IV acyclovir until PCR results obtained.<br>Trial with methylprednisolone; if severe, treatment with methylprednisolone.<br>If positive for autoimmune encephalopathy antibody or no improvement after 7 to 14 days, consider rituximab.	Ensure adequate evaluation to confirm etiology and/or exclude other causes
Infusion-related reaction	See Table 14			
Myocarditis	Grade 2, 3 or 4	Permanently discontinue	Administer high dose corticosteroids (1 g/day of IV methylprednisolone) for 3 to 5 days, followed by oral prednisone taper over 4 to 6 weeks based on improvement in cardiac function and biomarkers.<br>If no improvement in 24 hours, consider adding other potent immunosuppressive agents.	Ensure adequate evaluation (e.g., Urgent cardiology consultation) to confirm etiology and/or exclude other causes

Table 13: Dose Modification Guidelines for PD-1 Inhibitor-related AEs (Continued)

Immune-related AEs	Toxicity grade or conditions (NCI-CTCAE v5.0)	Action taken with PD-1 inhibitor[1]	irAE management with corticosteroid and/or other therapies	Monitoring and follow-up
Pneumonitis	Grade 2	Restart dosing when toxicity resolves to Grade 1. If Grade 2 recurs, permanently discontinue.	Administer corticosteroids (initial dose of 1 to 2 mg/kg methylprednisolone or equivalent) followed by taper	Monitor participants for signs and symptoms of pneumonitis.<br>Evaluate participants with suspected pneumonitis with radiographic imaging and initiate corticosteroid treatment.<br>Add prophylactic antibiotics for opportunistic infections.
	Grade 3 or 4, or recurrent Grade 2	Permanently discontinue		
Rash / skin reactions	Grade 3 or suspected DRESS, SJS or TEN	Withhold	Treat with high potency topical steroids to affected areas.<br>Treat with prednisone.	Ensure adequate evaluation (e.g., urgent dermatology consultation) to confirm etiology and/or exclude other causes
	Grade 4 or confirmed DRESS, SJS or TEN	Permanently discontinue	Administer 1 to 2 mg/kg/day IV methylprednisolone and taper steroid when dermatitis is controlled.	
Recurrence of AEs after resolution to Grade ≤1	Grade 1 or 2	Withhold	Based on severity of AEs, administer appropriate treatment until symptoms improve to Grade ≤2.	
	Grade 3 or 4	Permanently discontinue		

Table 13: Dose Modification Guidelines for PD-1 Inhibitor-related AEs (Continued)

Immune-related AEs	Toxicity grade or conditions (NCI-CTCAE v5.0)	Action taken with PD-1 inhibitor[1]	irAE management with corticosteroid and/or other therapies	Monitoring and follow-up
Renal failure or nephritis	Grade 2 with creatinine >1.5 to ≤3×ULN	Restart dosing when toxicity resolves to Grade 1.	Start treatment with prednisone; if persistent Grade 2 beyond 1 week, prednisone/methylprednisolone.	Monitor participants for signs and symptoms, including monitoring of creatinine and urine protein every 3 to 7 days.<br>Ensure adequate evaluation (e.g., nephrology consultation) to confirm etiology and/or exclude other causes.
	Grade ≥3 with creatinine >3×ULN	Permanently discontinue	Start treatment with prednisone; if persistent Grade 2 beyond 1 week, prednisone/methylprednisolone.<br>Consider adding one of the following after 1 week of steroids: azathioprine, cyclosporine, cyclophosphamide, infliximab, mycophenolate.	Ensure adequate evaluation (e.g., nephrology consultation, renal biopsy) to confirm etiology and/or exclude other causes.<br>Consider inpatient care.
Type 1 diabetes mellitus (T1DM) or hyperglycemia	Grade 3 to 4 hyperglycemia or T1DM (associated with metabolic acidosis or ketonuria)	Restart dosing in appropriately managed clinically and metabolically stable participants; insulin replacement therapy is required	Initiate insulin replacement therapy for participants with T1DM.<br>Administer anti-hyperglycemic in participants with hyperglycemia.	Monitor participants for hyperglycemia or other signs and symptoms of diabetes
Uveitis	Grade ≥2	Withhold	Urgent ophthalmology consultation.<br>Administer treatment with ophthalmic and systemic prednisone/methylprednisolone.	Ensure adequate evaluation (e.g., urgent ophthalmology consultation)

Table 13: Dose Modification Guidelines for PD-1 Inhibitor-related AEs (Continued)

Immune-related AEs	Toxicity grade or conditions (NCI-CTCAE v5.0)	Action taken with PD-1 inhibitor[1]	irAE management with corticosteroid and/or other therapies	Monitoring and follow-up
Other irARs	Based on severity and type of reaction (Grade 2 or 3)	Restart dosing when toxicity resolves to Grade 1	Based on severity of AE, administer corticosteroids. When controlled, taper steroid.	Ensure adequate evaluation to confirm etiology and exclude other causes.
	Grade 4 or recurrent Grade 3	Permanently discontinue		

Abbreviations: AE=adverse event; ALT=alanine aminotransferase; AST=aspartate aminotransferase; DRESS= drug reaction with eosinophilia and systemic symptoms; HRT=hormone replacement therapy; irAR=immune related adverse reaction; PD-1=programmed cell death protein 1; SJS=Stevens Johnson syndrome; T1DM=Type 1 diabetes mellitus; TEN=toxic epidermal necrolysis.

1. This table is reflective of pembrolizumab Summary of Product Characteristics guidance.
2. For participants with liver metastasis who begin treatment with Grade 2 AST or ALT, if AST or ALT increases by ≥50% relative to baseline and lasts for at least 1 week, then the participant should be discontinued.

6.4.2.2. Management of Chemotherapy-related AEs

Hematological and nonhematological AEs attributable to pemetrexed, carboplatin, and cisplatin should be managed according to guidelines in the prescribing information and by local institutional practice.

6.4.2.3. Management of Infusion-related Reactions

Participants receiving IV study treatment may develop signs and symptoms of an infusion reaction during or shortly after drug infusion. These reactions generally resolve completely within 24 hours of completion of infusion. Table 14 shows treatment guidelines for participants who experience infusion-related reactions associated with administration of study treatment, including actions to be taken with the study treatment, if necessary.

Table 14: Infusion Reaction Treatment Guidelines

NCI-CTCAE Grade	Treatment	Premedication at Subsequent Dosing
**Grade 1**<br>Mild reaction; infusion interruption not indicated; treatment not indicated	Increase monitoring of vital signs as medically indicated until the participant is deemed medically stable in the opinion of the Investigator.	None
**Grade 2**<br>Requires infusion interruption but responds promptly to symptomatic treatment (e.g., antihistamines, NSAIDs, narcotics, or IV fluids); prophylactic medications indicated for ≤24 hours	Stop infusion and monitor symptoms.<br>Additional appropriate medical therapy may include, but is not limited to the following:<br>• IV fluids<br>• Antihistamines<br>• NSAIDs<br>• Acetaminophen<br>• Narcotics<br>Increase monitoring of vital signs as medically indicated until the participant is deemed medically stable in the opinion of the Investigator.<br>If symptoms resolve within 1 hour of stopping drug infusion, the infusion may be restarted at 50% of the original infusion rate (e.g., from 100 mL/h to 50 mL/h). Otherwise, dosing will be withheld until symptoms resolve, and the participant should be premedicated for the next scheduled dose.<br>**Participants who develop Grade 2 toxicity despite adequate premedication should be permanently discontinued from further study treatment administration**.	Participant may be premedicated 1.5 hours (± 30 minutes) prior to infusion of dostarlimab or pembrolizumab with the following:<br>• Diphenhydramine 50 mg PO (or equivalent dose of antihistamine)<br>• Acetaminophen 500 to 1,000 mg PO (or equivalent dose of antipyretic)
**Grade 3**:<br>Prolonged (i.e., not rapidly responsive to symptomatic medication and/or brief interruption of infusion); recurrence of symptoms following initial improvement; hospitalization indicated for other clinical sequelae (e.g., renal impairment, pulmonary infiltrates)<br>**Grade 4**:<br>Life-threatening; pressor or ventilatory support indicated	**Stop Infusion.**<br>Additional appropriate medical therapy may include, but is not limited to the following:<br>• IV fluids<br>• Antihistamines<br>• NSAIDs<br>• Acetaminophen<br>• Narcotics<br>• Oxygen<br>• Pressors<br>• Corticosteroids<br>• Epinephrine<br>Increase monitoring of vital signs as medically indicated until the participant is deemed medically stable in the opinion of the Investigator.<br>Hospitalization may be indicated.<br>**Participant is permanently discontinued from further study treatment administration.**	No subsequent dosing.

Abbreviations: IV=intravenous; NCI-CTCAE=National Cancer Institute-Common Terminology Criteria for Adverse Events; NSAID=nonsteroidal anti-inflammatory drug; PO=orally.
Note: Appropriate resuscitation equipment should be available in the room and a physician is readily available during the period of study treatment administration.

6.5. Participant-specific Dose Adjustments

There are no participant-specific dose adjustments defined for this protocol.

6.6. Study Treatment Compliance

When participants are dosed at the site, they will receive study treatment directly from the Investigator or designee, under medical supervision. The date and time of each dose administered in the clinic will be recorded in the source documents. The dose of study treatment and study participant identification will be confirmed at the time of dosing by a member of the study site staff other than the person administering the study treatment.

6.7. Treatment of Overdose

6.7.1. Protocol-defined Overdose

For this study, an overdose of dostarlimab is defined as any dose that is ≥20% of 500 mg Q3W. No specific information is available on the treatment of overdose of dostarlimab. In the event of overdose, the participant should be observed closely for signs of toxicity. Appropriate supportive treatment should be provided if clinically indicated.

An overdose of pembrolizumab is defined, for this study, as a dose ≥1,000 mg (5 × the dose). No specific information is available on the treatment of overdose of pembrolizumab. In the event of overdose, the participant should be observed closely for signs of toxicity. Appropriate supportive treatment should be provided, if clinically indicated.

An overdose of pemetrexed, cisplatin, or carboplatin is defined according to the respective prescribing information.

In the event of an overdose, the Investigator should do the following:

1. Contact the GSK Medical Monitor immediately.
2. Closely monitor the participant for AE/SAE and laboratory abnormalities until study treatment of interest can no longer be detected systemically (at least 130 days).
3. Document the quantity of the excess dose as well as the duration of the overdosing in the eCRF.

Decisions regarding dose interruptions or modifications will be made by the Investigator in consultation with the GSK Medical Monitor based on the clinical evaluation of the participant.

See Section 7.5.4 for details on reporting a study treatment overdose.

6.8. Treatment After the End of the Study

Post-study treatment will not be provided as part of the protocol. Upon discontinuation from their assigned study treatment, participants may receive additional (non-protocol) therapy at the discretion of the treating physician. New therapy should be documented in the eCRF. Every effort should be made to complete the required withdrawal and

follow-up evaluations prior to initiating further therapy or dosing of an investigational agent (see Table 4 for follow-up assessments and procedures).

6.9. Concomitant Medications and Non-drug Therapies

Participants should receive full supportive care during the study, including transfusion of blood and blood products, and treatment with antibiotics, antiemetics, antidiarrheals, and analgesics, as appropriate.

Participants will be instructed to inform the Investigator prior to starting any new medications from the time of first dose of study treatment until the end of the study (Final Study Visit). Any concomitant medication(s), including non-prescription medication(s) and herbal product(s), taken during the study will be recorded in the eCRF. The minimum requirement is that drug name, dose, and the dates of administration be recorded. Additionally, a complete list of all prior anti-cancer therapies will be recorded in the eCRF.

Questions regarding concomitant medications should be directed to the GSK Medical Monitor for clarification.

If future changes are made to the list of permitted/prohibited medications, formal documentation will be provided by GSK and stored in the study file. Any such changes will be communicated to the investigative sites in the form of a letter.

6.9.1. Prohibited Medications and Non-drug Therapies

Participants are prohibited from using the following medications and non-drug therapies:

- Systemic anticancer medicines
- Biological therapy, immunotherapy, or chemotherapy not specified in the protocol
- Investigational agents
- Live virus and bacterial vaccines
- Systemic glucocorticoids other than those used to manage AEs or as pre-medications to study therapy
- Participants taking NSAIDs or salicylates will not take the NSAID or salicylate (other than an aspirin dose ≤1.3 grams per day) for 2 days before, the day of, and 2 days after receiving pemetrexed. Participants taking NSAIDs or salicylates with a long half-life (e.g., naproxen, piroxicam, diflunisal, or nabumetone) will not take the NSAIDs or salicylates for 5 days before, the day of, and 2 days after pemetrexed.

The study treatment product labels must be consulted to determine any additional prohibited medications.

7. STUDY ASSESSMENTS AND PROCEDURES

7.1. General Guidelines

- Study procedures and their timing are summarized in the SoA (Table 4).
- Protocol waivers or exemptions are not allowed.
- Immediate safety concerns should be discussed with the Sponsor immediately upon occurrence or awareness to determine if the participant should continue or discontinue study treatment.
- Adherence to the study design requirements, including those specified in the SoA, is essential and required for study conduct.
- All Screening evaluations must be completed and reviewed to confirm that potential participants meet all eligibility criteria. The Investigator will maintain a screening log to record details of all participants screened and to confirm eligibility or record reasons for screening failure, as applicable.
- Procedures conducted as part of the participant's routine clinical management (e.g., blood count) and obtained before signing of the ICF may be utilized for Screening or baseline purposes, provided the procedure met the protocol-specified criteria and was performed within the time frame defined in the SoA.
- Results that could unblind the study will not be reported to investigative sites or other blinded personnel until the study has been unblinded.
- Repeat or unscheduled samples may be taken for safety reasons or for technical issues with the samples.

7.1.1. General Guidance for Treatment Continuity when Participants are Unable to Come into the Clinic

Due to the significant challenges that currently face the healthcare system and patients due to Coronavirus disease 2019 (COVID-19), as well as the potential for enduring or additional quarantine measures, the following guidance is being provided in this protocol. In the spirit of global diversity in the COVID-19 pandemic and its impact on healthcare in each individual country as well as the recently issued guidance by several regulatory authorities, the autonomy of each investigative site to assess the benefit/risk for their patients participating in clinical studies should be maintained.

Prior to utilization of any of the measures outlined in this section, discussion and approval must be obtained from Sponsor/contract research organization.

It is expected that sites participating in clinical studies will make every effort to ensure proper monitoring and well-being of enrolled participants by adhering to safety monitoring as outlined in the SoA (Table 4). The use of local laboratories and local radiology centers to reduce the need for a participant to come into the clinic are supported, if deemed necessary for the well-being of the participant. These local facilities should be added to regulatory documents, as required.

Additionally, regulatory guidance issued in response to the COVID-19 pandemic supports the use of central and remote monitoring programs to maintain oversight of

clinical sites. Any restrictions in place at the site that will impact monitoring and/or participant access to the site and care providers should be communicated to the Sponsor/contract research organization.

A global telemedicine platform that allows for continued monitoring of AEs, concomitant medications, protocol deviations, etc, may be engaged. Discussions around utilization of this technology should be held on a per-site basis and appropriate documentation of utilization should be captured.

General rules for participants with limited possibility to travel:

- If possible, replace in-person visits with phone contact or alternative location for assessment (e.g., local laboratories and imaging centers).
- In instances where it is desired to reduce participant exposure in clinic, in-person visits every other visit are acceptable if there are no ongoing AEs or new AEs. At this time, these missed visits will be considered protocol deviations.

Table 15: Critical Data Collection and Safety Precautions

Assessment	Recommendation
Follow-up assessments	Contact participant by phone. This discussion should include assessment of new therapies and overall survival.
PROs (Treatment Period and Follow-up)	May be completed by the participant using their own digital device.
CBC	Local laboratory, if possible. Arrangements for the use of a local laboratory should be made by the site including the reporting of results to the Investigator for review.
Serum chemistry	Local laboratory, if possible. Arrangements for the use of a local laboratory should be made by the site including the reporting of results to the Investigator for review.
AEs	Ongoing AEs and SAEs reviewed by phone.<br>If hematologic AEs are ongoing, a local CBC is desirable.<br>New AEs/SAEs- may be assessed by phone; (please remember to submit SAE documentation within 24 hours of learning of the event).
Concomitant medications	Reviewed by phone and via medical record review.
Evaluation of response	CT/MRI if possible.
PD	CT/MRI if possible.
Pregnancy test	Local laboratory, if available. Arrangements for the use of a local laboratory should be made by the site including the reporting of results to the Investigator for review.<br>Per ICF participant must avoid getting pregnant.

Abbreviations: AE-adverse event; CBC=complete blood count; CT=computed tomography; ICF=informed consent form; MRI=magnetic resonance imaging; PD=progressive disease; PRO=patient-reported outcome; SAE=serious adverse event.

7.2. Screening and Critical Baseline Assessments

7.2.1. Baseline Assessments at Screening

Refer to the SoA (Table 4) for the full list of assessments collected during the Screening Period of this study.

7.2.2. Baseline Tumor Assessment (Documentation of Target and Non-target Lesions)

- All baseline lesion assessments must be performed within 35 days prior to the first dose of study treatment.
- If a participant had a tumor assessment of the clinically indicated areas within the 35 days before dosing on Cycle 1/Day 1, but prior to signing the main ICF, an additional CT/MRI scan for study screening is not required. CT/MRI scans completed during

Screening prior to signing the main ICF must have been performed and available for submission per the image acquisition guidelines.

- Lymph nodes that have a short axis of <10 mm are considered non-pathological and should not be recorded or followed.
- Pathological lymph nodes with <15 mm and but ≥10 mm short axis are considered non-measurable.
- Pathological lymph nodes with ≥15 mm short axis are considered measurable and can be selected as target lesions, however, lymph nodes should not be selected as target lesions when other suitable target lesions are available.
- Measurable lesions up to a maximum of 2 lesions per organ and 5 lesions in total, representative of all involved organs, should be identified as target lesions, and recorded and measured at baseline. These lesions should be selected on the basis of their size (lesions with the longest diameter) and their suitability for accurate repeated measurements (either by imaging techniques or clinically).

  Note: Cystic lesions thought to represent cystic metastases should not be selected as target lesions when other suitable target lesions are available.

  Note: Measurable lesions that have been previously irradiated and have not been shown to be progressing following irradiation should not be considered as target lesions.
- Lytic bone lesions or mixed lytic-blastic lesions, with identifiable soft tissue components, that can be evaluated by CT or MRI can be considered measurable. Bone scans, fluorodeoxyglucose-positron emission tomography (FDG-PET) scans, or X-rays are not considered adequate imaging techniques to measure bone lesions.
- All other lesions (or sites of disease) should be identified as non-target and should also be recorded at baseline. Non-target lesions will be grouped by organ. Measurements of these lesions are not required, but the presence or absence of each should be noted throughout Follow-up.
- Within 35 days prior to the first dose, participants must have a baseline tumor assessment of the chest and abdomen (including the entire liver and both adrenal glands). Other regions should be imaged as clinically indicated (e.g., pelvis, brain, etc). This assessment should be conducted by IV contrast-enhanced computed tomography (CT) or magnetic resonance imaging (MRI) scan. MRI should be used for abdominal (and pelvic) imaging if CT contrast is contraindicated, and, preferably, for IV contrast-enhanced imaging of the brain. The same imaging technique should be used in a participant throughout the study. The CT component of positron emission tomography (PET)/CT may be used according to RECIST v1.1 guidelines, with full radiation dose diagnostic CT and IV CT contrast, and as clinically indicated. At each post-baseline assessment, evaluations of the sites of the disease identified by these scans are required.

7.2.3. Determination of PD-L1 Status Prior to Randomization

To be eligible for the study, participants must have documented PD-L1 status by the 22C3 pharmDx assay (Agilent/Dako). If no prior PD-L1 result is available at the time of

Screening, the participant can be tested locally using the aforementioned method, or central PD-L1 testing can be completed (refer to the Laboratory Manual for country-specific details of central assessment). For participants requiring PD-L1 testing at the time of study entry, an archival FFPE tumor tissue specimen, which may have been collected at any time prior to Screening, can be submitted for analysis within 28 days of the participant's first dose. Specimens used to confirm diagnosis or collected after the participant has been diagnosed with metastatic disease will be preferred for determination of PD-L1 status. Both tissue block and freshly cut (within 6 months) slides (10 to 15) are acceptable. If no archival FFPE tumor tissue is available, a fresh tumor tissue biopsy should be obtained and tested within 28 days of the participant's first dose. Biopsies obtained prior to receipt of adjuvant/neoadjuvant chemotherapy will be permitted if a fresh biopsy is not feasible. As the results of baseline PD-L1 status assessment will be used as a stratification factor in this study, results must be provided prior to randomization. Participants who do not have documented PD-L1 status and cannot provide archived or fresh tissue sample for PD-L1 testing will not be eligible for the study.

7.3. Efficacy

7.3.1. Post-baseline Imaging and Tumor Assessment

- The efficacy of each treatment combination will be evaluated by assessment of tumor response according to RECIST v.1.1 (Appendix 1) by BICR and by the Investigator. Investigator assessment of tumor response and progression according to RECIST v1.1 will be used for treatment decisions.
- Tumor assessment method and timing, evaluation of disease, disease progression, and response criteria will be conducted according to RECIST v1.1 as follows:
  - Tumor assessment modalities may include imaging (e.g., CT or MRI).

To support continued tumor assessment while on study, serial imaging will then be performed at 6 weeks (42 [±7] days) and again at 12 weeks (84 [±7] days) from the randomization date. Following the 12-week scan, imaging will be performed Q9W (every 63 [±7] days), or more frequently, if clinically indicated. After 48 weeks, participants who remain on treatment will have imaging performed Q12W (every 84 [±7] days). Imaging should continue to be performed until discontinuation of study treatment due to disease progression with clinical instability, start of subsequent anticancer treatment, withdrawal of informed consent, or death, whichever comes first. Imaging must be performed on a calendar schedule and should not be affected by dose interruptions/delays. All radiographic images/scans at the specified time points, as well as any unscheduled images/scans, will be collected and stored centrally for BICR, potential future evaluation, and archiving. Digital copies of all DICOM-formatted scans must be maintained at the Investigative site as source documents.

- For post-baseline assessments, a window of ±7 days is permitted to allow for flexible scheduling.

- To ensure comparability between the baseline and subsequent assessments, the same method of assessment and the same imaging technique will be used when assessing response.
- Following the initial radiologic assessment of progressive disease (PD), clinically stable participants should continue study treatment and a follow-up scan should be performed at least 4 weeks and no longer than 8 weeks later. The follow-up scan will provide additional information to the Investigator for participant management and further treatment decisions. Further guidance for the assessment of scans acquired after RECIST v1.1-defined PD are provided in Appendix 1. For participants who are not clinically stable (clinically progressing) at the initial radiologic assessment of PD, study treatment will be discontinued and a follow-up scan is not required. Following the initial radiologic assessment of CR or PR, a confirmatory follow-up scan should be performed at least 4 weeks later.
- If EOT occurs between the scheduled radiologic disease assessments, scans do not need to be repeated at EOT if fewer than 5 weeks have passed since the time of the previous disease assessment that did not document PD. To ensure comparability between the baseline and subsequent assessments, the same method of assessment and the same imaging technique will be used when assessing response.

7.3.1.1. Objective Response Rate

ORR as a primary efficacy endpoint in this study will be evaluated by RECIST v1.1 based on BICR and will be defined as the proportion of participants with a best overall response (BOR) of confirmed CR or PR in the analysis population.

CCI [redacted]

7.3.1.2. Overall Survival

OS as a secondary efficacy endpoint in this study will be defined as the time from the date of randomization to the date of death by any cause. Following the EOT Visit, survival status will be collected for all participants using acceptable means, including telephone contact. Participants without documented death at the time of the final analysis will be censored at the last date they were known to be alive.

7.3.1.3. Progression-free Survival

PFS as a secondary efficacy endpoint in this study will be evaluated using RECIST v1.1 based on Investigator assessment and will be defined as the time from the date of randomization to the date of PD or death by any cause, whichever occurs first.

7.3.1.4. Duration of Response

CCI [redacted]

7.4. Safety

Refer to the SoA (Table 4) for full list of safety assessments and their frequency and timing during this study.

7.4.1. Physical Examinations

- A full physical examination will include assessments of the cardiovascular, respiratory, gastrointestinal, and neurological systems. Height (at Screening only) and weight will also be measured and recorded. Full physical examinations will be conducted during Screening and at EOT or discontinuation.

  Note: For height and weight measurements, the participant is allowed to wear indoor, daytime clothing with no shoes.
- Symptom-directed physical examinations will include assessments based on participants' current symptoms and may include brief examination of the lungs, cardiovascular system, and abdomen (liver and spleen).
- Investigators should pay special attention to clinical signs related to previous serious illnesses.

7.4.2. ECOG Performance Status

- Performance status will be assessed using the ECOG performance status scale (Appendix 2). The same observer should assess performance status each time, if possible.

7.4.3. Vital Signs

- Vital sign measurements are to be measured in a semi-supine position after a 5-minute rest and will include temperature, systolic and diastolic blood pressure, pulse rate, and respiratory rate.
- Three readings of blood pressure and pulse rate should be taken. The first reading should be rejected and the second and third readings should be averaged to give the measurement to be recorded in the eCRF.
- Vital signs will be measured more frequently if warranted by clinical condition of the participant. On days where vital signs are measured multiple times, temperature does not need to be repeated, unless clinically indicated.

7.4.4. Pulse Oximetry

- Pulse oximetry will be performed using local standard procedures. This assessment should be completed prior to study treatment administration when performed during the Treatment Period.

7.4.5. Electrocardiograms

- All participants will undergo electrocardiograms (ECGs). Participants will be in a supine or semirecumbent position (about 30 degrees of elevation) and rested for approximately 2 minutes before ECGs are recorded.

7.4.6. Clinical Safety Laboratory Assessments

- See Appendix 3 for the full list of clinical laboratory tests to be performed and to the SoA (Table 4) for the timing and frequency of these tests. Clinical laboratory assessments will be performed by the local laboratory at the investigational site.
- The Investigator must review the laboratory report, document this review, and record any clinically significant changes occurring during the study as an AE. The laboratory reports must be filed with the source documents. If SAE criteria are met or the abnormality is an adverse event of special interest (AESI) (Appendix 8), the event should be recorded and reported according to the SAE reporting process (Section 7.5.4).
- Abnormal laboratory findings associated with the underlying disease are not considered clinically significant, unless judged by the Investigator to be more severe than expected for the participant's condition.
- All laboratory tests with values considered clinically significantly abnormal during participation in the study, or within 90 days after the last dose of study treatment, should be repeated until the values return to normal/baseline or are no longer considered significantly abnormal by the Investigator or GSK Medical Monitor.
  - If clinically significant values do not return to normal/baseline within a period of time judged reasonable by the Investigator, the etiology should be identified, and the Sponsor should be notified.
- Hematologic testing may occur more frequently than is specified in the SoA if additional testing is medically indicated per the Investigator's judgment or if an event meeting the criteria for study dose adjustment occurs (Section 6.4).
- All protocol-required laboratory tests must be conducted in accordance with the Laboratory Manual and the SoA (Table 4).
- Additional tests may be performed at a laboratory facility other than the study site, but the test results must be reported to the study site, the study site must keep a copy of the test results with the participant's study file, as well as laboratory reference ranges for the facility used, and the results must be entered into the eCRF.
- If laboratory values from non-protocol specified laboratory tests performed at the institution's local laboratory require a change in participant management or are considered clinically significant by the Investigator (e.g., AE, SAE, or dose modification), then the results must be recorded.
- Further details on sample collection and analysis can be found in the Laboratory Manual. Any suspected case of secondary cancer reported while a participant is receiving treatment or followed for post-treatment assessments must be investigated, including obtaining and documenting a histological diagnosis. Testing completed as part of standard of care is sufficient as long as the methods are deemed acceptable after consultation with the GSK Medical Monitor.

7.4.7. Pregnancy Testing

- Refer to Section 5.1 for pregnancy testing study entry criteria.
- Pregnancy testing (urine or serum as required by local regulations) should be conducted prior to each dose of study treatment (within 72 hours).
- Monthly pregnancy testing (urine or serum as required by local regulations) should be conducted at the end of relevant systemic exposure to the study treatment, plus at least 180 days, minimum, thereafter (or longer as per the required contraception use in this study; see Section 5.1).
- Additional serum or urine pregnancy tests may be performed, as determined necessary, by the Investigator or required by local regulation, to establish the absence of pregnancy at any time during the participant's participation in the study.
- Any pregnancies that occur while a participant is on study (inclusive of the minimum 180 days post treatment follow-up period or longer as per the required contraception use in this study) are to be reported as described in Section 7.5.5.

7.5. AEs, SAEs, and Other Safety Reporting

The definitions of AEs, SAEs, and other safety events, including AESIs can be found in Appendix 8.

AEs will be reported by the participant (or, when appropriate, by a caregiver, surrogate, or the participant's legally authorized representative).

The Investigator and any qualified designees are responsible for detecting, documenting, and reporting events that meet the definition of an AE or SAE and remain responsible for following-up all events.

The method of recording, evaluating, and assessing causality of AEs and SAEs and the procedures for completing and transmitting SAE reports are provided in Appendix 8.

7.5.1. Time Period and Frequency for Collecting AE and SAE Information

- All AEs and SAEs will be collected from the start of study treatment until the time points outlined in Table 16 and the SoA (Table 4). However, any SAEs assessed as related to study participation (e.g., study treatment, protocol-mandated procedures, invasive tests, or change in existing therapy) or related to a GSK product will be recorded from the time a participant consents to participate in the study.

Table 16: Collecting and Reporting AEs, Pregnancy, and Survival

Parameter	Time Point
AEs	Through 30 days after cessation of study treatment
SAEs	Through 90 days after cessation of study treatment (or for a minimum of 30 days until the start of alternate anticancer therapy)
Pregnancy	Through at least 180 days following the last dose of study treatment OR as per local requirements and local approved product labels (applies to female participants and female partners of male participants; see Section 5.1 for additional details)
Study drug-related SAEs	Until study closeout
AESIs	Until study closeout
Survival	Until study closeout

Abbreviations: AE=adverse event; AESI=adverse event of special interest; SAE=serious adverse event.
Note: Collection and reporting will begin on the day of informed consent.

- Medical occurrences that begin before the start of study treatment, but after obtaining informed consent, will be recorded as medical history/current medical conditions, not as AEs.
- All SAEs will be recorded and reported to the Sponsor or designee immediately, and, under no circumstance, should this exceed 24 hours, as indicated in Appendix 8. The Investigator will submit any updated SAE data to the Sponsor within 24 hours of it being available.
- Investigators are not obligated to actively seek information on AEs or SAEs after the conclusion of a participant's study participation. However, if the Investigator learns of any SAE, including a death, at any time after a participant has been discharged from the study, and he/she considers the event to be reasonably related to the study treatment or study participation, the Investigator must promptly notify the Sponsor.

7.5.2. Method of Detecting AEs and SAEs

Care will be taken not to introduce bias when detecting AEs and SAEs. Open-ended and non-leading verbal questioning of the participant is the preferred method to inquire about AE occurrence.

7.5.3. Follow-up of AEs and SAEs

After the initial AE/SAE report, the Investigator is required to proactively follow each participant at subsequent visits/contacts. All SAEs and AESIs will be followed until the event is resolved, stabilized, or otherwise explained, or the participant is lost to follow-up (as defined in Section 5.7). Further information on follow-up procedures is given in Appendix 8.

7.5.4. Regulatory Reporting Requirements for SAEs

- Prompt notification by the Investigator to the Sponsor of an SAE is essential so that legal obligations and ethical responsibilities toward the safety of participants and the safety of a study treatment under clinical investigation are met.
- The Sponsor has a legal responsibility to notify both the local regulatory authority and other regulatory agencies about the safety of a study treatment under clinical investigation. The Sponsor will comply with country-specific regulatory requirements relating to safety reporting to the regulatory authority, Institutional Review Boards (IRBs)/Independent Ethics Committees (IECs), and Investigators.
- An Investigator who receives an Investigator Safety Report describing an SAE or other specific safety information (e.g., summary or listing of SAEs) from the Sponsor will review and then file it along with the IB, and will notify the IRB/IEC, if appropriate, according to local requirements.
- Investigator Safety Reports must be prepared for suspected unexpected serious adverse reactions (SUSARs) according to local regulatory requirements and Sponsor policy and forwarded to Investigators, as necessary.

7.5.5. Pregnancy

- Details of all pregnancies in female participants and female partners of male participants will be collected after the start of study treatment and until at least 180 days following the last dose of study treatment (or longer, per the time period for post-treatment contraception requirements in Section 5.1).
- If a pregnancy is reported, the Investigator will record pregnancy information on the appropriate form and submit it to GSK within 24 hours of learning of the pregnancy of the female participant or female partner of a male participant (after obtaining the necessary signed informed consent from the female partner). Although pregnancy itself is not considered to be an AE or SAE, any pregnancy complication or elective termination of a pregnancy for medical reasons will be reported as an AE or SAE.
- Abnormal pregnancy outcomes (e.g., spontaneous abortion, fetal death, stillbirth, congenital anomalies, ectopic pregnancy) are considered SAEs and will be reported as such.
- The pregnant female participant or pregnant female partner of a male participant will be followed to determine the outcome of the pregnancy. The Investigator will collect follow-up information on the pregnant female participant or pregnant female partner of a male participant and the neonate and the information will be forwarded to the Sponsor.
- Any post-study pregnancy-related SAE considered reasonably related to the study treatment by the Investigator will be reported to the Sponsor as described in Section 7.5.4. Although the Investigator is not obligated to actively seek this information in former study participants/female partners of male study participants, he or she may learn of an SAE through spontaneous reporting.

- Any female participant who becomes pregnant while participating in the study will be withdrawn from the study.

7.5.6. Cardiovascular and Death Events

For any cardiovascular events detailed in Appendix 8 and all deaths, whether or not they are considered SAEs, specific Cardiovascular and Death sections of the eCRF will be required to be completed. These sections include questions regarding cardiovascular (including sudden cardiac death) and non-cardiovascular death.

The cardiovascular eCRFs are presented as queries in response to reporting of certain cardiovascular Medical Dictionary for Regulatory Activities (MedDRA) terms. The cardiovascular information should be recorded in the specific cardiovascular section of the eCRF within 1 week of receipt of a Cardiovascular Event data query prompting its completion.

The Death eCRF is provided immediately after the occurrence or outcome of death is reported. Initial and follow-up reports regarding death must be completed within 1 week of when the death is reported.

7.5.7. Disease-related Events and/or Disease-related Outcomes Not Qualifying as SAEs

An event which is part of the natural course of the disease under study (i.e., disease progression or hospitalization due to disease progression) does not need to be reported as an SAE. Death due to disease under study is to be recorded on the Death eCRF. However, if the underlying disease (i.e., progression) is greater than that which would normally be expected for the participant or if the Investigator considers that there was a causal relationship between treatment with study treatment(s), protocol design, or protocol procedures and the disease progression, then this must be reported as an SAE.

7.6. Translational Research (Biomarker and Pharmacodynamic Assessments)

Comparative examination of pre-dosing profiles of participants may uncover known or novel candidate biomarkers/profiles that could be used to predict response to PD-1 inhibitors or provide new insights into NSCLC and medically related conditions.

7.6.1. Biomarker Sample Collection

Prior to randomization, mandatory PD-L1 biomarker testing will be performed if no prior result is available at the time of Screening. If no prior results are available, a mandatory archival or fresh tumor specimen must be submitted for testing.

A blood sample for the analysis CCI [redacted] will be collected at the time points specified in the SoA (Table 4).

CCI [redacted]

CCI [redacted]

Details on tissue and blood sample collection, processing, storage, shipping, and handling instructions can be found in the Laboratory Manual.

7.6.2. Tumor Tissue Biomarker Analysis

If no prior PD-L1 result is available at the time of Screening, the mandatory archival or fresh tumor specimen must be submitted to the local or central immunohistochemistry (IHC) laboratory to be evaluated for PD-L1 expression using the 22C3 pharmDx assay (Agilent/Dako) (see also: Section 7.2.3).

CCI [redacted]

7.6.3. Blood (Circulating) Biomarker Analysis

CCI [redacted]

Results from blood-based ctDNA biomarker analyses may be compared with corresponding analyses on tumor tissue samples for concordance, whenever applicable. They may also be correlated with efficacy outcomes.

Remaining blood and/or blood-derived samples such as plasma and ctDNA will be stored for potential future biomarker testing in cancer-related research, including potential bridging to candidate companion diagnostic assays.

7.7. Pharmacokinetics

CCI [redacted]

7.8. Immunogenicity

Serum samples to support analysis of anti-drug antibodies (ADAs) will be collected from all participants (both treatment arms to maintain the blind) according to the SoA (Table 4). ADAs to dostarlimab will be evaluated in the serum samples of those participants receiving dostarlimab and, if needed, ADAs to pembrolizumab will be evaluated in the serum samples of those participants receiving pembrolizumab.

Serum samples will be screened for antibodies binding to dostarlimab and the titer of confirmed positive samples will be reported. All samples collected for the detection of ADAs to dostarlimab will also be evaluated for dostarlimab serum concentration to enable interpretation of the data. ADAs may be further characterized and/or evaluated for their ability to neutralize the activity of dostarlimab based on the positivity of the 3-tier ADA assays. If needed, serum samples for immunogenicity assessment of pembrolizumab will be analyzed in a similar fashion as to those for dostarlimab. Samples will be stored and retained per the Sponsor's sample management SOP or according to local regulations.

7.9. Genetics/Pharmacogenetics

A 6-mL blood sample for DNA isolation will be collected from participants who have consented to participate in the genetics analysis component of the study. Participation is optional. Participants who do not wish to participate in the genetic research may still participate in the study.

In the event of DNA extraction failure, a replacement genetic blood sample may be requested from the participant. Signed informed consent will be required to obtain a replacement sample, unless it was included in the original consent.

See Appendix 9 for information regarding genetic research. Details on processes for the collection, shipment, and destruction of these samples can be found in the Laboratory Manual.

7.10. Value Evidence and Outcomes Assessments

7.10.1. PROs

See the SoA (Table 4) for a list of PRO instruments that will be used to measure the patient-reported assessment of treatment throughout study participation.

PRO questionnaires should be conducted prior to any clinical procedures during the Treatment Period. It is estimated that completion of the PROs will take <20 minutes. The questionnaires will be administered to participants in different regions based on the availability of translated versions.

7.10.1.1. EORTC-QLQ-C30

The European Organisation for Research and Treatment of Cancer Quality of Life Questionnaire 30-item Core Module (EORTC-QLQ-C30) is a questionnaire used to measure health-related quality of life (HRQoL) in patients with cancer; it has been translated and validated in >100 languages and has been used in more than 3,000 studies worldwide . The EORTC-QLQ-C30 is composed of both multi-item scales and single-item measures. These include 5 functional scales (Physical functioning, Role functioning, Emotional functioning, Cognitive functioning, and Social functioning), 3 symptom scales (Fatigue, Nausea and vomiting, and Pain), 6 single items (Dyspnea, Insomnia, Appetite loss, Constipation, Diarrhea, and Financial difficulties), and a global health status/HRQoL. The EORTC-QLQ-C30 employs a week recall period for all items and a 4-point scale for the functional and symptom scales/items with response categories "Not at all," "A little," "Quite a bit," and "Very much." The 2 items assessing global health status/quality of life utilize a 7-point scale ranging from 1 ("Very Poor") to 7 ("Excellent").

7.10.1.2. EORTC-QLQ-LC13

The European Organisation for Research and Treatment of Cancer Quality of Life Questionnaire 13-item Lung Cancer Module (EORTC QLQ-LC13) is a lung cancer specific questionnaire module designed to supplement the EORTC-QLQ-C30 . The measures in the lung cancer questionnaire module assess both lung cancer-associated symptoms such as coughing, hemoptysis, and pain as well as side-effects from

conventional chemo- and radiotherapy such as hair loss, neuropathy, sore mouth and dysphagia. The EORTC QLQ-LC13 is a clinically valid and useful tool for assessing disease- and treatment-specific symptoms in patients with lung cancer participating in clinical studies when combined with the EORTC Core Quality of Life Questionnaire.

7.10.1.3. PRO-CTCAE

The Patient-Reported Outcome Version of the Common Terminology Criteria for Adverse Events (PRO-CTCAE) is a PRO measure developed to evaluate symptomatic toxicity in patients on cancer clinical studies (Basch, Reeve et al. 2014). The PRO-CTCAE was designed to be used as a companion to the CTCAE, the standard lexicon for AE reporting in cancer clinical studies. The PRO-CTCAE includes a library of 124 items representing 78 symptomatic toxicities drawn from the CTCAE. PRO-CTCAE provides a systematic yet flexible tool for descriptive reporting of symptomatic treatment side effects in cancer clinical studies. In the present study, a selection of items from the PRO-CTCAE Version 1.0 Item Library will be administered to participants.

7.10.1.4. FACT-GP5

The Functional Assessment of Cancer Therapy – General Population (FACT-GP) (now in Version 4) is a 27-item compilation of general questions divided into 4 primary quality of life domains: Physical Well-Being, Social/Family Well-Being, Emotional Well-Being, and Functional Well-Being . It is considered appropriate for use with participants with any form of cancer and has also been used and validated in other chronic illness conditions (e.g., HIV/AIDS and multiple sclerosis) and in the general population (using a slightly modified version).

The FACT-GP5 item is a single item from the FACT-G, which assesses how bothersome the side effects of treatment are for patients with cancer. The recall period is the past 7 days, and the item has a 5-category response scale ranging from “0=Not at all” to “4=Very much.” This item is being included to assess the overall tolerability of treatment from the participant’s perspective.

7.10.1.5. Patient Global Impression Items

The Patient Global Impression of Severity (PGIS) assesses the global impression of symptoms severity at baseline and subsequent time points. The second question, the Patient Global Impression of Change (PGIC) serves to rate the global change in symptoms at subsequent time points. In addition to evaluating symptom severity and change, these questions serve as anchors to establish thresholds of clinically meaningful change for the questionnaires in the study .

7.11. Biomedical Research

CCI

[redacted]

CCI [redacted]

In an effort to optimize the research that can be conducted with future biomedical research specimens, it is essential to link study participant clinical study data with future biomedical research test results. The clinical study data allow specific analyses to be conducted. Knowing participant characteristics like sex, age, medical history, treatment type, and treatment outcomes are critical to understanding the clinical context of future biomedical research analytical results.

To maintain privacy of information collected from specimens obtained for future biomedical research, the Sponsor has developed secure policies and procedures. All specimens will be single-coded per International Council for Harmonisation of Technical Requirements for Pharmaceuticals for Human Use (ICH) E15 guidelines, "Definitions for Genomic Biomarkers, Pharmacogenomics, Pharmacogenetics, Genomic Data and Sample Coding Categories" .

At the clinical study site, unique codes will be placed on the future biomedical research specimens for transfer to the storage facility. This first code is a random number that does not contain any personally identifying information embedded within it in order to maintain participant privacy. The link (or key) between participant identifiers and this first unique code will be held at the study site. No personal identifiers will appear on the specimen tube.

Documentation of participant consent for future biomedical research will be captured in the eCRFs. Participants may withdraw their consent for future biomedical research and have their specimens and all derivatives destroyed. Participants may withdraw consent at any time by contacting the Investigator. Any specimens for which an informed consent cannot be verified will be destroyed.

8. STATISTICAL CONSIDERATIONS AND DATA ANALYSES

8.1. Hypothesis

The primary efficacy endpoint, ORR using RECIST v1.1 based on BICR, of dostarlimab plus chemotherapy is similar to that of pembrolizumab plus chemotherapy in participants with metastatic non-squamous NSCLC without a known EGFR, ALK, ROS-1, or BRAF V600E mutation or other genomic aberration for which an approved targeted therapy is available and who have not received prior treatment of metastatic disease.

8.2. Sample Size Considerations

CCI [redacted]

8.3. Data Analysis Considerations

8.3.1. Analysis Populations

The analysis populations are defined as follows (additional analysis populations may be defined in the RAP:

- **Intent-to-treat (ITT) population:** All participants who were randomized. Participants will be analyzed according to the treatment assigned at randomization even if no study treatment was received. Participants who were incorrectly stratified during randomization will be analyzed and presented under the stratum assigned during randomization.
- **Response evaluable population:** A subset of the ITT population that will include participants who do NOT meet any of the following criteria. Participants who meet any of the following criteria will be excluded from the response evaluable population:
  - Participants did not receive at least 1 dose of the randomized study treatment.
  - Participants do not have measurable disease at baseline per Investigator.
  - Participants do not have any post-baseline tumor assessment (unless PD or death is observed before that time, in which case the participant will not be excluded from the response evaluable population).
- **Per-protocol population:** All participants in the ITT population who do not have protocol deviations that may significantly impact the interpretation of efficacy results. A detailed specification of the per-protocol population will be provided prior to database lock. Participants will be analyzed according to the treatment that they actually received.

- **Safety population:** All participants who received at least 1 dose of study treatment. Participants will be analyzed according to the treatment assigned at randomization unless the incorrect treatment(s) was/were received throughout the dosing period, in which case participants will be analyzed according to the first study treatment received.
- **Dostarlimab PK population:** All participants who received any amount of dostarlimab and have at least 1 measurable dostarlimab concentration.
- **Pembrolizumab PK population:** If Pembrolizumab PK analysis is performed, this will include all participants who received any amount of pembrolizumab and have at least 1 measurable pembrolizumab concentration.
- **Dostarlimab immunogenicity population:** All participants who received at least 1 dose of dostarlimab and who have at least 1 ADA sample with an assay result.
- **Pembrolizumab immunogenicity population:** If Pembrolizumab immunogenicity analysis is performed, this will include all participants who received at least 1 dose of pembrolizumab and who have at least 1 ADA sample with an assay result.
- **Biomarker population:** All safety participants who have at least 1 post-baseline tumor assessment and have provided sufficient tumor or blood sample.

8.3.2. Interim Analysis

No formal interim analysis will be done for this study.

8.3.3. Supplementary Analyses

Following the primary analysis with the first Data Cut Off (DCO1) on 4 August 2022, supplementary analyses at DCO2 and at DCO3 are planned to accrue more data to better characterize DOR and overall OS:

1. The supplementary analysis at DCO2 is planned approximately 1 year after DCO1 (15 months after the last subject was randomized).
    - Efficacy endpoints in scope: DOR, OS, ORR.
2. The supplementary analysis at DCO3 is planned approximately 2 years after the last subject was randomized.
    - Efficacy endpoint in scope: OS

Additional analyses may be also conducted at the end of the study.

8.4. Key Elements of Analysis Plan

Data will be listed and summarized according to the GSK reporting standards, where applicable. Complete details will be documented in the RAP. Any deviations from, or additions to, the original analysis plan described in this protocol will be documented in the RAP and final study report.

As it is anticipated that accrual will be spread thinly across centers, and summaries of data by center would be unlikely informative, data from all participating centers will be pooled prior to analysis.

All data up to the time of study completion/withdrawal from study will be included in the analysis, regardless of duration of treatment.

As the duration of treatment for a given participant will depend on efficacy and tolerability, the duration of follow-up will vary between participants. Consequently, there will be no imputation for missing data.

8.4.1. Participant Disposition

[redacted]

8.4.2. Demographics, Medical History, Baseline Characteristics, and Concomitant Medications

[redacted]

8.4.3. Primary Efficacy Analyses

[redacted]

The primary analysis (at DCO1) of ORR will be based on the ITT population, and a supportive sensitivity analysis will be performed on the response evaluable population and per-protocol population.

The primary analysis of ORR will occur after all enrolled participants have completed the third on-study tumor assessment (approximately 6 months) or have been discontinued from the study, whichever occurs first.

The supplementary analysis of ORR will occur approximately 1 year after DCO 1 (about 15 months after the last subject was randomized).

8.4.4. Secondary Efficacy Analyses

[redacted]

8.4.5. Exploratory Efficacy Analyses

CCI [redacted]

8.4.6. Secondary Safety Analyses

CCI [redacted]

8.4.6.1. Extent of Exposure

CCI [redacted]

8.4.6.2. AEs

AEs will be coded using the standard MedDRA and grouped by system organ class. AEs will be graded by the Investigator according to the NCI-CTCAE v5.0.

CCI [redacted]

CCI [redacted]

8.4.6.3. Clinical Laboratory Evaluations

CCI [redacted]

8.4.6.4. Other Safety Measures

CCI [redacted]

CCI

[redacted]

CCI [redacted]

9. REFERENCES

Andrade, R. J., M. Robles and M. I. Lucena (2009). "Rechallenge in drug-induced liver injury: the attractive hazard." Expert Opin Drug Saf **8**(6): 709-714.

Basch, E., B. B. Reeve, S. A. Mitchell, S. B. Clauser, L. M. Minasian, A. C. Dueck, T. R. Mendoza, J. Hay, T. M. Atkinson, A. P. Abernethy, D. W. Bruner, C. S. Cleeland, J. A. Sloan, R. Chilukuri, P. Baumgartner, A. Denicoff, D. St Germain, A. M. O'Mara, A. Chen, J. Kelaghan, A. V. Bennett, L. Sit, L. Rogak, A. Barz, D. B. Paul and D. Schrag (2014). "Development of the National Cancer Institute's patient-reported outcomes version of the common terminology criteria for adverse events (PRO-CTCAE)." J Natl Cancer Inst **106**(9).

Bergman, B., N. K. Aaronson, S. Ahmedzai, S. Kaasa and M. Sullivan (1994). "The EORTC QLQ-LC13: a modular supplement to the EORTC Core Quality of Life Questionnaire (QLQ-C30) for use in lung cancer clinical trials. EORTC Study Group on Quality of Life." Eur J Cancer **30A**(5): 635-642.

Blank, C., I. Brown, A. C. Peterson, M. Spiotto, Y. Iwai, T. Honjo and T. F. Gajewski (2004). "PD-L1/B7H-1 inhibits the effector phase of tumor rejection by T cell receptor (TCR) transgenic CD8+ T cells." Cancer Res **64**(3): 1140-1145.

Borghaei, H., C. J. Langer, S. Gadgeel, V. A. Papadimitrakopoulou, A. Patnaik, S. F. Powell, R. D. Gentzler, R. G. Martins, J. P. Stevenson, S. I. Jalal, A. Panwalkar, J. C. Yang, M. Gubens, L. V. Sequist, M. M. Awad, J. Fiore, S. Saraf, S. M. Keller and L. Gandhi (2019). "24-Month Overall Survival from KEYNOTE-021 Cohort G: Pemetrexed and Carboplatin with or without Pembrolizumab as First-Line Therapy for Advanced Nonsquamous Non-Small Cell Lung Cancer." J Thorac Oncol **14**(1): 124-129.

Borghaei, H., L. Paz-Ares, L. Horn, D. R. Spigel, M. Steins, N. E. Ready, L. Q. Chow, E. E. Vokes, E. Felip, E. Holgado, F. Barlesi, M. Kohlhaufl, O. Arrieta, M. A. Burgio, J. Fayette, H. Lena, E. Poddubskaya, D. E. Gerber, S. N. Gettinger, C. M. Rudin, N. Rizvi, L. Crino, G. R. Blumenschein, Jr., S. J. Antonia, C. Dorange, C. T. Harbison, F. Graf Finckenstein and J. R. Brahmer (2015). "Nivolumab versus Docetaxel in Advanced Nonsquamous Non-Small-Cell Lung Cancer." N Engl J Med **373**(17): 1627-1639.

Brahmer, J., K. L. Reckamp, P. Baas, L. Crino, W. E. Eberhardt, E. Poddubskaya, S. Antonia, A. Pluzanski, E. E. Vokes, E. Holgado, D. Waterhouse, N. Ready, J. Gainor, O. Aren Frontera, L. Havel, M. Steins, M. C. Garassino, J. G. Aerts, M. Domine, L. Paz-Ares, M. Reck, C. Baudelet, C. T. Harbison, B. Lestini and D. R. Spigel (2015). "Nivolumab versus Docetaxel in Advanced Squamous-Cell Non-Small-Cell Lung Cancer." N Engl J Med **373**(2): 123-135.

Brahmer, J. R., C. G. Drake, I. Wollner, J. D. Powderly, J. Picus, W. H. Sharfman, E. Stankevich, A. Pons, T. M. Salay, T. L. McMiller, M. M. Gilson, C. Wang, M. Selby, J. M. Taube, R. Anders, L. Chen, A. J. Korman, D. M. Pardoll, I. Lowy and S. L. Topalian (2010). "Phase I study of single-agent anti-programmed death-1 (MDX-1106) in refractory solid tumors: safety, clinical activity, pharmacodynamics, and immunologic correlates." J Clin Oncol **28**(19): 3167-3175.

Brahmer, J. R., C. Lacchetti, B. J. Schneider, M. B. Atkins, K. J. Brassil, J. M. Caterino, I. Chau, M. S. Ernstoff, J. M. Gardner, P. Ginex, S. Hallmeyer, J. Holter Chakrabarty, N. B. Leighl, J. S. Mammen, D. F. McDermott, A. Naing, L. J. Nastoupil, T. Phillips, L. D. Porter, I. Puzanov, C. A. Reichner, B. D. Santomasso, C. Seigel, A. Spira, M. E. Suarez-Almazor, Y. Wang, J. S. Weber, J. D. Wolchok and J. A. Thompson (2018). "Management of Immune-Related Adverse Events in Patients Treated With Immune Checkpoint Inhibitor Therapy: American Society of Clinical Oncology Clinical Practice Guideline." J Clin Oncol: JCO2017776385.

Cella, D. F., D. S. Tulsky, G. Gray, B. Sarafian, E. Linn, A. Bonomi, M. Silberman, S. B. Yellen, P. Winicour, J. Brannon and et al. (1993). "The Functional Assessment of Cancer Therapy scale: development and validation of the general measure." J Clin Oncol **11**(3): 570-579.

D'Addio, F., L. V. Riella, B. G. Mfarrej, L. Chabtini, L. T. Adams, M. Yeung, H. Yagita, M. Azuma, M. H. Sayegh and I. Guleria (2011). "The link between the PDL1 costimulatory pathway and Th17 in fetomaternal tolerance." J Immunol **187**(9): 4530-4541.

Eisenhauer, E. A., P. Therasse, J. Bogaerts, L. H. Schwartz, D. Sargent, R. Ford, J. Dancey, S. Arbuck, S. Gwyther, M. Mooney, L. Rubinstein, L. Shankar, L. Dodd, R. Kaplan, D. Lacombe and J. Verweij (2009). "New response evaluation criteria in solid tumours: revised RECIST guideline (version 1.1)." Eur J Cancer **45**(2): 228-247.

EORTC-QLQ-C30 (2020). "European Organisation for Research and Treatment of Cancer. EORTC-QLQ-C30 Core Questionnaire.

FDA (2009). Guidance for industry. Drug-induced liver injury: premarketing clinical evaluation. UDoHaHSFaD.

Fridman, W. H., F. Pages, C. Sautes-Fridman and J. Galon (2012). "The immune contexture in human tumours: impact on clinical outcome." Nat Rev Cancer **12**(4): 298-306.

"Gadgeel S, Stevenson J, Langer C, et al. Pembrolizumab (pembro) plus chemotherapy as front-line therapy for advanced NSCLC: KEYNOTE-021 cohorts A-C. J Clin Oncol 34, 2016 (suppl; abstr 9016). ."

Gandhi, L., D. Rodríguez-Abreu, S. Gadgeel, E. Esteban, E. Felip, F. De Angelis, M. Domine, P. Clingan, M. J. Hochmair, S. F. Powell, S. Y.-S. Cheng, H. G. Bischoff, N. Peled, F. Grossi, R. R. Jennens, M. Reck, R. Hui, E. B. Garon, M. Boyer, B. Rubio-Viqueira, S. Novello, T. Kurata, J. E. Gray, J. Vida, Z. Wei, J. Yang, H. Raftopoulos, M. C. Pietanza and M. C. Garassino (2018). "Pembrolizumab plus Chemotherapy in Metastatic Non–Small-Cell Lung Cancer." New England Journal of Medicine **378**(22): 2078-2092.

Guy, W. (1976). ECDEU Assessment Manual for Psychopharmacology, Revised. Rockville, MD, National Institute of Mental Health.

Hato, S. V., A. Khong, I. J. de Vries and W. J. Lesterhuis (2014). "Molecular pathways: the immunogenic effects of platinum-based chemotherapeutics." Clin Cancer Res **20**(11): 2831-2837.

Herbst, R. S., P. Baas, D. W. Kim, E. Felip, J. L. Perez-Gracia, J. Y. Han, J. Molina, J. H. Kim, C. D. Arvis, M. J. Ahn, M. Majem, M. J. Fidler, G. de Castro, Jr., M. Garrido, G. M. Lubiniecki, Y. Shentu, E. Im, M. Dolled-Filhart and E. B. Garon (2016). "Pembrolizumab versus docetaxel for previously treated, PD-L1-positive, advanced non-small-cell lung cancer (KEYNOTE-010): a randomised controlled trial." Lancet **387**(10027): 1540-1550.

Herbst, R. S., J. V. Heymach and S. M. Lippman (2008). "Lung cancer." N Engl J Med **359**(13): 1367-1380.

Herzberg, B., M. J. Campo and J. F. Gainor (2017). "Immune checkpoint inhibitors in non-small cell lung cancer." Oncologist **22**(1): 81-88.

Howlader, N. N., A. M.; Krapcho, M.; Miller, D.; Brest, A.; Yu, M.; Ruhl, J.; Tatalovich, Z.; Mariotto, A.; Lewis, D. R.; Chen, H.S.; Feuer, E.J.; Cronin, K.A. (eds) (2018). " SEER Cancer Statistics Review, 1975-2016, National Cancer Institute. based on November 2018 SEER data submission, posted to the SEER website, April 2019."

Huang, X., F. Venet, Y. L. Wang, A. Lepape, Z. Yuan, Y. Chen, R. Swan, H. Kherouf, G. Monneret, C. S. Chung and A. Ayala (2009). "PD-1 expression by macrophages plays a pathologic role in altering microbial clearance and the innate inflammatory response to sepsis." Proc Natl Acad Sci U S A **106**(15): 6303-6308.

Hunt, C. M. (2010). "Mitochondrial and immunoallergic injury increase risk of positive drug rechallenge after drug-induced liver injury: a systematic review." Hepatology **52**(6): 2216-2222.

ICH E15 (2007). "International Council on Harmonisation. ICH Guideline E15 Definitions for Genomic Biomarkers, Pharmacogenomics, Pharmacogenetics, Genomic Data and Sample Coding Categories.

Ishida, Y., Y. Agata, K. Shibahara and T. Honjo (1992). "Induced expression of PD-1, a novel member of the immunoglobulin gene superfamily, upon programmed cell death." EMBO J **11**(11): 3887-3895.

James, L. P., L. Letzig, P. M. Simpson, E. Capparelli, D. W. Roberts, J. A. Hinson, T. J. Davern and W. M. Lee (2009). "Pharmacokinetics of acetaminophen-protein adducts in adults with acetaminophen overdose and acute liver failure." Drug Metab Dispos **37**(8): 1779-1784.

Karim, R., E. S. Jordanova, S. J. Piersma, G. G. Kenter, L. Chen, J. M. Boer, C. J. Melief and S. H. van der Burg (2009). "Tumor-expressed B7-H1 and B7-DC in relation to PD-1+ T-cell infiltration and survival of patients with cervical carcinoma." Clin Cancer Res **15**(20): 6341-6347.

Keir, M. E., M. J. Butte, G. J. Freeman and A. H. Sharpe (2008). "PD-1 and its ligands in tolerance and immunity." Annu Rev Immunol **26**: 677-704.

KEYTRUDA Prescribing Information (2020). Merck & Co. KEYTRUDA® (pembrolizumab) injection, for intravenous use prescribing information package insert.

Kroemer, G., L. Galluzzi, O. Kepp and L. Zitvogel (2013). "Immunogenic cell death in cancer therapy." Annu Rev Immunol **31**: 51-72.

Langer, C. J., S. M. Gadgeel, H. Borghaei, V. A. Papadimitrakopoulou, A. Patnaik, S. F. Powell, R. D. Gentzler, R. G. Martins, J. P. Stevenson, S. I. Jalal, A. Panwalkar, J. C. Yang, M. Gubens, L. V. Sequist, M. M. Awad, J. Fiore, Y. Ge, H. Raftopoulos, L. Gandhi and K.-. investigators (2016). "Carboplatin and pemetrexed with or without pembrolizumab for advanced, non-squamous non-small-cell lung cancer: a randomised, phase 2 cohort of the open-label KEYNOTE-021 study." Lancet Oncol **17**(11): 1497-1508.

Liechtenstein, T., N. Perez-Janices, M. Gato, F. Caliendo, G. Kochan, I. Blanco-Luquin, K. Van der Jeught, F. Arce, D. Guerrero-Setas, J. Fernandez-Irigoyen, E. Santamaria, K. Breckpot and D. Escors (2014). "A highly efficient tumor-infiltrating MDSC differentiation system for discovery of anti-neoplastic targets, which circumvents the need for tumor establishment in mice." Oncotarget **5**(17): 7843-7857.

Mok, T. S. K., Y. L. Wu, I. Kudaba, D. M. Kowalski, B. C. Cho, H. Z. Turna, G. Castro, Jr., V. Srimuninnimit, K. K. Laktionov, I. Bondarenko, K. Kubota, G. M. Lubiniecki, J. Zhang, D. Kush, G. Lopes and K.-. Investigators (2019). "Pembrolizumab versus chemotherapy for previously untreated, PD-L1-expressing, locally advanced or metastatic non-small-cell lung cancer (KEYNOTE-042): a randomised, open-label, controlled, phase 3 trial." Lancet **393**(10183): 1819-1830.

Motzer, R. J., B. Escudier, D. F. McDermott, S. George, H. J. Hammers, S. Srinivas, S. S. Tykodi, J. A. Sosman, G. Procopio, E. R. Plimack, D. Castellano, T. K. Choueiri, H. Gurney, F. Donskov, P. Bono, J. Wagstaff, T. C. Gauler, T. Ueda, Y. Tomita, F. A. Schutz, C. Kollmannsberger, J. Larkin, A. Ravaud, J. S. Simon, L. A. Xu, I. M. Waxman, P. Sharma and I. CheckMate (2015). "Nivolumab versus everolimus in advanced renal-cell carcinoma." N Engl J Med **373**(19): 1803-1813.

NCI SEER Lung Cancer (2020). "National Cancer Institute Surveillance, Epidemiology and End Results (SEER Program): Cancer Stats Facts - Lung and Bronchus Cancer 2012-2016.

NCI SEER NSCLC (2018). "Surveillance, Epidemiology, and End Results (SEER) Program SEER*Stat Database: Incidence - SEER 18 Regs Research Data + Hurricane Katrina Impacted Louisiana Cases, Nov 2018 Sub (2000-2016) <Katrina/Rita Population Adjustment> - Linked To County Attributes - Total U.S., 1969-2017 Counties, National Cancer Institute, DCCPS, Surveillance Research Program, released April 2019, based on the November 2018 submission."

Nishimura, H., Y. Agata, A. Kawasaki, M. Sato, S. Imamura, N. Minato, H. Yagita, T. Nakano and T. Honjo (1996). "Developmentally regulated expression of the PD-1 protein on the surface of double-negative (CD4-CD8-) thymocytes." Int Immunol **8**(5): 773-780.

Oken, M. M., R. H. Creech, D. C. Tormey, J. Horton, T. E. Davis, E. T. McFadden and P. P. Carbone (1982). "Toxicity and response criteria of the Eastern Cooperative Oncology Group." Am J Clin Oncol **5**(6): 649-655.

Pages, F., A. Berger, M. Camus, F. Sanchez-Cabo, A. Costes, R. Molidor, B. Mlecnik, A. Kirilovsky, M. Nilsson, D. Damotte, T. Meatchi, P. Bruneval, P. H. Cugnenc, Z. Trajanoski, W. H. Fridman and J. Galon (2005). "Effector memory T cells, early metastasis, and survival in colorectal cancer." N Engl J Med **353**(25): 2654-2666.

Papadimitrakopoulou, V., M. Cobo, R. Bordoni, P. Dubray-Longeras, Z. Szalai, G. Ursol, S. Novello, F. Orlandi, S. Ball, J. Goldschmidt, Jr., R. Sanborn, T. Hoang, D. Mendus, Y. Deng, M. Kowanetz, X. Wen, W. Lin, A. Sandler and M. Nishio (2018). "OA05.07 IMpower132: PFS and Safety Results with 1L Atezolizumab + Carboplatin/Cisplatin + Pemetrexed in Stage IV Non-Squamous NSCLC." Journal of Thoracic Oncology **13**(10): S332-S333.

Papay, J. I., D. Clines, R. Rafi, N. Yuen, S. D. Britt, J. S. Walsh and C. M. Hunt (2009). "Drug-induced liver injury following positive drug rechallenge." Regul Toxicol Pharmacol **54**(1): 84-90.

Paz-Ares, L., A. Luft, D. Vicente, A. Tafreshi, M. Gumus, J. Mazieres, B. Hermes, F. Cay Senler, T. Csoszi, A. Fulop, J. Rodriguez-Cid, J. Wilson, S. Sugawara, T. Kato, K. H. Lee, Y. Cheng, S. Novello, B. Halmos, X. Li, G. M. Lubiniecki, B. Piperdi, D. M. Kowalski and K.-. Investigators (2018). "Pembrolizumab plus Chemotherapy for Squamous Non-Small-Cell Lung Cancer." N Engl J Med **379**(21): 2040-2051.

Pedoeem, A., I. Azoulay-Alfaguter, M. Strazza, G. J. Silverman and A. Mor (2014). "Programmed death-1 pathway in cancer and autoimmunity." Clin Immunol **153**(1): 145-152.

Pena-Cruz, V., S. M. McDonough, F. Diaz-Griffero, C. P. Crum, R. D. Carrasco and G. J. Freeman (2010). "PD-1 on immature and PD-1 ligands on migratory human Langerhans cells regulate antigen-presenting cell activity." J Invest Dermatol **130**(9): 2222-2230.

Pfannenstiel, L. W., S. S. Lam, L. A. Emens, E. M. Jaffee and T. D. Armstrong (2010). "Paclitaxel enhances early dendritic cell maturation and function through TLR4 signaling in mice." Cell Immunol **263**(1): 79-87.

Reck, M., D. Rodriguez-Abreu, A. G. Robinson, R. Hui, T. Csoszi, A. Fulop, M. Gottfried, N. Peled, A. Tafreshi, S. Cuffe, M. O'Brien, S. Rao, K. Hotta, M. A. Leiby, G. M. Lubiniecki, Y. Shentu, R. Rangwala, J. R. Brahmer and K.-. Investigators (2016). "Pembrolizumab versus Chemotherapy for PD-L1-Positive Non-Small-Cell Lung Cancer." N Engl J Med **375**(19): 1823-1833.

Robert, C., G. V. Long, B. Brady, C. Dutriaux, M. Maio, L. Mortier, J. C. Hassel, P. Rutkowski, C. McNeil, E. Kalinka-Warzocha, K. J. Savage, M. M. Hernberg, C. Lebbe, J. Charles, C. Mihalcioiu, V. Chiarion-Sileni, C. Mauch, F. Cognetti, A. Arance, H. Schmidt, D. Schadendorf, H. Gogas, L. Lundgren-Eriksson, C. Horak, B. Sharkey, I. M. Waxman, V. Atkinson and P. A. Ascierto (2015). "Nivolumab in previously untreated melanoma without BRAF mutation." N Engl J Med **372**(4): 320-330.

Robert, C., J. Schachter, G. V. Long, A. Arance, J. J. Grob, L. Mortier, A. Daud, M. S. Carlino, C. McNeil, M. Lotem, J. Larkin, P. Lorigan, B. Neyns, C. U. Blank, O. Hamid, C. Mateus, R. Shapira-Frommer, M. Kosh, H. Zhou, N. Ibrahim, S. Ebbinghaus, A. Ribas and K.-. investigators (2015). "Pembrolizumab versus Ipilimumab in Advanced Melanoma." N Engl J Med **372**(26): 2521-2532.

Sanmamed, M. F. and L. Chen (2014). "Inducible expression of B7-H1 (PD-L1) and its selective role in tumor site immune modulation." Cancer J **20**(4): 256-261.

Santarpia, M., N. Karachaliou and R. Rosell (2017). "Beyond platinum treatment for NSCLC: what does the future hold?" Expert Rev Anticancer Ther **17**(4): 293-295.

Senovilla, L., I. Vitale, I. Martins, M. Tailler, C. Pailleret, M. Michaud, L. Galluzzi, S. Adjemian, O. Kepp, M. Niso-Santano, S. Shen, G. Marino, A. Criollo, A. Boileve, B. Job, S. Ladoire, F. Ghiringhelli, A. Sistigu, T. Yamazaki, S. Rello-Varona, C. Locher, V. Poirier-Colame, M. Talbot, A. Valent, F. Berardinelli, A. Antoccia, F. Ciccosanti, G. M. Fimia, M. Piacentini, A. Fueyo, N. L. Messina, M. Li, C. J. Chan, V. Sigl, G. Pourcher, C. Ruckenstuhl, D. Carmona-Gutierrez, V. Lazar, J. M. Penninger, F. Madeo, C. Lopez-Otin, M. J. Smyth, L. Zitvogel, M. Castedo and G. Kroemer (2012). "An immunosurveillance mechanism controls cancer cell ploidy." Science **337**(6102): 1678-1684.

Sevko, A., T. Michels, M. Vrohlings, L. Umansky, P. Beckhove, M. Kato, G. V. Shurin, M. R. Shurin and V. Umansky (2013). "Antitumor effect of paclitaxel is mediated by inhibition of myeloid-derived suppressor cells and chronic inflammation in the spontaneous melanoma model." J Immunol **190**(5): 2464-2471.

Socinski, M. A., R. M. Jotte, F. Cappuzzo, F. Orlandi, D. Stroyakovskiy, N. Nogami, D. Rodriguez-Abreu, D. Moro-Sibilot, C. A. Thomas, F. Barlesi, G. Finley, C. Kelsch, A. Lee, S. Coleman, Y. Deng, Y. Shen, M. Kowanetz, A. Lopez-Chavez, A. Sandler, M. Reck and I. M. S. Group (2018). "Atezolizumab for First-Line Treatment of Metastatic Nonsquamous NSCLC." N Engl J Med **378**(24): 2288-2301.

Spiess, P. E., J. Dhillon, A. S. Baumgarten, P. A. Johnstone and A. R. Giuliano (2016). "Pathophysiological basis of human papillomavirus in penile cancer: Key to prevention and delivery of more effective therapies." CA Cancer J Clin **66**(6): 481-495.

Taube, J. M., R. A. Anders, G. D. Young, H. Xu, R. Sharma, T. L. McMiller, S. Chen, A. P. Klein, D. M. Pardoll, S. L. Topalian and L. Chen (2012). "Colocalization of inflammatory response with B7-h1 expression in human melanocytic lesions supports an adaptive resistance mechanism of immune escape." Sci Transl Med **4**(127): 127ra137.

Topalian, S. L., C. G. Drake and D. M. Pardoll (2012). "Targeting the PD-1/B7-H1(PD-L1) pathway to activate anti-tumor immunity." Curr Opin Immunol **24**(2): 207-212.

Weber, J. S., S. P. D'Angelo, D. Minor, F. S. Hodi, R. Gutzmer, B. Neyns, C. Hoeller, N. I. Khushalani, W. H. Miller, Jr., C. D. Lao, G. P. Linette, L. Thomas, P. Lorigan, K. F. Grossmann, J. C. Hassel, M. Maio, M. Sznol, P. A. Ascierto, P. Mohr, B. Chmielowski, A. Bryce, I. M. Svane, J. J. Grob, A. M. Krackhardt, C. Horak, A. Lambert, A. S. Yang and J. Larkin (2015). "Nivolumab versus chemotherapy in patients with advanced melanoma who progressed after anti-CTLA-4 treatment (CheckMate 037): a randomised, controlled, open-label, phase 3 trial." Lancet Oncol **16**(4): 375-384.

West, H., M. McCleod, M. Hussein, A. Morabito, A. Rittmeyer, H. J. Conter, H. G. Kopp, D. Daniel, S. McCune, T. Mekhail, A. Zer, N. Reinmuth, A. Sadiq, A. Sandler, W. Lin, T. Ochi Lohmann, V. Archer, L. Wang, M. Kowanetz and F. Cappuzzo (2019). "Atezolizumab in combination with carboplatin plus nab-paclitaxel chemotherapy compared with chemotherapy alone as first-line treatment for metastatic non-squamous non-small-cell lung cancer (IMpower130): a multicentre, randomised, open-label, phase 3 trial." Lancet Oncol **20**(7): 924-937.

Yao, S. and L. Chen (2014). "PD-1 as an immune modulatory receptor." Cancer Journal **20**(4): 26.

Zhang, L., J. R. Conejo-Garcia, D. Katsaros, P. A. Gimotty, M. Massobrio, G. Regnani, A. Makrigiannakis, H. Gray, K. Schlienger, M. N. Liebman, S. C. Rubin and G. Coukos (2003). "Intratumoral T cells, recurrence, and survival in epithelial ovarian cancer." N Engl J Med **348**(3): 203-213.

Zhang, L., T. F. Gajewski and J. Kline (2009). "PD-1/PD-L1 interactions inhibit antitumor immune responses in a murine acute myeloid leukemia model." Blood **114**(8): 1545-1552.

Zou, W. and L. Chen (2008). "Inhibitory B7-family molecules in the tumour microenvironment." Nat Rev Immunol **8**(6): 467-477.

APPENDIX 1. GUIDELINES FOR ASSESSMENT OF DISEASE, DISEASE PROGRESSION, AND RESPONSE CRITERIA

Adapted from RECIST v1.1 (Eisenhauer, Therasse et al. 2009).

Assessment Guidelines

Please note the following:

- The same diagnostic method, including use of contrast when applicable, must be used throughout the study to evaluate a lesion. Contrast agents must be used in accordance with the Image Acquisition Guidelines.
- All measurements should be taken and recorded in millimeters (mm), using a ruler or calipers.
- Ultrasound is not a suitable modality of disease assessment. If new lesions are identified by ultrasound, then confirmation by computed tomography (CT) or magnetic resonance imaging (MRI) is required.
- Fluorodeoxyglucose-positron emission tomography (FDG-PET) is generally not suitable for ongoing assessments of disease. However, FDG-PET can be useful in confirming new sites of disease where a positive FDG-PET scan correlates with the new site of disease present on CT/MRI. FDG-PET may also be used in lieu of a standard bone scan, providing that coverage allows interrogation of all likely sites of bone disease and FDG-PET is performed at all assessments.
- If PET/CT is performed, then the CT component can only be used for standard response assessments if performed to diagnostic quality, which includes the required anatomical coverage and prescribed use of contrast. The method of assessment should be noted as CT on the electronic case report form (eCRF).

CT and MRI: Contrast enhanced CT with 5 mm contiguous slices is recommended.

The minimum size of a measurable baseline lesion should be twice the slice thickness, with a minimum lesion size of 10 mm when the slice thickness is 5 mm. MRI is acceptable, but when used, the technical specification of the scanning sequences should be optimized for the evaluation of the type and site of disease and lesions must be measured in the same anatomic plane by use of the same imaging examinations. Whenever possible the same scanner should be used (Eisenhauer, Therasse et al. 2009).

X-ray: In general, X-ray should not be used for target lesion measurements owing to poor lesion definition. Lesions on chest X-ray may be considered for assessing progression due to new lesions, if they are clearly defined and surrounded by aerated lung; however, chest CT is preferred over chest X-ray (Eisenhauer, Therasse et al. 2009).

Brain scan: If brain scans are required, then contrast enhanced MRI is preferable to contrast enhanced CT.

Bone scan (typically bone scintigraphy): If a bone scan is performed and a new lesion(s) is equivocal, then correlative imaging (i.e., X-ray, CT, or MRI) is required to demonstrate malignant characteristics of the lesion(s).

Note: PET [FDG or fluoride] may be used in lieu of a standard bone scan providing coverage allows interrogation of all likely sites of bone disease and PET is performed at all assessments.

Guidelines for Evaluation of Disease

Measurable and Non-measurable Definitions

- **Measurable lesion:** A non-nodal lesion that can be accurately measured in at least 1 dimension (longest dimension) of the following:
  - ≥10 mm in long-axis diameter with MRI or CT when the scan slice thickness is no greater than 5 mm. If the slice thickness is greater than 5 mm, the minimum size of a measurable lesion must be at least double the slice thickness (e.g., if the slice thickness is 10 mm, a measurable lesion must be ≥20 mm).

  Additionally, lymph nodes can be considered pathologically enlarged and measurable if:
  - ≥15 mm in the short axis when assessed by CT or MRI (slice thickness recommended to be no more than 5 mm). At baseline and Follow-up, only the short axis will be measured (Eisenhauer, Therasse et al. 2009).
- **Non-measurable lesion:** All other lesions including lesions too small to be considered measurable (longest diameter <10 mm or pathological lymph nodes with ≥10 mm and <15 mm short axis) and truly non-measurable lesions, which include: leptomeningeal disease, ascites, pleural or pericardial effusions, inflammatory breast disease, lymphangitic involvement of the skin or lung, and abdominal masses/abdominal organomegaly identified by physical examination that is not measurable by reproducible imaging techniques (Eisenhauer, Therasse et al. 2009).
- **Measurable disease**: The presence of at least 1 measurable lesion. Palpable lesions that are not measurable by radiologic or photographic evaluations may not be utilized as the only measurable lesion.
- **Non-measurable only disease**: The presence of only non-measurable lesions.

  Note: Non-measurable only disease is not allowed per protocol.

Response Criteria

Evaluation of Target Lesions

Definitions for assessment of response for target lesion(s) are as follows:

- **Complete response (CR):** Disappearance of all target lesions. Any pathological lymph nodes must be <10 mm in the short axis.
- **Partial response (PR):** At least a 30% decrease in the sum of the diameters of target lesions, taking as reference the baseline sum diameters (e.g., percent change from baseline).
- **Stable disease (SD):** Neither sufficient shrinkage to qualify for PR nor sufficient increase to qualify for PD.

- **Progressive disease (PD):** At least a 20% increase in the sum of the diameters of target lesions, taking as a reference the smallest sum of diameters recorded since the treatment started (e.g., percent change from nadir, where nadir is defined as the smallest sum of diameters recorded since treatment start). In addition, the sum must have an absolute increase from nadir of 5 mm.
- **Not applicable (NA):** No target lesions at baseline.
- **Not evaluable (NE):** Cannot be classified by 1 of the 5 preceding definitions.

Notes

- If lymph nodes are documented as target lesions, the short axis is added into the sum of diameters (e.g., sum of diameters is the sum of the longest diameters for non-nodal lesions and the short axis for nodal lesions). When lymph nodes decrease to non-pathological size (short axis <10 mm) they should still have a measurement reported in order not to overstate progression.
- If at a given assessment time point all target lesions identified at baseline are not assessed, the sum of diameters cannot be calculated for purposes of assessing CR, PR, or SD or for use as the nadir for future assessments. However, the sum of the diameters of the assessed lesions and the percent change from nadir should be calculated to ensure that progression has not been documented. If an assessment of PD cannot be made, the response assessment should be NE.
- All lesions (nodal and non-nodal) should have their measurements recorded even when very small (e.g., 2 mm). If lesions are present but too small to measure, then 5 mm should be recorded and should contribute to the sum of diameters, unless it is likely that the lesion has disappeared, in which case 0 mm should be reported.
- If a lesion disappears and reappears at a subsequent time point, then it should continue to be measured. The response at the time when the lesion reappears will depend on the status of the other lesions. For example, if the disease had reached a CR status then PD would be documented at the time of reappearance. However, if the response status was PR or SD, the diameter of the reappearing lesion should be added to the remaining diameters and response should be determined based on percent change from baseline and percent change from nadir.

Evaluation of Non-Target Lesions

Definitions for assessment of response for non-target lesion(s) are as follows:

- **CR:** The disappearance of all non-target lesions. All lymph nodes identified as a site of disease at baseline must be non-pathological (e.g., <10 mm short axis).
- **Non-CR/non-PD:** The persistence of 1 or more non-target lesion(s) or lymph nodes identified as a site of disease at baseline with ≥10 mm short axis.
- **PD:** Unequivocal progression of existing non-target lesions. A modest increase in the size of 1 or more non-target lesions is usually not sufficient to qualify for unequivocal progression status. The designation of overall progression solely on the basis of change in non-target disease in the face of SD or PR of target disease will therefore be extremely rare.

- **NA:** No non-target lesions at baseline.
- **NE:** Cannot be classified by 1 of the 4 preceding definitions.

Notes

- In the presence of measurable disease, progression on the basis of solely non-target disease requires substantial worsening such that, even in the presence of SD or PR in target disease, the overall tumor burden has increased sufficiently to merit discontinuation of therapy.
- Sites of non-target lesions that are not assessed at a particular time point based on the assessment schedule should be excluded from the response determination (e.g., non-target response does not have to be "Not Evaluable").

New Lesions

- New malignancies denoting disease progression must be unequivocal. Lesions identified in Follow-up in an anatomical location not scanned at baseline are considered new lesions.
- Any equivocal new lesions should continue to be followed. Treatment can continue at the discretion of the Investigator until the next scheduled assessment. If at the next assessment the new lesion is considered to be unequivocal, progression should be documented.

Evaluation of Overall Response

Table 17 presents the overall response at an individual time point for all possible combinations of tumor responses in target and non-target lesions with or without the appearance of new lesions for participants with measurable disease at baseline.

Table 17: Evaluation of Overall Response for Participants with Measurable Disease at Baseline

Target Lesions	Non-target Lesions	New Lesions	Overall Response
CR	CR or NA	No	CR
CR	Non-CR/non-PD or NE	No	PR
PR	Non-PD or NA or NE	No	PR
SD	Non-PD or NA or NE	No	SD
NE	Non-PD or NA or NE	No	NE
PD	Any	Yes or no	PD
Any	PD	Yes or no	PD
Any	Any	Yes	PD

Abbreviations: CR=complete response; NA=not applicable; NE=not evaluable; PD=progressive disease; PR=partial response.

Notes

- Participants with a global deterioration of health status requiring discontinuation of treatment without objective evidence of disease progression at that time should be classified as having "symptomatic deterioration." Objective response status is determined by evaluations of disease burden. Every effort should be made to document the objective progression even after discontinuation of treatment.
- In some circumstances, it may be difficult to distinguish residual disease from normal tissue. When the evaluation of CR depends on this determination, it is recommended that the residual lesion be investigated (fine-needle aspirate/biopsy) to confirm the CR.

Evaluation of BOR

The best overall response (BOR) is the best response recorded from the start of the treatment until disease progression/recurrence and will be determined programmatically by GSK based on the Investigator's assessment of response at each time point.

- To be assigned a status of SD, follow-up disease assessment must have met the SD criteria at least once after randomization at a minimum interval of 35 (±7) days.
- If the minimum time for SD is not met, best response will depend on the subsequent assessments. For example, if an assessment of PD follows the assessment of SD and SD does not meet the minimum time requirement, the best response will be PD. Alternatively, participants lost to follow-up after an SD assessment not meeting the minimum time criteria will be considered not evaluable.
- Confirmation of disease is not required per protocol.

Evaluation of Scans Subsequent to RECIST v1.1-defined PD

For clinically stable participant treated through a RECIST v1.1-defined radiological progression, a follow-up scan is requested at least 4 weeks and no longer than 8 weeks later. The follow-up scan provides additional information to the Investigator for participant management and further treatment decisions. As the published RECIST v1.1 criteria (Eisenhauer, Therasse et al. 2009) do not provide guidance on how to assess scans acquired after RECIST v1.1-defined PD, supplemental instructions for Investigators on how to evaluate these follow-up scans are as follows:

A subsequent follow-up scan would be considered as having PD if *any* of the following criteria are met:

- ≥20% increase and at least a 5-mm increase in the sum of diameters of target lesions compared with the nadir sum of diameters at 2 consecutive visits, and a further increase of ≥5 mm in the sum of diameters at the follow-up scan timepoint compared with the immediate prior time point
- Significant progression (worsening) of non-target lesions at the follow-up scan timepoint compared with the immediately prior scan time point
- Significant progression (worsening) of previously new lesions (pre-existing new lesions) at the follow-up scan time point compared with the immediately prior scan time point

- Additional brand-new unequivocal lesions at the follow-up scan time point

APPENDIX 2. EASTERN COOPERATIVE ONCOLOGY GROUP PERFORMANCE STATUS

Table 18: Eastern Cooperative Oncology Group (ECOG) Performance Status Grading

Description	Grade
Fully active, able to carry on all pre-disease performance without restriction.	0
Restricted in physically strenuous activity, but ambulatory and able to carry out work of a light or sedentary nature (i.e., light house work, office work).	1
Ambulatory and capable of all self-care but unable to carry out any work activities. Up and about more than 50% of waking hours.	2
Capable of only limited self-care, confined to bed or chair more than 50% of waking hours.	3
Completely disabled. Cannot carry on any self-care. Totally confined to bed or chair.	4

Source: (Oken Creech et al. 1982)

APPENDIX 3. CLINICAL LABORATORY TESTS

Screening Only Testing

HIV and hepatitis B and C testing performed at Screening (or within 3 months prior to first dose of study treatment) only; laboratory tests for:

- HIV-1 or HIV-2 antibodies
- Hepatitis B surface antigen and hepatitis C antibodies

Continuous Laboratory Assessments

Parameters assessed throughout the study are listed in Table 19. Laboratory assessments required for the start of each treatment cycle may be performed up to 72 hours prior to the day of treatment.

Table 19: Protocol-required Safety Laboratory Tests

Laboratory Assessment	Parameters
Complete blood count (CBC)	• Differential white blood cell count<br>• Hematocrit (Hct)<br>• Hemoglobin<br>• Mean corpuscular volume (MCV)<br>• Platelet count<br>• Red blood cell count (RBC or erythrocyte count)<br>• White blood cell count
Serum chemistry	• Alanine aminotransferase (ALT)<br>• Albumin<br>• Alkaline phosphatase (ALP)<br>• Amylase<br>• Aspartate aminotransferase (AST)<br>• Blood urea nitrogen (if not available, then urea)<br>• Calcium<br>• Chloride<br>• Creatinine<br>• Gamma-glutamyl transferase (GGT)<br>• Glucose<br>• Lactate dehydrogenase<br>• Magnesium<br>• Phosphate<br>• Potassium<br>• Sodium<br>• Total bilirubin<br>• Total protein

Table 19: Protocol-required Safety Laboratory Tests (Continued)

**Laboratory Assessment**	**Parameters**
Urinalysis	• Bilirubin<br>• Blood<br>• Glucose<br>• Ketones<br>• Leukocyte esterase<br>• Microscopy (if clinically indicated)<br>• Nitrite<br>• Protein<br>• Specific gravity
Thyroid panel	• Thyroid-stimulating hormone (TSH)<br>• Triiodothyronine (T3) or free triiodothyronine (FT3)<br>• Thyroxine (T4) or free thyroxine (FT4)<br>(or equivalent tests, where applicable)
Pregnancy test	• Highly sensitive serum or urine human chorionic gonadotropin (hCG) pregnancy test (as needed for women of childbearing potential)[1]
Other testing	• Follicle-stimulating hormone (FSH) and estradiol (as needed in women of non-childbearing potential only)

[1] Post-screening, local urine testing will be standard for the protocol, unless serum testing is required by local regulation or Institutional Review Board/Independent Ethics Committee.

APPENDIX 4. REGULATORY, ETHICAL, AND STUDY OVERSIGHT CONSIDERATIONS

Regulatory and Ethical Considerations

This study will be conducted in accordance with the protocol and with the following:

- Consensus ethical principles derived from international guidelines including the Declaration of Helsinki and Council for International Organizations of Medical Sciences International Ethical Guidelines
- Applicable International Council for Harmonisation of Technical Requirements for Pharmaceuticals for Human Use (ICH) Good Clinical Practice (GCP) Guidelines
- Applicable laws and regulations

The protocol, protocol amendments, informed consent form (ICF), Investigator's Brochure, and other relevant documents (e.g., advertisements) must be submitted to an Institutional Review Board (IRB)/Independent Ethics Committee (IEC) by the Investigator and reviewed and approved by the IRB/IEC before the study is initiated.

Any amendments to the protocol will require IEC/IRB approval before implementation of changes made to the study design, except for changes necessary to eliminate an immediate hazard to study participants.

Protocols and any substantial amendments to the protocol will require health authority approval prior to initiation, except for changes necessary to eliminate an immediate hazard to study participants.

The Investigator will be responsible for the following:

- Providing written summaries of the status of the study to the IRB/IEC annually, or more frequently, in accordance with the requirements, policies, and procedures established by the IRB/EC
- Notifying the IRB/IEC of SAEs or other significant safety findings, as required by IRB/IEC procedures
- Providing oversight of the conduct of the study at the site and adherence to requirements of the United States Food and Drug Administration 21 Code of Federal Regulations (CFR), ICH guidelines, the IRB/IEC, European regulation 536/2014 for clinical studies (if applicable), European Medical Device Regulation 2017/745 for clinical device research (if applicable), and all other applicable local regulations

Financial Disclosure

Investigators and sub-Investigators will provide the Sponsor with sufficient, accurate financial information as requested to allow the Sponsor to submit complete and accurate financial certification or disclosure statements to the appropriate regulatory authorities. Investigators are responsible for providing information on financial interests during the course of the study and for 1 year after completion of the study.

Informed Consent Process

- The Investigator or their representative will explain the nature of the study to the participants or their legally authorized representative and answer all questions regarding the study.
- Participants must be informed that their participation is voluntary. Participants or their legally authorized representative will be required to sign a statement of informed consent that meets the requirements of 21 CFR 50, local regulations, ICH guidelines, Health Insurance Portability and Accountability Act requirements, where applicable, and the IRB/IEC or study center.
- The medical record must include a statement that written informed consent was obtained before the participant was enrolled in the study and the date the written consent was obtained. The authorized person obtaining the informed consent must also sign the ICF.
- Participants must be re-consented to the most current version of the ICF(s) during their participation in the study.
- A copy of the ICF(s) must be provided to the participants or their legally authorized representative.
- A participant who is rescreened is not required to sign another ICF if the rescreening occurs within 28 days from the previous ICF signature date.
- GSK (alone or working with others) may use participant's coded study data and samples and other information to carry out this study, understand the results of this study, learn more about the study treatment or about the study disease, publish the results of these research efforts, and/or work with government agencies or insurers to have the study treatment approved for medical use or approved for payment coverage.
- The ICF contains a separate section that addresses the use of participant data and remaining samples for optional further research. The Investigator or authorized designee will inform each participant of the possibility of further research not related to the study/disease. Participants will be told that they are free to refuse to participate and may withdraw their consent at any time and for any reason during the storage period. A separate signature will be required to document a participant's agreement to allow any participant data and/or remaining leftover samples to be used for further research not related to the study/disease. Participants who decline further research will tick the corresponding “No” box.

Data Protection

- Participants will be assigned a unique identifier by the Sponsor. Any participant records or datasets that are transferred to the Sponsor will contain the identifier only; participant names or any information that would make the participant identifiable will not be transferred.
- The participant must be informed that their personal study-related data will be used by the Sponsor in accordance with local data protection law. The level of disclosure

must also be explained to the participant who will be required to give consent for their data to be used as described in the informed consent.

- The participant must be informed that his or her medical records may be examined by Clinical Quality Assurance auditors or other authorized personnel appointed by the Sponsor, by appropriate IRB/IEC members, and by inspectors from regulatory authorities.

Committees Structure

A blinded independent central review (BICR) will be utilized in this study for review of radiological images and assessment of tumor response and progression. Details of the review will be summarized in the BICR Charter.

To safeguard the interest and safety of the participants in the study, a safety monitoring/review panel will be used to conduct review of incoming data to monitor emerging safety signals. If the panel recommends modifications to the design of the protocol or discontinuation of the study, selected Sponsor representatives may be unblinded in order to act on these recommendations.

Dissemination of Clinical Study Data

- Where required by applicable regulatory requirements, an Investigator signatory will be identified for the approval of the CSR. The Investigator will be provided reasonable access to statistical tables, figures, and relevant reports and will have the opportunity to review the complete study results at a GSK site or other mutually agreeable location.
- GSK will also provide all Investigators who were involved in the study with the full summary of the study results. The Investigator(s) is (are) encouraged to share the summary results with the study participants, as appropriate.
- Under the framework of the SHARE initiative, GSK intends to make anonymized participant-level data from this study available to external researchers for scientific analyses or to conduct further research that can help advance medical science or improve patient care. This helps ensure that the data provided by study participants are used to maximum effect in the creation of knowledge and understanding. Requests for access may be made through www.clinicalstudydatarequest.com.
- GSK will provide the Investigator with the randomization codes for their site only after completion of the full statistical analysis.
- A manuscript will be progressed for publication in the scientific literature if the results provide important scientific or medical knowledge.

Data Quality Assurance

- All participant data relating to the study will be recorded on the electronic case report form (eCRF), unless transmitted to the Sponsor or designee electronically (e.g., laboratory data). The Investigator is responsible for verifying that data entries are accurate and correct by signing the eCRF.

- Guidance on completion of eCRFs will be provided in the Case Report Form Completion Guidelines.
- Quality tolerance limits (QTLs) will be pre-defined in the Study Reference Manual to identify systematic issues that can impact participant safety and/or reliability of study results. These pre-defined parameters will be monitored during the study, and deviations from the QTLs and remedial actions taken will be summarized in the CSR.
- The Investigator must permit study-related monitoring, audits, IRB/IEC review, and regulatory agency inspections and must provide direct access to source data documents.
- Monitoring details describing strategy, including definition of study critical data items and processes (e.g., risk-based initiatives in operations and quality such as Risk Management and Mitigation Strategies and Analytical Risk-Based Monitoring, involvement of central reading mechanism), methods, responsibilities, and requirements, including handling of noncompliance issues and monitoring techniques (central, remote, or on-site monitoring) are provided in the Monitoring Plan.
- The Sponsor or designee is responsible for the data management of this study including quality checking of the data.
- The Sponsor assumes accountability for actions delegated to other individuals (e.g., Contract Research Organizations [CROs]).
- Records and documents, including signed ICF, pertaining to the conduct of this study must be retained by the Investigator for 25 years from the issue of the final CSR/equivalent summary, unless local regulations or institutional policies require a longer retention period. No records may be destroyed during the retention period without the written approval of the Sponsor. No records may be transferred to another location or party without written notification to the Sponsor.
- When copies of source documents are shared externally for review by a central reader mechanism (e.g., endpoint adjudication committee; expert reader), documents are stored by the external body for 25 years

Source Documents

- Source documents provide evidence for the existence of the participant and substantiate the integrity of the data collected. Source documents are filed at the Investigator’s site.
- Data reported on the eCRF that are transcribed from source documents must be consistent with the source documents or the discrepancies must be explained. The Investigator may need to request previous medical records or transfer records, depending on the study. Also, current medical records must be available.
- Definition of what constitutes source data and its origin can be found in the Study Reference Manual.
- The Investigator must maintain accurate documentation (source data) that supports the information entered in the eCRF.

- Study monitors will perform ongoing source data verification to confirm that data entered into the eCRF by authorized site personnel are accurate, complete, and verifiable from source documents; that the safety and rights of participants are being protected; and that the study is being conducted in accordance with the currently approved protocol and any other study agreements, ICH GCP, and all applicable regulatory requirements.
- Copies of documents are shared with external third parties contracted by GSK for review by a central reader mechanism (e.g., endpoint adjudication committee; expert reader). The non-exhaustive list of documents shared to inform the central reader may include, discharge summaries, imaging reports, ECG reports etc. Participant names or any information which would make the participant identifiable or is not essential for the central reader mechanism will be redacted by the investigator sites prior to transfer. Details of the list of documents and the redaction procedure are provided in the site manual or equivalent. These documents will be used by the third party solely for the purpose indicated within this protocol.

Study and Site Start and Closure

First Act of Recruitment

The study start date is the date on which the clinical study will be open for recruitment of participants.

The first act of recruitment is the first site open, the date of which will be the study start date.

Study/Site Termination

GSK or designee reserves the right to close the study site or terminate the study at any time for any reason, at the sole discretion of GSK. Study sites will be closed upon study completion. A study site is considered closed when all required documents and study supplies have been collected and a study-site closure visit has been performed.

The Investigator may initiate study-site closure at any time, provided there is reasonable cause and sufficient notice is given in advance of the intended termination.

Reasons for the early closure of a study site by the Sponsor or Investigator may include but are not limited to the following:

For study termination:

- Discontinuation of further study treatment development.

For site termination:

- Failure of the Investigator to comply with the protocol, the requirements of the IRB/IEC or local health authorities, the Sponsor's procedures, and/or GCP guidelines
- Inadequate or no recruitment of participants (evaluated after a reasonable amount of time) by the Investigator
- If the study is prematurely terminated or suspended, the Sponsor shall promptly inform the Investigators, the IECs/IRBs, the regulatory authorities, and any CROs

used in the study of the reason for termination or suspension, as specified by the applicable regulatory requirements. The Investigator shall promptly inform the participant and should assure appropriate participant therapy and/or follow-up.

Publication Policy

- The results of this study may be published or presented at scientific meetings. If this is foreseen, the Investigator agrees to submit all manuscripts or abstracts to the Sponsor before submission. This allows the Sponsor to protect proprietary information and to provide comments.
- The Sponsor will comply with the requirements for publication of study results. In accordance with standard editorial and ethical practice, the Sponsor will generally support publication of multicenter studies only in their entirety and not as individual site data. In this case, a coordinating Investigator will be designated by mutual agreement.
- Authorship will be determined by mutual agreement and in line with International Committee of Medical Journal Editors authorship requirements.

APPENDIX 5. CONTRACEPTIVE AND BARRIER GUIDANCE

It is not known if dostarlimab may have adverse effects on a fetus in utero. However, blockade of PD-L1 signaling in murine models of allogeneic pregnancy can eliminate fetomaternal tolerance and cause spontaneous abortion, as indicated by increase in embryo resorption and a reduction in litter size (D'Addio, Riella et al. 2011).

Therefore, female participants may only be enrolled if they are women of childbearing potential (WOCBP) (see WOCBP definition) who are non-pregnant, non-breastfeeding and are willing to use 1 highly effective form of contraception (Table 20), or are considered women of non-childbearing potential (WONCBP) (see WONCBP definition). Male participants may only be enrolled if they agree to use a male condom (and should also be advised of the benefit for a female partner to use a highly effective method of contraception, as a condom may break or leak) when having sexual intercourse with a WOCBP who is not currently pregnant, or be abstinent from heterosexual intercourse as their preferred and usual lifestyle (abstinent on a long term and persistent basis) and agree to remain abstinent.

Participants should be informed that taking the study medication may involve unknown risks to a fetus (unborn baby) if pregnancy were to occur during the study. In order to participate in the study, they must adhere to the study contraception requirements for the duration of the study and through at least 180 days after the last study treatment (Note: duration of contraceptive use after last dose of chemotherapy must be consistent with local requirements and local approved product labels; however, the minimum duration is 180 days after last dose of chemotherapy). If there is any question that a participant will not reliably comply with the requirements for contraception, that participant should not be entered into the study.

Table 20: Female Contraceptives Allowed During the Study

**FEMALE CONTRACEPTIVES[1] ALLOWED DURING THE STUDY INCLUDE THE FOLLOWING:**
**Highly Effective[2] Methods that Have Low User Dependency** *(Failure rate of <1% per year when used consistently and correctly)*
• Implantable progestogen-only hormone contraception associated with inhibition of ovulation[3]
• IUD
• IUS[3]
• Bilateral tubal occlusion
• Azoospermic partner (vasectomized or due to a medical cause)<br>– Azoospermia is a highly effective contraceptive method, provided that the partner is the sole sexual partner of the WOCBP and the absence of sperm has been confirmed. If not, then an additional highly effective method of contraception should be used. Spermatogenesis cycle is approximately 90 days.<br>**Note:** Documentation of azoospermia for a male participant can come from the site personnel's review of the participant's medical records, medical examination, or medical history interview.
**Highly Effective[2] Methods that Are User Dependent** (*Failure rate of <1% per year when used consistently and correctly)*
• Combined (estrogen- and progestogen-containing) hormonal contraception associated with inhibition of ovulation[3]<br>– oral<br>– intravaginal<br>– transdermal<br>– injectable
• Progestogen-only hormone contraception associated with inhibition of ovulation[3]<br>– oral<br>– injectable

Table 20: Female Contraceptives Allowed During the Study (Continued)

**FEMALE CONTRACEPTIVES[1] ALLOWED DURING THE STUDY INCLUDE THE FOLLOWING:**
**Highly Effective[2] Methods that Are User Dependent (continued)** (*Failure rate of <1% per year when used consistently and correctly)*
• Sexual abstinence<br>– Sexual abstinence is considered a highly effective method only if defined as refraining from heterosexual intercourse during the entire period of risk associated with the study treatment. The reliability of sexual abstinence needs to be evaluated in relation to the duration of the study and the preferred and usual lifestyle of the participant. Periodic abstinence (calendar, symptom-thermal, and post-ovulation methods), withdrawal (coitus interruptus), spermicides only, and lactational amenorrhea method are **not acceptable** methods of contraception.

Abbreviations: CTFG=Clinical Trial Facilitation Group; IUD=intrauterine device; IUS=intrauterine hormone-releasing system' WOCBP=woman of childbearing potential.

1 Contraceptive use by men or women should be consistent with local regulations regarding the use of contraceptive methods for those participating in clinical studies.

2 Failure rate of <1% per year when used consistently and correctly. Typical use failure rates differ from those when used consistently and correctly.

3 Male condoms must be used in addition to hormonal contraception. If locally required, in accordance with CTFG guidelines, then acceptable contraceptive methods are limited to those that inhibit ovulation as the primary mode of action. Male condoms and female condoms should not be used together (due to risk of failure from friction).

Definition of WOCBP

Women in the following categories are considered **WOCBP** (fertile):

1. Following menarche
2. From the time of menarche until becoming post-menopausal unless permanently sterile

Definition of WONCPB

Women in the following categories are considered **WONCBP** (not fertile):

1. Premenopausal female with permanent infertility due to 1 of the following (for the purpose of this study):
   a. Documented hysterectomy
   b. Documented bilateral salpingectomy
   c. Documented bilateral oophorectomy
2. Postmenopausal female

Notes:

- A postmenopausal state is defined as no menses for 12 months without an alternative medical cause.

  - A high follicle-stimulating hormone (FSH) level in the postmenopausal range may be used to confirm a postmenopausal state in women not using hormonal contraception or hormone replacement therapy (HRT). However, in the absence of 12 months of amenorrhea, confirmation with more than 1 FSH measurement is required.
  - Females on HRT and whose menopausal status is in doubt will be required to use 1 of the non-estrogen hormonal highly effective contraception methods if they wish to continue their HRT during the study. Otherwise, they must discontinue HRT to allow confirmation of postmenopausal status before study enrollment.

- For individuals with permanent infertility due to an alternate medical cause other than those previously listed, (e.g., Mullerian agenesis, androgen insensitivity, gonadal dysgenesis), Investigator discretion should be applied when determining study entry.
- If fertility is unclear (e.g., amenorrhea in adolescents or athletes) and a menstrual cycle cannot be confirmed before first dose of study treatment, additional evaluation should be considered.
- Documentation can come from the site personnel's review of the participant's medical records, medical examination, or medical history interview.

APPENDIX 6. LIVER SAFETY REQUIRED ACTIONS AND FOLLOW-UP ASSESSMENTS

Liver chemistry increased monitoring and stopping criteria have been designed to ensure participant safety and evaluate liver event etiology (in alignment with the United States Food and Drug Administration premarketing clinical liver safety guidance) .

Liver chemistry **monitoring criteria** are presented in Table 21.

Table 21: Liver Chemistry Increased Monitoring Criteria

**Criteria**	**Actions**
ALT ≥3×ULN and 1.5×baseline value **but** ALT <5×ULN and 2x baseline value **and** bilirubin <2×ULN**, without** symptoms believed to be related to liver injury, or hypersensitivity **and** who can be monitored weekly for 4 weeks	• If, after 4 weeks of monitoring, ALT <3×ULN and <1.5×baseline value, and bilirubin <2×ULN, monitor participant twice monthly until liver chemistries normalize or return to within baseline

Abbreviations: ALT=alanine aminotransferase; ULN=upper limit of normal.

Liver chemistry **stopping criteria** are presented in Table 22.

Table 22: Liver Chemistry Stopping Criteria

**Laboratory Value**	**Liver Chemistry Stopping Criteria**
**ALT-absolute**	**Both** ALT ≥5×ULN **and** ≥2x baseline value
**ALT Increase**	**Both** ALT ≥3×ULN **and** ≥ 1.5x baseline value persisting for ≥4 weeks
**Bilirubin**[1,2]	ALT ≥3×ULN **and** bilirubin ≥ 2×ULN (>35% direct bilirubin)
**INR**[2]	ALT ≥3×ULN **and** INR>1.5, if INR measured
**Cannot Monitor**	**Both** ALT ≥3×ULN **and** ≥ 1.5x baseline value that cannot be monitored for 4 weeks
**Symptomatic**[3]	**Both** ALT ≥3×ULN **and** ≥ 1.5x baseline value associated with symptoms (new or worsening) believed to be related to liver injury or hypersensitivity

Abbreviations: ALT=alanine aminotransferase; INR=international normalized ratio; SAE=serious adverse event; ULN=upper limit of normal.

[1] Serum bilirubin fractionation should be performed if testing is available. If serum bilirubin fractionation is not immediately available, discontinue study treatment for that participant if ALT ≥3×ULN **and** bilirubin ≥2×ULN. Additionally, if serum bilirubin fractionation testing is unavailable, **record presence of detectable urinary bilirubin on dipstick,** indicating direct bilirubin elevations and suggesting liver injury.

[2] All events of ALT ≥3×ULN **and** bilirubin ≥2×ULN (>35% direct bilirubin) or ALT ≥3×ULN **and** INR>1.5, if INR measured that may indicate severe liver injury (possible 'Hy's Law'), **must be reported as an SAE (excluding studies of hepatic impairment or cirrhosis)**; INR measurement is not required, and the threshold value stated will not apply to participants receiving anticoagulants.

[3] New or worsening symptoms believed to be related to liver injury (such as fatigue, nausea, vomiting, right upper quadrant pain or tenderness, or jaundice) or believed to be related to hypersensitivity (such as fever, rash or eosinophilia).

Liver chemistry **required follow-up assessments** are presented in Table 23.

Table 23: Required Actions and Follow-up Assessments Following ANY Liver Stopping Event

Actions	Follow-up Assessments
**Immediately** discontinue study treatment<br>Report the event to GSK **within 24 hours**<br>Complete the liver event eCRF and complete an SAE data collection tool if the event also meets the criteria for an SAE[3]<br>Perform liver event follow-up assessments<br>Monitor the participant until liver chemistries resolve, stabilize, or return to within baseline<br>**Do not restart/rechallenge** participant with study treatment unless allowed per protocol and GSK Medical Governance approval **is granted.**	• Viral hepatitis serology[1]<br>• Blood sample for PK analysis at the same visit as other chemistry laboratory work or medical evaluation related to the event, preferably within 24 hours of the event[2]<br>• CPK and LDH.<br>• Fractionate bilirubin, if total bilirubin ≥2×ULN<br>• Obtain CBC with differential to assess eosinophilia<br>• Record the appearance or worsening of clinical symptoms of liver injury, or hypersensitivity, on the AE report form<br>• Record use of concomitant medications on the concomitant medications report form including acetaminophen, herbal remedies, and other over-the-counter medications<br>• Record alcohol use on the liver event alcohol intake case report form
**Monitoring – All criteria:**	
Repeat liver chemistries (include ALT, AST, ALP, and bilirubin) and perform liver event follow-up assessments within **24-72 hours**<br>Monitor subjects weekly until liver chemistries resolve, stabilize or return to within baseline.	• Serum acetaminophen adduct HPLC assay (quantifies potential acetaminophen contribution to liver injury in participants with definite or likely acetaminophen use in the preceding week (James, Letzig et al. 2009)).<br>**Note: Not required in China**<br>• Liver imaging (ultrasound, MRI, or CT) and /or liver biopsy to evaluate liver disease; complete Liver Imaging and/or Liver Biopsy eCRF forms.

Table 23: Required Actions and Follow-up Assessments Following ANY Liver Stopping Event (Continued)

Actions	Follow-up Assessments
**Monitoring – Bilirubin or INR criteria:**	
Repeat liver chemistries (include ALT, AST, alkaline phosphatase, bilirubin) and perform liver event follow-up assessments within **24 hours**	• Anti-nuclear antibody, anti-smooth muscle antibody, Type 1 anti-liver kidney microsomal antibodies, and quantitative total IgG or gamma globulins.<br>• Serum acetaminophen adduct HPLC assay (quantifies potential acetaminophen contribution to liver injury in participants with definite or likely acetaminophen use in the preceding week (James, Letzig et al. 2009)).<br>**Note: Not required in China**<br>• Liver imaging (ultrasound, MRI, or CT) and/or liver biopsy to evaluate liver disease; complete Liver Imaging and/or Liver Biopsy eCRF forms.
Monitor participant twice weekly until liver chemistries resolve, stabilize or return to within baseline	
A specialist or hepatology consultation is recommended.	

Abbreviations: AE=adverse event; ALP=alkaline phosphatase; ALT=alanine aminotransferase; AST=aspartate aminotransferase; CBC=complete blood count; CPK=serum creatine phosphokinase; CT=computed tomography; eCRF=electronic case report form; HPLC=high performance liquid chromatography; IgG=immunoglobulin G; IgM=immunoglobulin M; INR=international normalized ratio; LDH=lactate dehydrogenase; MRI=magnetic resonance imaging; PK=pharmacokinetics; SAE=serious adverse event; SRM=study reference manual; ULN=upper limit of normal.

[1] Includes the following: Hepatitis A IgM antibody, hepatitis B surface antigen and hepatitis B core antibody (IgM), hepatitis C RNA, cytomegalovirus IgM antibody, Epstein-Barr viral capsid antigen IgM antibody (or if unavailable, obtain heterophile antibody or monospot testing), and hepatitis E IgM antibody.

[2] PK sample may not be required for participants known to be receiving placebo or non-GSK comparator treatments. Record the date/time of the PK blood sample draw and the date/time of the last dose of study treatment prior to blood sample draw on the eCRF. If the date or time of the last dose is unclear, provide the participant's best approximation. If the date/time of the last dose cannot be approximated OR a PK sample cannot be collected in the time period indicated, do not obtain a PK sample. Instructions for sample handling and shipping are in the SRM.

[3] All events of ALT ≥3×ULN **and** bilirubin ≥2×ULN (>35% direct bilirubin) or ALT ≥3×ULN **and** INR>1.5, if INR measured that may indicate severe liver injury (possible 'Hy's Law'), **must be reported as an SAE (excluding studies of hepatic impairment or cirrhosis)**; INR measurement is not required, and the threshold value stated will not apply to participants receiving anticoagulants.

APPENDIX 7. LIVER SAFETY DRUG RESTART OR RECHALLENGE GUIDELINES

If participant meets liver chemistry stopping criteria do not restart/rechallenge participant with study treatment unless:

- GSK Medical Governance approval **is granted** (as described below),
- Ethics and/or IRB approval is obtained, if required, and
- Separate consent for treatment restart/rechallenge is signed by the participant

If GSK Medical Governance approval to restart/rechallenge participant with study treatment **is not granted**, then participant must permanently discontinue study treatment and may continue in the study for protocol-specified follow up assessments.

Rechallenge Following Liver Stopping Events that are Possibly Related to Study Treatment

Following drug-induced liver injury, **drug rechallenge is associated with a 13% mortality across all drugs in prospective studies** . Clinical outcomes vary by drug, with nearly 50% fatality with halothane re-administered within one month of initial injury. However, some drugs seldom result in recurrent liver injury or fatality.

Risk factors for a fatal drug rechallenge outcome include:

- Hypersensitivity with initial liver injury (eg, fever, rash, eosinophilia)
- Jaundice or bilirubin >2×ULN with initial liver injury (direct bilirubin >35% of total)
- Participant currently exhibits severe liver injury defined by: ALT ≥3×ULN, bilirubin ≥2×ULN (direct bilirubin >35% of total), or INR≥1.5
- SAE or fatality has earlier been observed with drug rechallenges (Papay, Clines et al. 2009, Hunt 2010)
- Evidence of drug-related preclinical liability (e.g.,reactive metabolites, mitochondrial impairment)

Rechallenge refers to resuming study treatment following drug induced liver injury (DILI). Because of the risks associated with rechallenge after DILI this should only be considered for a participant for whom there is compelling evidence of benefit from a critical or life-saving medicine, there is no alternative approved medicine available, and a benefit:risk assessment of rechallenge is considered to be favorable.

Approval by GSK for rechallenge with study treatment can be considered where:

- Investigator requests consideration of rechallenge with study treatment for a participant who is receiving compelling benefit with study treatment that exceeds risk, and no effective alternative therapy is available.
- Ethics Committee or IRB approval for rechallenge with study treatment must be obtained, as required.
- If the rechallenge is approved by GSK Medical Governance in writing, the participant must be provided with a clear description of the possible benefits and risks of study

treatment administration, including the possibility of recurrent, more severe liver injury, or death.

- The participant must also provide signed informed consent specifically for the rechallenge with study treatment. Documentation of informed consent must be recorded in the study chart.
- Study treatment must be administered at the dose specified by GSK.
- Participants approved by GSK Medical Governance for rechallenge with study treatment must return to the clinic twice a week for liver chemistry tests until stable liver chemistries have been demonstrated and then standard laboratory monitoring may resume as per protocol.
- If after study treatment rechallenge the participant meets protocol-defined liver chemistry stopping criteria, study treatment should be permanently discontinued.
- GSK Medical Monitor, and the Ethics Committee or IRB, as required, must be informed of the participant's outcome following study treatment rechallenge.
- GSK must be notified of any adverse events, as per Section 7.5 and Appendix 8.

Restart Following Transient Resolving Liver Stopping Events NOT Related to Study Treatment

Restart refers to resuming study treatment following liver stopping events in which there is a clear underlying cause (other than DILI) of the liver event (e.g.,biliary obstruction, pancreatic events, hypotension, acute viral hepatitis). Furthermore, there should be no evidence of alcoholic hepatitis or hypersensitivity, and the study treatment should not be associated with HLA markers of liver injury.

Approval by GSK for study treatment restart can be considered where:

- Investigator requests consideration for study treatment restart if liver chemistries have a clear underlying cause (e.g., biliary obstruction, hypotension and liver chemistries have improved to normal or are within 1.5 x baseline and ALT <3xULN).
- Restart risk factors (e.g., fever, rash, eosinophilia, or hypersensitivity, alcoholic hepatitis, possible study treatment-induced liver injury or study treatment has an HLA genetic marker associated with liver injury (e.g., lapatinib, abacavir, amoxicillin/clavulanate) are reviewed and excluded
- Ethics Committee or IRB approval of study treatment restart must be obtained, as required.
- If restart of study treatment is approved by GSK Medical Governance in writing, the participant must be provided with a clear description of the possible benefits and risks of study treatment administration, including the possibility of recurrent, more severe liver injury or death.
- The participant must also provide signed informed consent specifically for the study treatment restart. Documentation of informed consent must be recorded in the study chart.

- Study treatment must be administered at the dose specified by GSK.
- Participants approved by GSK Medical Governance for restarting study treatment must return to the clinic once a week for liver chemistry tests until stable liver chemistries have been demonstrated and then laboratory monitoring may resume as per protocol.
- If after study treatment restart the participant meets protocol-defined liver chemistry stopping criteria, follow usual stopping criteria instructions.
- GSK Medical Monitor, and the Ethics Committee or IRB, as required, must be informed of the participant's outcome following study treatment restart.
- GSK must be notified of any adverse events, as per Section 7.5 and Appendix 8.

APPENDIX 8. ADVERSE EVENTS, SERIOUS ADVERSE EVENTS, AND SPECIAL SITUATIONS: DEFINITIONS AND PROCEDURES FOR RECORDING, FOLLOW-UP, EVALUATING, AND REPORTING

Definitions and Criteria

Definition of an Adverse Event

An adverse event (AE) is any untoward medical occurrence in a clinical study participant, temporally associated with the use of a study treatment, whether or not considered related to the study treatment.

Note: An AE can therefore be any unfavorable and unintended sign (including an abnormal laboratory finding), symptom, or disease (new or exacerbated) temporally associated with the use of a study treatment.

Events Meeting the AE Definition

- Any abnormal laboratory test results (hematology, clinical chemistry, or urinalysis) or other safety assessments (e.g., electrocardiogram [ECG], radiological scans, and vital signs measurements), including those that worsen from baseline, considered clinically significant in the medical and scientific judgment of the Investigator (i.e., not related to progression of underlying disease).
- Exacerbation of a chronic or intermittent pre-existing condition including an increase in either frequency or intensity of the condition.
- New conditions detected or diagnosed after study treatment administration even though it may have been present before the start of the study.
- Signs, symptoms, or the clinical sequelae of a suspected intervention-intervention interaction.
- Signs, symptoms, or the clinical sequelae of a suspected overdose of either study treatment or a concomitant medication. Overdose per se will not be reported as an AE/serious adverse event (SAE) unless it is an intentional overdose taken with possible suicidal/self-harming intent. Such overdoses should be reported regardless of sequelae.
- "Lack of efficacy" or "failure of expected pharmacological action" per se will not be reported as an AE or SAE. Such instances will be captured in the efficacy assessments. However, the signs, symptoms, and/or clinical sequelae resulting from lack of efficacy will be reported as AE or SAE if they fulfil the definition of an AE or SAE.

Events NOT Meeting the AE Definition

- Any clinically significant abnormal laboratory findings or other abnormal safety assessments that are associated with the underlying disease, unless judged by the Investigator to be more severe than expected for the participant’s condition.

- The disease/disorder being studied or expected progression, signs, or symptoms of the disease/disorder being studied, unless more severe than expected for the participant's condition.
- Medical or surgical procedure (e.g., endoscopy, appendectomy): the condition that leads to the procedure is the AE.
- Situations in which an untoward medical occurrence did not occur (social and/or convenience admission to a hospital).
- Anticipated day-to-day fluctuations of pre-existing disease(s) or condition(s) present or detected at the start of the study that do not worsen.

Definition of SAE

An SAE is defined as any SAE that, at any dose:

- **Results in death**
- **Is life-threatening**
  - The term 'life-threatening' in the definition of 'serious' refers to an event in which the participant was at risk of death at the time of the event. It does not refer to an event, that hypothetically might have caused death, if it were more severe.
- **Requires inpatient hospitalization or prolongation of existing hospitalization**
  - In general, hospitalization signifies that the participant has been admitted (usually involving at least an overnight stay) at the hospital or emergency ward for observation and/or treatment that would not have been appropriate in the physician's office or outpatient setting. Complications that occur during hospitalization are AE. If a complication prolongs hospitalization or fulfils any other serious criteria, the event is serious. When in doubt as to whether "hospitalization" occurred or was necessary, the AE should be considered serious.
  - Hospitalization for elective treatment of a pre-existing condition that did not worsen from baseline is not considered an AE.
- **Results in persistent or significant disability/incapacity**
  - The term disability means a substantial disruption of a person's ability to conduct normal life functions.
  - This definition is not intended to include experiences of relatively minor medical significance such as uncomplicated headache, nausea, vomiting, diarrhea, influenza, and accidental trauma (e.g., sprained ankle) that may interfere with or prevent everyday life functions but do not constitute a substantial disruption.
- **Is a congenital anomaly/birth defect**
- **Other situations**
  - Medical or scientific judgment should be exercised by the Investigator in deciding whether SAE reporting is appropriate in other situations such as significant medical events that may jeopardize the participant or may require medical or surgical intervention to prevent 1 of the other outcomes listed in the definition.

These events should usually be considered serious. Examples of such events include invasive or malignant cancers, intensive treatment of allergic bronchospasm, blood dyscrasias, convulsions, or development of intervention dependency or intervention abuse.

Definition of a TEAE

A treatment-emergent adverse event (TEAE) is any event that was not present prior to the initiation of study treatment or any event already present that worsens in either intensity or frequency following exposure to study treatment.

Definition of AESI

An adverse event of special interest (AESI) is any AE (serious or nonserious) that is of scientific and medical concern specific to the study treatment, for which ongoing monitoring and rapid communication by the Investigator to the Sponsor are appropriate.

AESIs for this study are as follows:

- Dostarlimab: None

Definition of Cardiovascular Events

Investigators will be required to fill out the specific cardiovascular event page of the electronic case report form (eCRF) for the following AEs and SAEs which are considered cardiovascular events:

- Myocardial infarction/unstable angina
- Congestive heart failure
- Arrhythmias
- Valvulopathy
- Pulmonary hypertension
- Cerebrovascular events/stroke and transient ischemic attack
- Peripheral arterial thromboembolism
- Deep venous thrombosis/pulmonary embolism
- Revascularization

Definitions of Special Situations

- **Abuse:** The persistent or sporadic, intentional excessive use of the study treatment, which is accompanied by harmful physical or psychological effects.
- **Misuse:** The medicinal product is intentionally and inappropriately used not in accordance with the authorized/approved product information.
- **Medication error:** Any preventable incident that may cause or lead to inappropriate study treatment use or participant harm while the study treatment is in the control of the health care professionals or participants. Such incident may be due to health care professional practice, product labeling, packaging and preparation, procedures for administration, and systems, including the following: prescribing, order

communication, nomenclature, compounding, dispensing, distribution, administration, education, monitoring, and use.

- **Overdose:** A deliberate or accidental administration of study treatment to a study participant, at a dose greater than what was assigned to that participant per the study protocol and under the direction of the Investigator.
- **Accidental/occupational exposure:** The unintentional exposure to a study treatment as a result of one's professional or non-professional occupation, or accidental exposure to a non-professional to whom exposure was not intended (i.e., study product given to wrong participant).
- **Suspected unexpected serious adverse reaction (SUSAR):** A serious adverse reaction, the nature and severity of which are not consistent with the information about the medicinal product set out in the Investigator's Brochure (IB) Reference Safety Information (RSI) section.

Recording and Follow-up of AEs and SAEs

- When an AE/SAE occurs, it is the responsibility of the Investigator to review all documentation (e.g., hospital progress notes, laboratory, and diagnostics reports) related to the event.
- The Investigator will then record all relevant AE/SAE information.
- It is **not** acceptable for the Investigator to send photocopies of the participant's medical records to GSK in lieu of completion of the GSK required form.
- There may be instances when copies of medical records for certain cases are requested by GSK. In this case, all participant identifiers, with the exception of the participant number, will be redacted on the copies of the medical records before submission to GSK.
- The Investigator will attempt to establish a diagnosis of the event based on signs, symptoms, and/or other clinical information. Whenever possible, the diagnosis (not the individual signs/symptoms) will be documented as the AE/SAE.

Assessment of Expectedness

Expectedness (whether an AE is "expected" or "unexpected") will be determined by the Sponsor. An AE will be considered unexpected if the nature, severity, or frequency of the event is not consistent with the risk information provided in the RSI for dostarlimab in the IB and for pembrolizumab, pemetrexed, cisplatin, and carboplatin in their respective prescribing information.

Assessment of Intensity

Investigators should assess the severity of AEs according to the National Cancer Institute-Common Terminology Criteria for Adverse Events (NCI-CTCAE) v5.0. In general, NCI-CTCAE severity grades are as follows:

- **Grade 1:** Mild; asymptomatic or mild symptoms; clinical or diagnostic observations only; intervention not indicated; easily tolerated

- **Grade 2:** Moderate; minimal, local, or noninvasive intervention indicated; limiting age-appropriate instrumental activities of daily living (ADLs) (instrumental ADLs refer to preparing meals, shopping for groceries or clothes, using the telephone, or managing money)
- **Grade 3:** Severe or medically significant but not immediately life-threatening; hospitalization or prolongation of hospitalization indicated; disabling; limiting self-care ADL (self-care ADLs refer to bathing, dressing and undressing, feeding self, using the toilet, taking medications, and being not bedridden)
- **Grade 4:** Life-threatening consequences; urgent intervention indicated
- **Grade 5:** Death related to AE

A distinction should be made between **serious** and **severe** AEs. Severity is a measure of intensity, whereas seriousness is defined by the previously stated criteria. For example, a mild degree of gastrointestinal bleeding requiring an overnight hospitalization for monitoring purposes may be considered an SAE but is not necessarily severe. Similarly, an AE that is severe in intensity is not necessarily an SAE. For example, alopecia may be assessed as severe in intensity but may not be considered an SAE.

Assessment of Causality

The Investigator is obligated to assess the relationship between study treatment and each occurrence of each AE/SAE. One of the following categories should be selected based on medical judgment, considering all contributing factors:

- **Related:** A causal relationship between the medicinal product (or study procedures) and AE is a reasonable possibility. For example, the occurrence of the AE cannot be explained by other causative factors. The AE, however, can be explained by the pharmacological effect of the medicinal product such as a similar event having been reported previously, alteration of the dose effect, or the timing or seriousness of the AE. Positive rechallenge/dechallenge is supportive.
- **Not related:** A causal relationship between the medicinal product (or study procedures) and AE is not a reasonable possibility; there is no temporal relationship between the medicinal product and the event, or an alternative etiology is more reasonable.
- A "reasonable possibility" of a relationship conveys that there are facts, evidence, and/or arguments to suggest a causal relationship, rather than a relationship cannot be ruled out.
- The Investigator will use clinical judgment to determine the relationship.
- Alternative causes, such as underlying disease(s), concomitant therapy, and other risk factors, as well as the temporal relationship of the event to study treatment administration will be considered and investigated.
- The Investigator will also consult the IB and/or Product Information, for marketed products, in his/her assessment.

- For each AE/SAE, the Investigator **must** document in the medical notes that he/she has reviewed the AE/SAE and has provided an assessment of causality.
- There may be situations in which an SAE has occurred and the Investigator has minimal information to include in the initial report to GSK. However, **it is very important that the Investigator always assesses causality for every event before the initial transmission of the SAE data to GSK.**
- The Investigator may change his/her opinion of causality in light of follow-up information and may send an SAE follow-up report with the updated causality assessment.
- The causality assessment is one of the criteria used when determining regulatory reporting requirements.

Reporting of SAEs to GSK via Electronic Data Collection Tool

- The primary mechanism for reporting SAE to GSK will be the electronic data collection tool.
- If the electronic system is unavailable, then the site will use the paper SAE data collection tool (see next section) to report the event within 24 hours.
- The site will enter the SAE data into the electronic system as soon as it becomes available.
- After the study is completed at a given site, the electronic data collection tool will be taken off-line to prevent the entry of new data or changes to existing data.
- If a site receives a report of a new SAE from a study participant or receives updated data on a previously reported SAE after the electronic data collection tool has been taken off-line, then the site can report this information on a paper SAE form (see next section) or to the GSK Medical Monitor or the SAE coordinator by telephone.
- Contacts for SAE reporting can be found in the Study Reference Manual.

SAE Reporting to GSK via Paper Data Collection Tool

- Facsimile transmission of the SAE paper data collection tool is the preferred method to transmit this information to the GSK Medical Monitor or the SAE coordinator.
- In rare circumstances and in the absence of facsimile equipment, notification by telephone is acceptable with a copy of the SAE data collection tool sent by overnight mail or courier service.
- Initial notification via telephone does not replace the need for the Investigator to complete and sign the SAE data collection tool within the designated reporting time frames.
- Contacts for SAE reporting can be found in Study Reference Manual.

APPENDIX 9. GENETICS

USE/ANALYSIS OF DNA

Genetic variation may impact a participant's response to study treatment, susceptibility, and severity and progression of disease. Variable response to study treatment may be due to genetic determinants that impact drug absorption, distribution, metabolism, and excretion; mechanism of action of the drug; disease etiology; and/or molecular subtype of the disease being treated. Therefore, where local regulations and Institutional Review Board/Independent Ethics Committee allow, a blood sample will be collected for DNA analysis.

DNA samples will be used for research related to dostarlimab or non-small cell lung cancer (NSCLC) and related diseases. They may also be used to develop tests/assays including diagnostic tests related to dostarlimab (or study treatments of this class) and NSCLC. Genetic research may consist of the analysis of 1 or more candidate genes or the analysis of genetic markers throughout the genome (as appropriate).

Additional analyses of DNA samples may be conducted if it is hypothesized that these may help further understand the clinical data.

The samples may be analyzed as part of a multi-study assessment of genetic factors involved in the response to dostarlimab or study treatments of this class. The results of genetic analyses may be reported in the clinical study report or in a separate study summary.

The Sponsor will store the DNA samples in a secure storage space with adequate measures to protect confidentiality.

The samples will be retained while research on dostarlimab (or study treatments of this class) or NSCLC continues, but no longer than 15 years after the last participant's last visit or other period per local requirements.

APPENDIX 10. ABBREVIATIONS AND DEFINITIONS OF TERMS

The following abbreviations and specialist terms are used in this study protocol.

Table 24: Abbreviations and Specialist Terms

Abbreviation or Specialist Term	Explanation
ADA	anti-drug antibody
ADL	activity of daily living
AE	adverse event
AESI	adverse event of special interest
ALK	anaplastic lymphoma kinase
ALT	alanine aminotransferase
AST	aspartate aminotransferase
BICR	blinded independent central review
BOR	best overall response
BRAF	proto-oncogene B-raf
CD	cluster of differentiation
CFR	Code of Federal Regulations
CI	confidence interval
C_{max}	maximum concentration
C_{min}	minimum concentration
COVID-19	Corona virus disease 2019
CPMS	Clinical Pharmacology Modeling and Simulation
CR	complete response
CRO	contract research organization
CSR	clinical study report
CT	computed tomography
ctDNA	circulating tumor DNA
CTLA-4	cytotoxic T-lymphocyte-associated protein 4
CV	coefficient of variation
DICOM	Digital Imaging and Communications in Medicine
DOR	duration of response
DRE	disease-related event
DRESS	drug reaction with eosinophilia and systemic symptoms
ECG	electrocardiogram

Table 24: Abbreviations and Specialist Terms (Continued)

Abbreviation or Specialist Term	Explanation
ECOG	Eastern Cooperative Oncology Group
eCRF	electronic case report form
EGFR	epidermal growth factor receptor
EORTC-QLQ-C30	European Organisation for Research and Treatment of Cancer Quality of Life Questionnaire 30-item Core Module
EORTC-QLQ-LC13	European Organisation for Research and Treatment of Cancer Quality of Life Questionnaire 13-item Lung Cancer Module
EOT	End-of-Treatment
EU	European Union
FACT-GP5	Functional Assessment of Cancer Therapy-General Population
FDG-PET	fluorodeoxyglucose-positron emission tomography
FFPE	formalin-fixed paraffin-embedded
FSH	follicle-stimulating hormone
GCP	Good Clinical Practice
HR	hazard ratio
HRQoL	health-related quality of life
HRT	hormone replacement therapy
IB	Investigator's Brochure
ICH	International Council for Harmonisation of Technical Requirements for Pharmaceuticals for Human Use
ICF	informed consent form
IEC	Independent Ethics Committee
IHC	immunohistochemistry
IgG	immunoglobulin G
IND	Investigational New Drug
irAE	immune-related adverse event
IRB	Institutional Review Board
ITT	intent-to-treat
IV	intravenous(ly)
LAG-3	lymphocyte-activation gene 3
mAb	monoclonal antibody

Table 24: Abbreviations and Specialist Terms (Continued)

Abbreviation or Specialist Term	Explanation
MedDRA	Medical Dictionary for Regulatory Activities
MRI	magnetic resonance imaging
NCI-CTCAE	National Cancer Institute-Common Terminology Criteria for Adverse Events
NSAID	nonsteroidal anti-inflammatory drug
NSCLC	non-small cell lung cancer
ORR	objective response rate
OS	overall survival
PD	progressive disease
PD-1	programmed cell death protein 1
PD-L1	programmed death-ligand 1
PD-L2	programmed death-ligand 2
PDy	pharmacodynamic
PET	positron emission tomography
PFS	progression-free survival
PGIC	Patient Global Impression of Change
PGIS	Patient Global Impression of Severity
PK	pharmacokinetic(s)
PR	partial response
PRO	patient-reported outcome
PRO-CTCAE	Patient-reported Outcomes Version of the Common Terminology Criteria for Adverse Events
Q3W	every 3 weeks
Q6W	every 6 weeks
Q9W	every 9 weeks
Q12W	every 12 weeks
QTc	corrected QT interval
QTcB	QT interval corrected for heart rate according to Bazett's formula
QTcF	QT interval corrected for heart rate according to Fridericia's formula
QTL	quality tolerance limit
RAP	Reporting and Analysis Plan

Table 24: Abbreviations and Specialist Terms (Continued)

Abbreviation or Specialist Term	Explanation
RECIST	Response Evaluation Criteria in Solid Tumors
ROS-1	receptor tyrosine kinase-1
SAE	serious adverse event
SD	stable disease
SJS	Stevens-Johnson syndrome
SoA	schedule of activities
SUSAR	suspected unexpected serious adverse reaction
T1DM	Type 1 diabetes mellitus
TEAE	treatment-emergent adverse event
TEN	toxic epidermal necrolysis
TIM-3	T-cell immunoglobulin and mucin-domain containing-3
TMB	tumor mutational burden
TPS	tumor proportion score
ULN	upper limit of normal
US	United States
WOCBP	woman of childbearing potential
WONCBP	woman of non-childbearing potential

APPENDIX 11. PROTOCOL AMENDMENT SUMMARIES OF CHANGE – PRIOR VERSIONS

Overall Rationale Amendment 01 (24 July 2020)

CCI [redacted]

Table 25: Summary of Changes for Amendment 1

Section(s) Affected	Description of Change	Brief Rationale
Headers, cover page, and Protocol Amendment Summary of Changes	Headers and cover page were updated with new version number; headers were updated with new document number; Protocol Amendment Summary of Changes section was added per the Sponsor's protocol template as this is the first amendment for the protocol.	CCI [redacted]
Section 1.1. Synopsis, Table 3. Objectives and Endpoints for Study 213403<br><br>Section 1.1. Synopsis, Overall Design<br><br>Section 3. Objectives and Endpoints, Table 6. Objectives and Endpoints for Study 213403<br><br>Section 4.1 Overall Design<br><br>Section 5.1 Inclusion Criteria, #2.<br><br>Section 8.1. Hypothesis	Included BRAF V600E mutation and other genomic aberrations for which an approved targeted therapy is available in the disease eligibility criterion	[redacted]
Section 6.1 Study Treatments Administered, Table 9. Study Treatments for Study 213403	Removed the row for "IMP and NIMP" definitions.<br><br>Changed the "Use" for pembrolizumab from "background intervention" to "comparator".	[redacted]

Amendment 1 DEU-1 (07 January 2021)

This amendment is considered to be non-substantial based on the criteria set forth in Article 10(a) of Directive 2001/20/EC of the European Parliament and the Council of the European Union because it neither significantly impacts the safety or physical/mental integrity of participants nor the scientific value of the study.

Overall Rationale for the Amendment

[redacted]

Summary of Changes for the Amendment

Table 26: Summary of Changes for Amendment 1 DEU-1

Section(s) Affected	Description of Change	Brief Rationale
Headers, cover page, Protocol Amendment Summary of Changes, and Appendix 11. Protocol Amendment History	Headers and cover page were updated with new version number; headers were updated with new document number; Protocol Amendment Summary of Changes section was updated to include rationale for this amendment and administrative information for prior amendment was moved to new Appendix 11. Protocol Amendment History	[redacted]
Section 1.3. Schedule of Activities/Table 4: Schedule of Activities for Study 213403<br><br>Section 6.1.3 Chemotherapy (Pemetrexed, Cisplatin, and Carboplatin)	Amended to correct inaccurate infusion times for chemotherapy agents	
Section 2.2.5.1. Efficacy of Dostarlimab in NSCLC (renamed section; previously, “Dostarlimab in NSCLC”)<br><br>Section 2.2.5.2. Safety of Dostarlimab (new section)	Updated efficacy and safety information for dostarlimab added	

Section(s) Affected	Description of Change	Brief Rationale
Section 5.6. Withdrawal/Stopping Criteria	Reasons for required withdrawal/discontinuation was updated to more explicitly differentiate between scenarios requiring discontinuation and potential scenarios	CCI [redacted]
Section 6.3.2. Blinding	Instructions for emergency unblinding during the course of the study added	
Section 6.4.1. Dose Levels and Dose Adjustment/Table	Instructions for Dose Modifications updated in Table 12<br>Instructions for Dose Modifications updated in Table 13	

Abbreviation: NSCLC=non-small cell lung cancer.

Amendment of Amendment 1 DEU-1 (07 January 2021) to Amendment 3

CCI [redacted]

Summary of Changes for the Amendment

Table 27: Summary of Changes from Amendment 1 DEU-1 (07 January 2021) to Amendment 3 (17 June 2021)

Section(s) Affected	Description of Change	Brief Rationale
Synopsis, Table 3 Objectives and Endpoints<br>Section 3 Objectives and Endpoints, Table 6 Objectives and Endpoints<br>Section 7.7. Pharmacokinetics<br>Section 8.3.1. Analysis Populations<br>Section 8.4.7.3. Statistical Analysis of PK Data<br>Section 8.4.8. Exploratory Immunogenicity Analyses	Clarification that PK and immunogenicity for pembrolizumab will be assessed only if needed	CCI [redacted]
Synopsis, Overall Study Design<br>Synopsis, Table 4 Schedule of Activities<br>Section 7.5.1. Time Period and Frequency for Collecting AE and SAE Information	Correction to timeframe for reporting adverse events (AEs). Rather than all AEs being reported from signing of the informed consent, only treatment-related serious adverse events (SAEs) will reported from signing of the informed consent and all AEs will be reported from the start of study treatment.	[redacted]
Synopsis, Overall Study Design<br>Synopsis, Table 4 Schedule of Activities<br>Section 4.1 Overall Design<br>Section 7.2.2. Baseline Tumor Assessment (Documentation of Target and Non target Lesions)	Baseline tumour assessment of the chest and abdomen should be collected within 35 days prior to first dose, not 28 days.	[redacted]
Synopsis, Overall Study Design<br>Section 4.1 Overall Design<br>Section 7.2.2. Baseline Tumor Assessment (Documentation of Target and Non target Lesions)	Text updated:<br>**The CT component of** positron emission tomography (PET)/CT may be used according to RECIST v1.1 guidelines, with full radiation dose diagnostic CT and IV CT contrast, and as clinically indicated	[redacted]

Section(s) Affected	Description of Change	Brief Rationale
Synopsis, Table 4 Schedule of Activities	Text updated:<br>Treatment cycles are 21 days long with **dosing scheduled** on D1 of each cycle.<br>**All assessments should be done prior to drug administration and within 72 h prior to 1st dose of study drugs administrated in that cycle, unless otherwise specified. (VB: this includes C1D1 and all cycles).**<br>Visits **starting at Cycle 2 can be performed** ±3 days **of the scheduled date**.	CCI [redacted]
Synopsis, Table 4 Schedule of Activities	For archival (or fresh) tumor tissue samples, additional instruction added:<br>**If PD-L1 status is known and the participant has agreed to provide archival sample via the optional consent, this sample may be provided within 28 days of the start of study treatment.**	[redacted]
Synopsis, Table 4 Schedule of Activities	Additional assessment "Evaluate genomic aberration" added	[redacted]
Synopsis, Table 4 Schedule of Activities	Additional instruction added for optional blood sample for genetic testing to clarify sample may be obtained after randomization during first cycle if not collected at screening.	[redacted]
Synopsis, Table 4 Schedule of Activities	PRO collection timepoints updated to reflect timepoints in cycles rather than weeks.	[redacted]
Section 1.3, Table 4 Schedule of Activities	PROs should be collected prior to any clinical procedures, **whenever possible**.	[redacted]

Section(s) Affected	Description of Change	Brief Rationale
Synopsis, Table 4 Schedule of Activities	Clarification added that urinalysis starts at Cycle 6, Day 1	CCI [redacted]
Synopsis, Table 4 Schedule of Activities	Clarification added that urinalysis starts at Cycle 2, Day 1	
Section 2.2.1 Lung Cancer	Removed statement on EU approval of pembrolizumab monotherapy in NSCLC.	
Section 2.2.5.1. Efficacy of Dostarlimab in NSCLC (renamed section; previously, "Dostarlimab in NSCLC")<br>Section 2.2.5.2. Safety of Dostarlimab (new section)	Updated efficacy and safety information for dostarlimab added.	
Section 2.3.1 Risk Assessment, Table 5 Risk Assessment	Add additional detail on the potential risks of exposure to ionizing radiation, due to the inclusion in the study of CT scans.	
Section 5.1 Inclusion Criteria; Section 7.4.7 Pregnancy Testing;<br>Section 7.5.1 Time Period and Frequency for Collecting AE and SAE Information;<br>Section 7.5.5 Pregnancy; and Appendix 5, Contraceptive and Barrier Guidance	The protocol was revised to note the duration of contraception for female and males post chemotherapy must be consistent with local regulations with a minimum of 180 days for males and females, consistent with dostarlimab guidance.	
Section 5.1 Inclusion Criteria	Reworded inclusion criteria 3:<br>~~Target~~ **Measurable** lesions situated in a previously irradiated area ~~are~~ **may be** considered ~~measurable~~ **target lesions** if progression has been demonstrated in such lesions and if there are other target lesions.	

**Section(s) Affected**	**Description of Change**	**Brief Rationale**
Section 5.2 Inclusion Criteria	Reworded exclusion criteria 8:<br>Participant has an additional malignancy or a history of prior malignancy, with the exception of adequately treated basal or squamous skin cancer, cervical carcinoma in situ, ~~or~~ **superficial** bladder ~~carcinoma in situ~~ **cancer** without evidence of disease, **other in situ cancers**, or had a malignancy treated with curative intent and with no evidence of disease recurrence for 5 years since the initiation of that therapy.	CCI [redacted]
Section 5.2 Inclusion Criteria<br>Appendix 3. Clinical Laboratory Tests	Removed the requirement for participants who test positive for the presence of hepatitis B core antibody to be excluded.	[redacted]
Section 5.6. Withdrawal/Stopping Criteria	Reasons for required withdrawal/discontinuation was updated to more explicitly differentiate between scenarios requiring discontinuation and potential scenarios	[redacted]
Section 6.1.3. Chemotherapy (Pemetrexed, Cisplatin, and Carboplatin)	The protocol was amended to provide additional flexibility for local guidelines with relation to infusion times for chemotherapy agents.	[redacted]
Section 7.1.1. General Guidance for Treatment Continuity when Participants are Unable to Come into the Clinic	Instructions for the collection of PROs updated in Table 15.	[redacted]
Section 7.3.1.1. Objective Response Rate	Added definition of best overall response as **confirmed** CR or PR.	[redacted]

Section(s) Affected	Description of Change	Brief Rationale
Section 7.6.1. Biomarker Sample Collection	Add that samples may be used for additional exploratory biomarker testing and for the development of a diagnostic test.	CCI [redacted]
Section 7.11. Biomedical Research	Re-titled from Section 7.11. Future Biomedical Research	
Section 8.4.7.2. Population PK Analysis	Additional text added to describe a potential, future, population PK analysis including data from this study.	
Appendix 1. Guidelines for Assessment of Disease, Disease Progression, and Response Criteria	Amended the use of X-rays for the assessment of measurable lesions to the assessment of progression due to new lesions.	
Appendix 1. Guidelines for Assessment of Disease, Disease Progression, and Response Criteria	Remove the definition of a measurable lesion as ≥10 mm caliper/ruler measurement by clinical examination or medical photography	
Appendix 3. Clinical Laboratory Tests	Added statement to clarify laboratory assessments for each cycle can occur up to 72 hours before treatment	
Appendix 8. Adverse Events, Serious Adverse Events, and Special Situations: Definitions and Procedures for Recording, Follow-up, Evaluating, and Reporting	Removed all references to the eCRF requirement for documenting causality review.	

Amendment 2 (10 September 2020)

Overall Rationale for the Amendment

[redacted]

Summary of Changes for the Amendment

Table 28: Summary of Changes for Amendment 02

Section(s) Affected	Description of Change	Brief Rationale
Headers, cover page, and Protocol Amendment Summary of Changes	Headers and cover page were updated with new version number; headers were updated with new document number; Protocol Amendment Summary of Changes section was updated to include rationale for this version.	[redacted]
Section 1.1 Protocol Summary, Rationale	Corrected typographical error incorrectly capturing GARNET lung cancer population as previously "untreated" NSCLC patients	[redacted]
Section 5.1 Inclusion Criteria; Section 7.4.7 Pregnancy Testing;<br>Section 7.5.1 Time Period and Frequency for Collecting AE and SAE Information;<br>Section 7.5.5 Pregnancy; and Appendix 5, Contraceptive and Barrier Guidance	The protocol was revised to note the duration of contraception for female and males post chemotherapy must be consistent with local regulations with a minimum of 180 days for males and females, consistent with dostarlimab guidance.	[redacted]
Section 6.1.3 Chemotherapy; and 1.3 Schedule of Activities	The protocol was amended to provide additional flexibility for local guidelines with relation to infusion times for chemotherapy agents	[redacted]

Section(s) Affected	Description of Change	Brief Rationale
Section 5.6 Withdrawal/Stopping Criteria; and Section 1.3 Schedule of Activities	Criteria for treatment withdrawal/stopping rules was clarified to include the text "or other adverse event" to the criterion of unacceptable toxicity	CCI [redacted]

Amendment 3 (17 June 2021)

This amendment is considered to be non-substantial based on the criteria set forth in Article 10(a) of Directive 2001/20/EC of the European Parliament and the Council of the European Union because it neither significantly impacts the safety or physical/mental integrity of participants nor the scientific value of the study.

Overall Rationale for the Amendment

CCI [redacted]

Summary of Changes for the Amendment

Table 29: Summary of Changes for Amendment 03

Section(s) Affected	Description of Change	Brief Rationale
Headers, cover page, and Protocol Amendment Summary of Changes	Headers and cover page were updated with new version number; headers were updated with new document number; Protocol Amendment Summary of Changes section was updated to include rationale for this version.	CCI [redacted]

Section(s) Affected	Description of Change	Brief Rationale
Section 1.1 Synopsis, Table 3 Objectives and Endpoints<br>Section 3 Objectives and Endpoints, Table 6 Objectives and Endpoints<br>Section 7.7. Pharmacokinetics<br>Section 8.3.1. Analysis Populations<br>Section 8.4.7.3. Statistical Analysis of PK Data<br>Section 8.4.8. Exploratory Immunogenicity Analyses	Clarification that PK and immunogenicity for pembrolizumab will be assessed only if needed	CCI [redacted]
Section 1.1 Synopsis, Table 3 Objectives and Endpoints<br>Section 3 Objectives and Endpoints, Table 6 Objectives and Endpoints<br>Section 8.4.9. Exploratory Biomarker Analyses	Tumor tissue will be evaluated for PD L1 expression using IHC and may be correlated with ORR **and potentially other clinical endpoints** to treatment	
Section 1.1 Synopsis, Overall Study Design<br>Section 1.3, Table 4 Schedule of Activities<br>Section 4.1 Overall Design<br>Section 7.5.1. Time Period and Frequency for Collecting AE and SAE Information	Correction to timeframe for reporting adverse events (AEs). Rather than all AEs being reported from signing of the informed consent, only treatment-related serious adverse events (SAEs) will reported from signing of the informed consent and all AEs will be reported from the start of study treatment.	
Section 1.1 Synopsis, Overall Study Design<br>Section 1.3, Table 4 Schedule of Activities<br>Section 4.1 Overall Design<br>Section 7.2.2. Baseline Tumor Assessment (Documentation of Target and Non target Lesions)	Baseline tumour assessment of the chest and abdomen should be collected within 35 days prior to first dose, not 28 days.	

**Section(s) Affected**	**Description of Change**	**Brief Rationale**
Section 1.1 Synopsis, Overall Study Design<br>Section 4.1 Overall Design<br>Section 7.2.2. Baseline Tumor Assessment (Documentation of Target and Non target Lesions)	Text updated:<br>**The CT component of** positron emission tomography (PET)/CT may be used according to RECIST v1.1 guidelines, with full radiation dose diagnostic CT and IV CT contrast, and as clinically indicated	CCI [redacted]
Section 1.3, Table 4 Schedule of Activities	Text updated:<br>Treatment cycles are 21 days long with **dosing scheduled** on D1 of each cycle.<br>**All assessments should be done prior to drug administration and within 72 h prior to 1st dose of study drugs administrated in that cycle, unless otherwise specified.**<br>Visits **starting at Cycle 2 can be performed** ±3 days **of the scheduled date**.	
Section 1.3, Table 4 Schedule of Activities	For archival (or fresh) tumor tissue samples, additional instruction added:<br>**If PD-L1 status is known and the participant has agreed to provide archival sample via the optional consent, this sample may be provided within 28 days of the start of study treatment.**	
Section 1.3, Table 4 Schedule of Activities	Additional assessment "Evaluate genomic aberration" added	
Section 1.3, Table 4 Schedule of Activities	Additional instruction added for optional blood sample for genetic testing to clarify sample may be obtained after randomization during first cycle if not collected at screening.	

**Section(s) Affected**	**Description of Change**	**Brief Rationale**
Section 1.3, Table 4 Schedule of Activities	PRO collection timepoints updated to reflect timepoints in cycles rather than weeks.	CCI [redacted]
Section 1.3, Table 4 Schedule of Activities	PROs should be collected prior to any clinical procedures, **whenever possible**.	[redacted]
Section 1.3, Table 4 Schedule of Activities	Clarification added that urinalysis starts at Cycle 6, Day 1	[redacted]
Section 1.3, Table 4 Schedule of Activities	Clarification added that thyroid panel starts at Cycle 2, Day 1	[redacted]
Section 2.2.1 Lung Cancer	Removed statement on EU approval of pembrolizumab monotherapy in NSCLC.	[redacted]
Section 2.2.5.1. Efficacy of Dostarlimab in NSCLC (renamed section; previously, “Dostarlimab in NSCLC”)<br>Section 2.2.5.2. Safety of Dostarlimab (new section)	Updated efficacy and safety information for dostarlimab added.	[redacted]
Section 2.3.1 Risk Assessment, Table 5 Risk Assessment	Add additional detail on the potential risks of exposure to ionizing radiation, due to the inclusion in the study of CT scans.	[redacted]
Section 5.1 Inclusion Criteria	Reworded inclusion criteria 3:<br>~~Target~~ **Measurable** lesions situated in a previously irradiated area ~~are~~ **may be** considered ~~measurable~~ **target lesions** if progression has been demonstrated in such lesions and if there are other target lesions.	[redacted]

Section(s) Affected	Description of Change	Brief Rationale
Section 5.2 Exclusion Criteria	Reworded exclusion criteria 8:<br>Participant has an additional malignancy or a history of prior malignancy, with the exception of adequately treated basal or squamous skin cancer, cervical carcinoma in situ, ~~or~~ **superficial** bladder ~~carcinoma in situ~~ **cancer** without evidence of disease, **other in situ cancers**, or had a malignancy treated with curative intent and with no evidence of disease recurrence for 5 years since the initiation of that therapy.	CCI [redacted]
Section 5.2 Exclusion Criteria 10<br>Appendix 3. Clinical Laboratory Tests	Removed the requirement for participants who test positive for the presence of hepatitis B core antibody to be excluded.	
Section 5.6. Withdrawal/Stopping Criteria	Reasons for required withdrawal/discontinuation was updated to more explicitly differentiate between scenarios requiring discontinuation and other potential scenarios	
Section 6.1.3. Chemotherapy (Pemetrexed, Cisplatin, and Carboplatin)	Pemetrexed should be administered according to the local label, thus guidance on infusion rate has been removed.	
Section 6.3.2. Blinding	Instructions for emergency unblinding during the course of the study added	

Section(s) Affected	Description of Change	Brief Rationale
Section 6.4. Planned Dose Adjustments	Instructions for Dose Modifications for Non-hematological Chemotherapy Toxicities updated in Table 12.<br>Removed the requirement for PD-1 inhibitors be withheld in the event of all drug-related toxicities.<br>Instructions for Dose Modification Guidelines for PD-1 Inhibitor Related AEs updated in Table 13.	CCI [redacted]
Section 7.1.1. General Guidance for Treatment Continuity when Participants are Unable to Come into the Clinic	Instructions for the collection of PROs updated in Table 15.	
Section 7.3.1.1. Objective Response Rate	Added definition of best overall response as **confirmed** CR or PR.	
Section 7.6.1. Biomarker Sample Collection	Add that samples may be used for additional exploratory biomarker testing and for the development of a diagnostic test.	
Section 7.11. Biomedical Research	Re-titled from Section 7.11. Future Biomedical Research	
Section 8.4.7.2. Population PK Analysis	Additional text added to describe a potential, future, population PK analysis including data from this study.	
Appendix 1. Guidelines for Assessment of Disease, Disease Progression, and Response Criteria	Amended the use of X-rays for the assessment of measurable lesions to the assessment of progression due to new lesions.	

Section(s) Affected	Description of Change	Brief Rationale
Appendix 1. Guidelines for Assessment of Disease, Disease Progression, and Response Criteria	Remove the definition of a measurable lesion as ≥10 mm caliper/ruler measurement by clinical examination or medical photography	CCI [redacted]
Appendix 3. Clinical Laboratory Tests	Added statement to clarify laboratory assessments for each cycle can occur up to 72 hours before treatment	[redacted]
Appendix 8. Adverse Events, Serious Adverse Events, and Special Situations: Definitions and Procedures for Recording, Follow-up, Evaluating, and Reporting	Removed all references to the eCRF requirement for documenting causality review.	[redacted]

Amendment 4 (30 November 2021)

This amendment is considered to be non-substantial based on the criteria set forth in Article 10(a) of Directive 2001/20/EC of the European Parliament and the Council of the European Union because it neither significantly impacts the safety or physical/mental integrity of participants nor the scientific value of the study.

Overall Rationale for the Amendment 04

CCI [redacted]

Summary of Changes for the Amendment 04

Table 30: Summary of Changes for Amendment 04

Section(s) Affected	Description of Change	Brief Rationale
Section 1.1 Synopsis - Treatment Groups and Duration<br>Section 4.1 Overall Design - Treatment Groups and Duration<br>Section 8.2 Sample Size Considerations	Removed number of participants limit per strata	CCI [redacted]
Section 1.3 Schedule of Activities (Table 4)	Post dosing timepoints for PK and Biomarker sample collection is done after immunotherapy infusion.	
Section 6.4.2.1. Management of PD-1 Inhibitor-related AEs (Table 13: Dose Modification Guidelines for PD-1 Inhibitor-related AEs)	Removed superscript	
	"Permanently discontinue" for Grade 4 severity was removed from adrenal insufficiency, hypophysitis, hypothyroidism and hyperthyroidism.	

Section(s) Affected	Description of Change	Brief Rationale
	• Updated dose modifications for adrenal insufficiency to include endocrine consultation as follow-up.<br>• Expanded dose modifications for Guillain-Barré syndrome to include several severe neurological events (myasthenic syndrome/myasthenia gravis and transverse myelitis were added), to include Grade 2, to include corticosteroid and other therapy guidelines, and to include neurology consultation as follow-up.<br>• Updated dose modifications for myocarditis.<br>• Added DRESS to dose modifications for rash/skin reactions.<br>• Added corticosteroid and other therapy guidelines and consultation follow-up for Other irARs.	CCI [redacted]
	Removed the incorrect footnote that the decision to withhold or discontinue PD-1 inhibitor is at the discretion of the Investigator or treating physician.	

<u>Appendix 30</u>: FRENCH-SPECIFIC REQUIREMENTS

This appendix includes all applicable requirements of French Public Health Code / specific local GSK requirements and identifies, item per item, the mandatory modifications or additional information to the study protocol.

1. Concerning the « SELECTION OF STUDY POPULATION AND WITHDRAWAL CRITERIA»

- A subject will be eligible for inclusion in this study if he/she is either affiliated to or beneficiary of a social security category (French Public Health Code law L.1121-8-1) (exception for a participant to a non-interventional study if authorised by the Ethics Committee).

  It is the investigator's responsibility to ensure and to document (in the source document - subject notes) that the subject:
  - is either affiliated to or beneficiary of a social security category.
  - has got an authorisation by the Ethics Committee.

2. Concerning the "STATISTICAL CONSIDERATIONS AND DATA ANALYSES" and specially in the "SAMPLE SIZE ASSUMPTIONS"

The expected number of subjects to be recruited in France is declared to the French regulatory authority.

3. Concerning the "STUDY GOVERNANCE CONSIDERATIONS"

- **In section "Regulatory and Ethical Considerations, including the Informed Consent Process"**

  ⇒ Concerning **the process for informing the subject** and/or his/her legally authorized representative, the following text is added:

  French Patient Informed Consent is a document which summarizes the main features of the study and allows collection of the subject and/or his/her legally authorized representative written consent. It also contains a reference to the authorisation of ANSM and the approval from the French Ethics Committee.

  ⇒ **Concerning the management of the Patient Informed Consent Forms**, the following text is added:

  French Patient Informed Consent Form is in duplicate (triplicate for minor subject).

  The first page of the Patient Informed Consent Form is given to the investigator. The copy is kept by the patient or legally authorized representative.

- **NOTIFICATION TO THE HOSPITAL DIRECTOR**

  In accordance with Article L1123-13 of the French Public Health Code, the Hospital Director is informed of the commitment to the trial in her/his establishment. The Hospital Director is supplied with the protocol and any information needed for the financial disposition, the name of the investigator(s), the number of sites involved in his establishment and the estimated time schedule of the trial (R.1123-69).

- **INFORMATION TO THE HOSPITAL PHARMACIST**

  In accordance with Article R.1123-70 of the French Public Health Code, the Hospital Pharmacist is informed of the commitment to the trial in her/his establishment. The Pharmacist is supplied with a copy of the protocol (which allows her/him to dispense the drug(s) of the trial according to the trial methodology), all information concerning the product(s) of the trial (e.g. included in the CIB), the name of the investigator(s), the number of sites involved in her/his establishment and the estimated time schedule of the trial.

4. Concerning the " DATA MANAGEMENT " the following text is added:

- Within the framework of this clinical trial, data regarding the identity of the investigators and/or co-investigators and/or the pharmacists if applicable, involved in this clinical trial, and data regarding the subjects recruited in this clinical trial (subject number, treatment number, subjects status with respect to the clinical trial, dates of visit, medical data) will be collected and computerized in GlaxoSmithKline data bases by GlaxoSmithKline Laboratory or on its behalf, for reasons of follow up, clinical trial management and using the results of said clinical trial. According to the Data Protection French Law n° 78-17 of 6th January 1978 updated, each of these people aforesaid has a right of access, correction and opposition on their own data through GlaxoSmithKline Laboratory (Clinical Operations Department).

- **Ethnic Origin**

  In accordance with the Data Protection French Law n° 78-17 of 6th January 1978 updated – article 4-3, the ethnic origin can only be collected if the collection of this data is strictly necessary and relevant for the purpose of the study.

  There are numerous examples throughout the study of human medicines where collection of demographic information including gender, age, race, and ethnicity have helped to better understand differences in clinical pharmacology, efficacy, or safety of newly developed drugs. For example, the presence of epidermal growth factor receptors (EGFR) is predictive of sensitivity to treatment with EGFR tyrosine kinase inhibitors (TKIs) and is a significant determining factor for treatment selection in NSCLC. Based on previous research data, the frequency of EGFR mutations can vary significantly by region, race and ethnicity.

  Although participants with known EGFR mutations are excluded from participation in Study 213403, there may be other types of genetic and ethnic variation that may similarly impact the efficacy or safety of the therapeutic agents under study.

  Study 213403 will compare treatment with two different PD-1 inhibitors (pembrolizumab versus dostarlimab) in combination with chemotherapy in participants with newly diagnosed metastatic non squamous NSCLC. The study has a non-inferiority design. As a result, it is critical to the validity of our results that the populations in both arms be as similar as possible. The collection of race/ethnicity data in study 213403 will help to ensure this homogeneity by allowing us to better understand any imbalances in demographic features between the arms that could potentially skew our results. Doing so will also allow for investigation of potential emerging differences in efficacy or safety between these combinations. In addition, samples may be assessed for pharmacogenomic factors and pharmacodynamic receptor occupancy, to help characterize the PK/PDy and pharmacogenetic profile of the therapeutic agents. Collection of race/ethnic data may be helpful to further elucidate any differences in these characteristics, which may have an impact on efficacy or safety.

  Furthermore, it is intended to use the results of this study to support potential future regulatory submissions and anticipate receiving specific requests from regulatory authorities, in the US and elsewhere, for race specific safety, pharmacokinetic, and efficacy data.

- **TESTING OF BIOLOGICAL SAMPLES**
  In accordance with the French Public Health Code law – article L1211-2, a biological sample without identified purpose at the time of the sample and subject's preliminary information is not authorized.

5. Monitoring visits

The Health Institution and the Investigator agree to receive on a regular basis a Clinical Research Assistant (CRA) of GLAXOSMITHKLINE or of a service provider designated by GLAXOSMITHKLINE. The Health Institution and the Investigator agree to be available for any phone call and to systematically answer to all correspondence regarding the Study from GLAXOSMITHKLINE or from a service provider designated by GLAXOSMITHKLINE. In addition, the Health Institution and the Investigator agree that the CRA or the service provider designated by GLAXOSMITHKLINE have direct access to all the data concerning the Study (test results, medical record, etc …). This consultation of the information by GLAXOSMITHKLINE is required to validate the data registered in the electronic Case Report Form (eCRF), in particular by comparing them directly to the source data. In accordance with the legal and regulatory requirements, the strictest confidentiality will be respected.

6. Data entry into the eCRF

The Health Institution and the Investigator agree to meet deadlines, terms and conditions of the Study's electronic Case Report Form (eCRF) use here below:

The Health Institution and the Investigator undertake:

1. That the Investigator and the staff of the investigator center make themselves available to attend the training concerning the computer system dedicated to the electronic Case Report Form (eCRF) of the Study provided by GLAXOSMITHKLINE or by a company designated by GLAXOSMITHKLINE.
2. That the Investigator and the staff of the investigator center use the IT Equipment loaned and/or the access codes only for the purpose of which they are intended and for which they have been entrusted to them, namely for the Study achievement, to the exclusion of any other use.
3. That the Investigator and the staff of the investigator center use the IT Equipment loaned according to the specifications and manufacturer's recommendations which will have been provided by GLAXOSMITHKLINE.
4. To keep the IT Equipment and/or access codes in a safe and secure place and to authorize only the use of this IT Equipment by investigator center staff designated by the principal investigator to enter the data of the Study.
5. To be responsible for the installation and payment of the required Internet connections needed for the use of the IT Equipment, Computer systems and/or access codes.
6. To return at the end of the Study the IT Equipment and/or access codes to GLAXOSMITHKLINE or to any company designated by GLAXOSMITHKLINE and any training material and documentation. The IT Equipment cannot under any circumstances be kept by the Health Institution or the Investigator for any reason whatsoever.

7. CTR publication

It is expressly specified that GLAXOSMITHKLINE and/or the Sponsor can make available to the public the results of the Study by the posting of the said results on a website of the GLAXOSMITHKLINE GROUP named Clinical Trial Registred (CTR) including the registration of all the clinical trials conducted by the GLAXOSMITHKLINE Group and this before or after the publication of such results by any other process.

8. Data Protection French Law of 6th January 1978 updated (CNIL)

In accordance with the Data Protection French Law of 6 January 1978 updated, personal data are processed in a manner that ensures appropriate security, including protection against unauthorized or unlawful processing and against accidental loss, destruction or damage, using appropriate technical or organizational measures. The processing is whether deemed to be compliant with one of the methodology of reference **(MR-001)** or has been the subject of a request for authorization to the CNIL. The Investigator has, regarding the processing data related to her/him, a right of access, of rectification, erasure and of opposition with GLAXOSMITHKLINE in accordance with the legal provisions.

9. Investigational Product Accountability, Reconciliation, and Destruction

In specific situations (if the site has 1- an approval from the French Regulatory Agency (ANSM) and 2- a GSK written endorsement letter) where institutional practices dictate that the site disposes of and/or destroys IP prior to allowing the "monitor' to verify and document IP accountability, the following applies:

*"During the conduct of the Study, Investigational Product (IP) will be destroyed by the Institution prior to a GlaxoSmithKline "**monitor**" conducting final investigational product accountability. Institution agrees that such destruction will comply with Institution's investigational product accountability procedures and will provide GlaxoSmithKline with investigational product accountability logs and supporting documentation to verify adherence to 'Bonnes Pratiques Cliniques' (decision dated on the 24th of November 2006).*

Signature Page for RPS-CLIN-050714 v2.0

Approval	PPD<br>I am signing for the reasons as stated in the document.<br>14-Feb-2023 21:46:10 GMT+0000

Signature Page for RPS-CLIN-050714 v2.0
